# Genome‐ and epigenome‐wide studies of plasma protein biomarkers for Alzheimer's disease implicate TBCA and TREM2 in disease risk

**DOI:** 10.1002/dad2.12280

**Published:** 2022-04-20

**Authors:** Robert F. Hillary, Danni A. Gadd, Daniel L. McCartney, Liu Shi, Archie Campbell, Rosie M. Walker, Craig W. Ritchie, Ian J. Deary, Kathryn L. Evans, Alejo J. Nevado‐Holgado, Caroline Hayward, David J. Porteous, Andrew M. McIntosh, Simon Lovestone, Matthew R. Robinson, Riccardo E. Marioni

**Affiliations:** ^1^ Centre for Genomic and Experimental Medicine Institute of Genetics and Cancer, University of Edinburgh Edinburgh UK; ^2^ Department of Psychiatry University of Oxford Oxford UK; ^3^ Centre for Clinical Brain Sciences, Chancellor's Building, 49 Little France Crescent University of Edinburgh Edinburgh UK; ^4^ Edinburgh Dementia Prevention, Centre for Clinical Brain Sciences University of Edinburgh Edinburgh UK; ^5^ Lothian Birth Cohorts, Department of Psychology University of Edinburgh Edinburgh UK; ^6^ MRC Human Genetics Unit Institute of Genetics and Cancer University of Edinburgh Edinburgh UK; ^7^ Division of Psychiatry, Centre for Clinical Brain Sciences University of Edinburgh Edinburgh UK; ^8^ Neurodegeneration Johnson and Johnson Medical Ltd Wokingham UK; ^9^ Medical Genomics Group Institute of Science and Technology Austria Klosterneuburg Austria

## Abstract

**Introduction:**

The levels of many blood proteins are associated with Alzheimer's disease (AD) or its pathological hallmarks. Elucidating the molecular factors that control circulating levels of these proteins may help to identify proteins associated with disease risk mechanisms.

**Methods:**

Genome‐wide and epigenome‐wide studies (n_individuals_ ≤1064) were performed on plasma levels of 282 AD‐associated proteins, identified by a structured literature review. Bayesian penalized regression estimated contributions of genetic and epigenetic variation toward inter‐individual differences in plasma protein levels. Mendelian randomization (MR) and co‐localization tested associations between proteins and disease‐related phenotypes.

**Results:**

Sixty‐four independent genetic and 26 epigenetic loci were associated with 45 proteins. Novel findings included an association between plasma triggering receptor expressed on myeloid cells 2 (TREM2) levels and a polymorphism and cytosine‐phosphate‐guanine (CpG) site within the *MS4A4A* locus. Higher plasma tubulin‐specific chaperone A (TBCA) and TREM2 levels were significantly associated with lower AD risk.

**Discussion:**

Our data inform the regulation of biomarker levels and their relationships with AD.

## 1 INTRODUCTION

Alzheimer's disease (AD) is one of the leading causes of disease burden and death globally.^1^, ^2^ Blood‐based methods for assessing disease risk are potentially more cost‐effective and less invasive than neuroimaging methods or lumbar punctures for collecting cerebrospinal fluid (CSF). Approaches that use genomics and untargeted proteomics have suggested that there are signals in blood that can supplement targeted assays, and contribute to the understanding and prediction of AD.^3^, ^4^ However, the relevance of many candidate protein markers identified by untargeted approaches to AD remains unclear.^5^, ^6^ Understanding the molecular factors that regulate the levels of AD‐associated proteins may identify proteins that bear relevance to disease risk mechanisms.

Unlike genetic factors, which remain largely stable over the life‐course, differential DNA methylation (DNAm) profiles are influenced by genetic and non‐genetic factors.^7^ DNAm is characterized by the addition of methyl groups to DNA, typically in the context of cytosine‐phosphate‐guanine (CpG) nucleotide base pairings. Clusters of CpG sites termed CpG islands are located near 70% of gene promoters. CpG island methylation is typically associated with reduced gene expression. However, it is important to note that DNAm is dynamic, tissue‐specific, and cell‐specific.^8^ DNAm data may capture independent information beyond genetic factors in explaining inter‐individual variation in circulating protein levels. Several genome‐wide association studies (GWAS) have catalogued polymorphisms associated with plasma protein levels and identified proteins that correlate with risk scores for various disease states including AD.^9^, ^10^, ^11^ Zaghlool et al. (2020) performed the only large‐scale epigenome‐wide association study (EWAS) to date on plasma protein levels (>1000 proteins).^12^ Few studies have combined GWAS and EWAS data to quantify the independent and combined contributions of genetic and epigenetic factors toward differential protein biomarker levels.^13^, ^14^, ^15^

We performed a structured literature review of studies that report associations between plasma proteins and AD diagnosis or related traits such as amyloid beta (Aβ) burden and cortical atrophy.^16^, ^17^, ^18^, ^19^, ^20^, ^21^, ^22^, ^23^, ^24^, ^25^, ^26^, ^27^ We focused on studies that measured plasma protein levels using the SOMAscan affinity proteomics platform (SomaLogic Inc.), as this matches the protocol used in our study, Generation Scotland. We identified 282 proteins that were also measured in our sample (*n* ≤ 1064). Our first aim was to conduct GWAS and EWAS on plasma levels of 282 AD‐associated proteins. Using Bayesian penalized regression, we estimated the proportion of inter‐individual variability in plasma protein levels that can be accounted for by variation in genetic and DNAm factors. BayesR+ implicitly adjusts for probe intercorrelations and data structure, including relatedness.^28^ For our second aim, we used Mendelian randomization (MR) and co‐localization analyses to test for relationships between plasma protein levels and AD phenotypes.

## 2 METHODS

### 2.1 Study cohort

Analyses were performed using blood samples from participants of the **ST**ratifying **R**esilience **a**nd **D**epression **L**ongitudinally (STRADL) cohort, comprising 1188 individuals from the larger, family‐structured Generation Scotland: the Scottish Family Health Study (GS). GS consists of 24084 individuals from across Scotland. Recruitment for GS took place between 2006 and 2011. STRADL participants partook in follow‐up data collection 4 to 13 years after baseline.^29^, ^30^

### 2.2 Search strategy

We searched MEDLINE (Ovid interface, Ovid MEDLINE in‐process and other non‐indexed citations and Ovid MEDLINE 1946 onwards), Embase (Ovid interface, 1980 onwards), Web of Science (core collection, Thomson Reuters), and medRxiv/bioRxiv to identify relevant articles indexed as of May 28, 2021. Search terms are outlined under supplementary information. Twenty‐five articles were identified and one further article was identified through a supplemental manual literature search. After removal of duplicates, 23 articles were assessed for inclusion criteria: (1) original research article, (2) proteins were measured in plasma, (3) proteins were measured using SOMAscan technology, and (4) proteins were associated with AD or related phenotypes. Twelve articles met inclusion criteria.

### 2.3 Protein quantification

The 5k SOMAscan v4 array was used to quantify the levels of plasma proteins in GS participants (*n* = 1065). This highly multiplexed platform uses chemically modified aptamers termed SOMAmers (**S**low **O**ff‐rate **M**odified **A**pta**mers**) that recognize epitopes on their cognate protein targets with high specificity and high affinity in the nanomolar‐to‐picomolar range. The recognition signal is measured as relative fluorescence units (RFUs) on microarrays.

Plasma samples were collected in 150 μL aliquots and stored at −80°C. Samples were run in 96‐well plates and reagents were spread across three dilution factors (0.005%, 0.5%, and 20%) to create distinct sets for high, medium, and low abundance proteins, respectively. Raw microarray data were normalized through a number of quality control steps, which are detailed in the supplementary information. After quality control and the exclusion of non‐human proteins, deprecated markers and spuriomers, 4235 SOMAmers were retained for proteomic analyses.

Normalized RFUs (from SomaLogic) were log‐transformed and regressed onto the following covariates: age, sex, study site (Aberdeen/Dundee), time between sample being collected and processed for proteomics (factor, 4 levels), and 20 genetic principal components (PCs) of ancestry from multidimensional scaling (to control for population structure). Relationships between covariates and SOMAmers are shown in Table S1. Residualized RFUs were transformed by rank‐based inverse normalization. We refer to these as protein levels; however, they reflect RFUs that have undergone a number of quality control, transformation and pre‐correction steps.

**RESEARCH IN CONTEXT**

1. Systematic Review: The authors performed a structured literature review of studies that reported associations between SOMAscan‐measured plasma proteins and Alzheimer's disease (AD). Twelve studies were included following a search of MEDLINE, Embase, Web of Science, and preprint servers. The goal of the study was to combine genome‐wide and epigenome‐wide association studies (GWAS and EWAS) with causal modeling methods to investigate associations between plasma proteins and AD risk. The study used data from the Scottish population‐based cohort, Generation Scotland.
2. Interpretation: Two hundred eighty‐two proteins across the included studies were available for testing in Generation Scotland. Seven novel genetic and 19 novel cytosine‐phosphate‐guanine (CpG) sites were associated with plasma levels of 18 proteins. Higher plasma levels of tubulin‐specific chaperone A (TBCA) and triggering receptor expressed on myeloid cells 2 (TREM2) associated with lower risk of AD.
3. Future Directions: Triangulation of evidence across other experimental and epidemiological approaches will be necessary to determine if blood proteins influence AD risk.

### 2.4 GWAS

Generation Scotland samples were genotyped using the Illumina Human OmniExpressExome‐8v1.0 Bead Chip and processed using the Illumina Genome Studio software v2011 (Illumina, San Diego, CA, USA). Quality control steps are outlined under supplementary information. After quality control, 561125 single nucleotide polymorphisms (SNPs) remained for 1064 individuals. In total, 1064 individuals had both genotype and proteomic data available for analyses.

Bayesian penalized regression GWAS were performed using BayesR+ software in C++.^28^ BayesR+ utilizes a mixture of prior Gaussian distributions to allow for markers with effect sizes of different magnitudes. It also includes a discrete spike at zero that enables the exclusion of markers with non‐identifiable effects on the trait of interest. Guided by data from our previous studies, mixture variances for the stand‐alone GWAS were set to 0.01 and 0.1 to allow for markers that account for 1% or 10% of variation in circulating protein levels, respectively.^14^, ^15^ In the combined GWAS/EWAS analysis, genotype and DNAm data must have had the same number of prior variances (*n* = 3 each). Mixture variances for SNP data were set to 0.01, 0.1, and 0.2 in combined analyses. Input data were scaled to mean zero and unit variance. Gibbs sampling was used to sample over the posterior distribution conditional on input data and 10000 samples were used. The first 5000 samples of burn‐in were removed and a thinning of five samples was applied to reduce autocorrelation. SNPs that exhibited a posterior inclusion probability (PIP) ≥95% were deemed significant.

### 2.5 EWAS

Blood DNAm in Generation Scotland participants was quantified using the Illumina HumanMethylationEPIC BeadChip Array. Blood DNAm was assessed in two separate sets. After quality control, 793706 and 773860 CpG remained in sets 1 and 2, respectively. In total, 772619 CpG sites were shared across sets. Each set was truncated to these overlapping probes.

In the stand‐alone EWAS and combined GWAS/EWAS, mixture variances were set to 0.001, 0.01, and 0.1 (n = 778). Missing DNAm data were mean imputed separately within each set as BayesR+ cannot accept missing values. Both sets were combined and adjusted for DNAm batch, set, age, and sex. Each CpG site was scaled to mean zero and unit variance. Houseman‐estimated white blood cell proportions were included as fixed‐effect covariates.^31^ CpG sites that had a PIP ≥95% were deemed significant.

Sensitivity EWAS analyses were performed using linear mixed‐effects models and the lmekin function from the R *coxme* package (version 2.2‐16).^32^ DNAm data were pre‐corrected for age, sex, batch, and set. Houseman‐estimated white blood cell proportions were incorporated as fixed‐effect covariates and a kinship matrix was fitted to account for relatedness among individuals in STRADL.

### 2.6 Co‐localization analyses

Formal Bayesian tests of co‐localization were used to determine whether a shared causal variant likely underpinned two traits of interest.^33^ A 200 kilobase (kb) region (upstream and downstream, recommended default setting) surrounding the variant was extracted from our GWAS summary statistics.

Expression quantitative trait loci (eQTL) data were extracted from eQTLGen summary statistics. Methylation QTL (mQTL) summary statistics were extracted from phenoscanner, GoDMC, or our own mQTL analyses. Methylation QTL analyses were performed using additive linear regression models and by regressing CpG sites (beta values) on SNPs (0, 1, 2) while adjusting for age, sex, DNAm batch, set, Houseman‐estimated white blood cell proportions, and 20 genetic PCs (*n* = 778). In instances where an mQTL effect was identified in more than one database, summary statistics from the study with the largest sample size were used in *coloc*.^34^, ^35^, ^36^ For AD‐related traits, summary statistics were extracted from the relevant GWAS.^3^, ^37^, ^38^, ^39^ Default priors were applied. Summary statistics for all SNPs (± 200 kb from the queried SNP) were used to estimate the posterior probability for five separate hypotheses: a single variant underlying both traits, separate variants for both traits, a causal variant for one trait (encompassing two hypotheses), or no causal variant for either trait. Posterior probabilities ≥95% provided strong evidence for a given hypothesis.

### 2.7 Mendelian randomization (MR)

Bidirectional Mendelian randomization (MR) was used to test for associations between (1) gene expression and plasma protein levels, (2) DNAm and plasma protein levels, and (3) plasma protein levels and AD risk or related biomarkers. Pruned variants (*r* ^2 ^< 0.1) were used as instrumental variables (IVs) in MR analyses. Analyses were conducted using MR‐base.^40^ Two‐sample MR was applied and relationships were assessed using the Wald ratio test. Further information on IVs used are provided in supplementary information.

## 3 RESULTS

### 3.1 Identification of plasma proteins associated with AD

**FIGURE 1:**
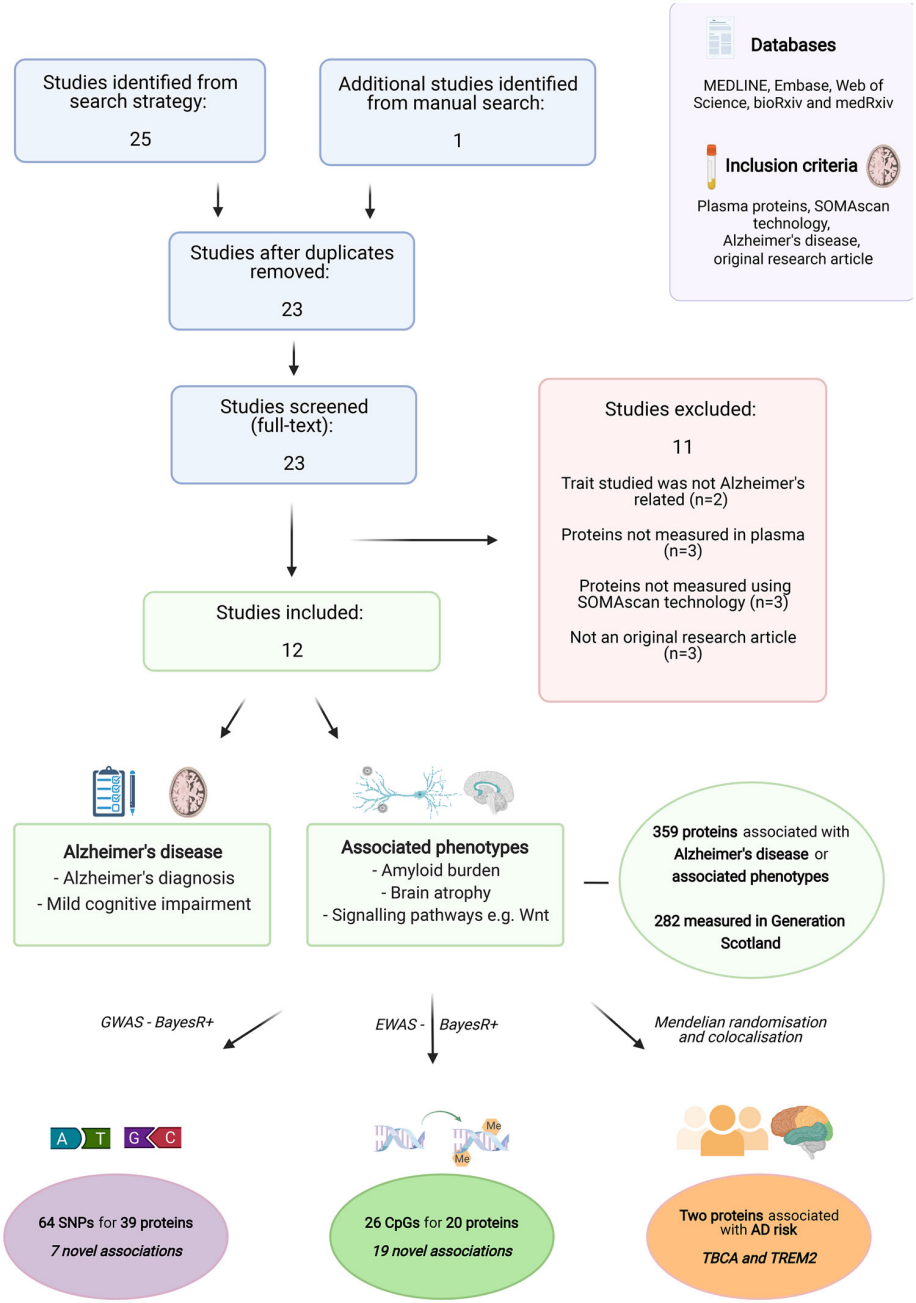
Structured literature review of SOMAscan plasma proteins that were associated with AD in the literature, and assessment of their molecular architectures and relationships with AD in the present study. The MEDLINE, Embase, Web of Science databases, and preprint servers were queried to identify studies that reported associations between SOMAscan‐measured plasma proteins and AD. GWAS, EWAS, and causal inference analyses were performed to identify molecular correlates of 282 AD‐associated plasma protein levels and to probe their associations with AD and related traits. AD, Alzheimer's disease; EWAS, epigenome‐wide association studies; GWAS, genome‐wide association studies. Figure created using Biorender.com

Twelve studies were identified that reported associations between SOMAscan plasma proteins and AD or related traits (Figure 1). Three hundred fifty‐nine unique proteins were identified and 22 (6.1%) were reported in more than one study (Table S2‐S4). In the Generation Scotland dataset, there were 308 SOMAmers (**S**low **O**ff‐rate **M**odified **A**pta**mers**) that targeted 282 of 359 proteins of interest (Table S5 and Figure S1). The 282 unique proteins were brought forward for analyses (UniProt IDs and Seq‐ids are shown in Table S6).

### 3.2 GWAS on AD‐associated proteins

**FIGURE 2:**
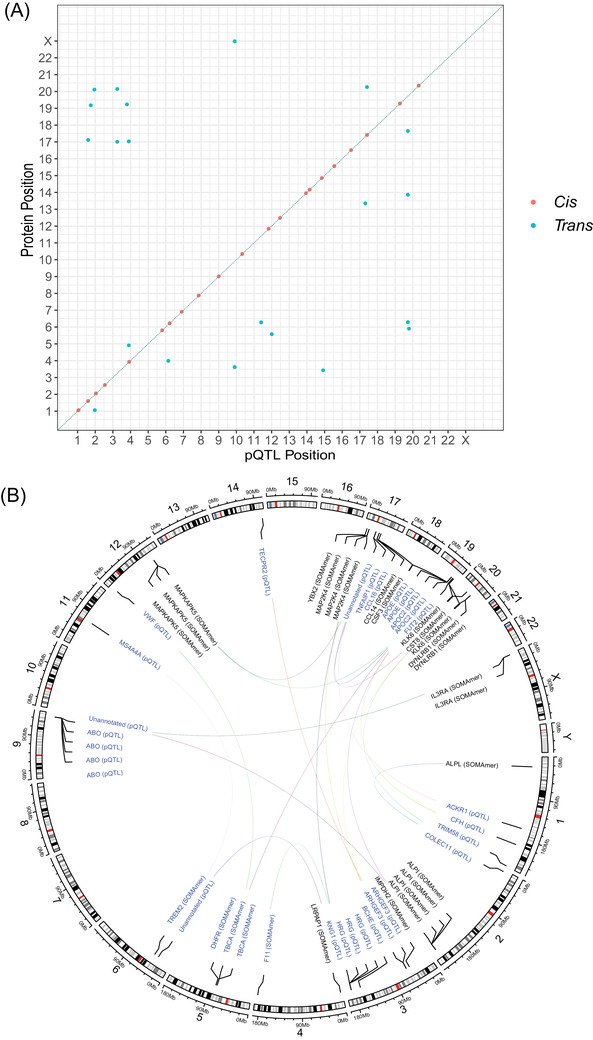
GWAS on plasma protein levels previously associated with AD and disease‐related phenotypes. (A) Chromosomal locations of pQTLs identified through Bayesian penalized regression GWAS. The *x*‐axis shows the chromosomal location of pQTLs associated with the levels of SOMAmers that correlate with AD status or related pathways. The *y*‐axis represents the position of the gene encoding the target protein. *Cis* (red circles); *trans* (blue circles). (B) A circos plot for the 28 *trans*‐associated pQTLs from (A). Lines indicate an association between a pQTL and SOMAmer. AD, Alzheimer's disease;GWAS, genome‐wide association studies; pQTL, protein quantitative trait locus

There were 1064 individuals with genotype and proteomic data in Generation Scotland. The mean age of the sample was 59.9 years (standard deviation [SD] = 5.9) and 59.1% of the sample was female. In the BayesR+ GWAS, 64 independent variants (or protein quantitative trait loci, pQTLs) were associated with 41 SOMAmers that mapped to 39 unique protein targets (PIP≥ 95%; Table S7). The phenotypic correlation structure of these 41 SOMAmers is presented in Figure S2. The median correlation coefficient between SOMAmer levels was 0.18. Thirty‐six pQTLs represented *cis* associations (pQTLs within 10 megabases [Mb] of transcription start site [TSS] for a given gene) and 28 pQTLs were *trans‐*chromosomal effects (Figure 2). The majority of variants were located in intronic regions using annotations from the ENSEMBL variant effect predictor (46.9%, Figure S3).

Fifty‐seven pQTLs were previously reported in GWAS of blood protein levels (Table S8). Variants either directly replicated known associations or showed high linkage disequilibrium (LD, *r* ^2 ^> 0.75) with known pQTLs for queried proteins. Relative effect sizes reported in the literature correlated strongly with those in our study (*r *= 0.77, 95% confidence interval [CI] = 0.66, 0.84). We identified seven novel pQTLs associated with seven unique proteins. Three pQTLs were in *cis* (for GM2A, MATN3 and IL1RAP). Four pQTLs represented *trans‐*chromosomal effects: rs1126680 (*BCHE* for KLK6), rs7867739 (near *ABO* for ALPI), rs3820897 (*COLEC11* for ALPL), and rs1530914 (*MS4A4A* for triggering receptor expressed on myeloid cells 2 [TREM2]).

Thirty‐three pQTLs were associated with at least one trait in the GWAS Catalog at *P* < 5 × 10^−8^ (range = 1 to 96 associations; Table S9).^41^ In relation to AD traits, the *trans* pQTL in *MS4A4A* (rs1530914) for TREM2 levels is in high LD with a *TREM2* variant (rs1582763, *r* ^2^ ∼ 0.9) associated with AD in apolipoprotein E (*APOE*) ε4 carriers and family history of AD.^3^, ^42^ In addition, the *trans* pQTL in *APOE* (rs769449) for tubulin‐specific chaperone A (TBCA) levels was associated with 15 AD‐related traits including genetic predisposition to AD and CSF biomarkers of the disease.

BayesR+ was used to estimate the proportions of inter‐individual variation in plasma protein levels that were attributable to common SNPs (minor allele frequency >1%). Estimates ranged from 5.3% (PRL; 95% credible interval [CrI] = 0%, 24.4%) to 73.0% (IL1RAP; 95% CrI = 56.0%, 83.0%), with a median estimate of 13.0% across all 308 SOMAmers (Table S10).

### 3.3 Co‐localization of protein QTLs with expression QTLs

The 36 *cis* pQTLs identified in BayesR+ were annotated to 23 unique proteins. For 12 of 23 proteins, at least one pQTL was previously reported to be an expression QTL for the respective gene in blood tissue (eQTL consortium database).^34^ The R package *coloc* ^33^ was used to test the hypothesis that a single variant associates with differences in gene expression (eQTL) and protein levels (pQTL) for each gene of interest. For two proteins (PCSK7 and F7), there was strong evidence (posterior probability [PP]) >95%) for a shared variant underlying gene expression and protein levels (Table S11). MR analyses provided evidence for reciprocal associations between changes in gene expression and circulating levels of these proteins (Table S12). Three proteins had weaker evidence for co‐localization (PP ≥75% for GM2A, LYZ, PDCD1LG2) and seven proteins had strong evidence for separate variants underlying gene expression and protein levels.

### 3.4 EWAS on AD‐associated proteins

**FIGURE 3:**
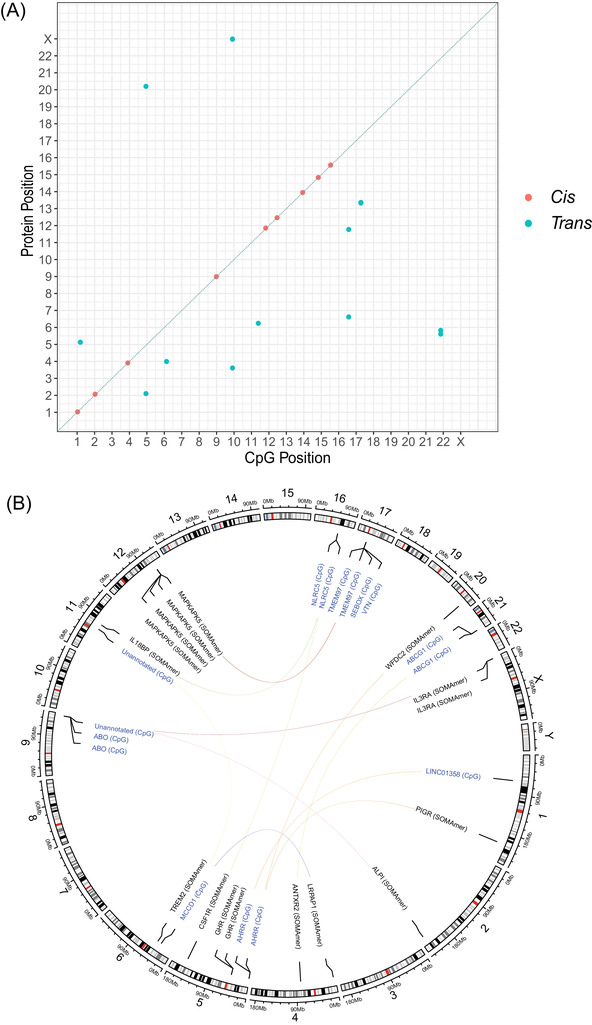
EWAS on plasma protein levels previously associated with AD and disease‐related phenotypes. (A) Chromosomal locations of CpGs identified through Bayesian penalized regression EWAS. The *x*‐axis shows the chromosomal location of CpG sites and the *y*‐axis represents the position of the gene encoding the target protein. *Cis* (red circles); *trans* (blue circles). (B) A circos plot for the 16 *trans*‐associated CpGs from (A). Lines indicate an association between a CpG site and SOMAmer. AD, Alzheimer's disease; CpG, cytosine‐phosphate‐guanin ; EWAS, epigenome‐wide association studies

There were 778 individuals with DNAm and proteomic data in the Generation Scotland sample. The mean age of the sample was 60.2 (SD = 8.8) years and 56.4% of the sample were female. Twenty‐six CpGs were associated with the levels of 20 unique proteins (PIP >95%, Table S13 and Figure S4). The median correlation coefficient between measured protein levels was 0.16. The associations consisted of 10 *cis* CpG sites and 16 *trans* CpG loci (Figure 3). The cg07839457 probe in the *NLRC5* locus was associated with IL18BP and CSF1R levels, and the smoking‐associated probe cg05575921 in *AHRR* was associated with GHR, PIGR, and WFDC2 levels.

We used linear mixed‐effects models that accounted for relatedness to perform sensitivity analyses for the 26 CpG associations identified in BayesR+ (Table S14).^32^ Effect sizes were highly correlated with those from BayesR+ and showed full directional concordance (*r *= 0.95, 95% CI = 0.90, 0.98; Figure S5). Twenty‐one associations were replicated at a genome‐wide significance threshold of *P* < 3.6 x 10^−8^,and the remaining five associations were replicated at *P* < 2.0 × 10^−3^. Furthermore, 7 of 26 CpG associations were previously reported in the literature and relative effect sizes correlated strongly with those in our study (*r *= 0.98, 95% CI = 0.87, 1.0). The 19 novel CpG sites were associated with levels of 14 unique proteins.

In BayesR+, estimates for the proportions of variability in SOMAmer levels that could be accounted for by DNAm measured on the EPIC BeadChip array ranged from 7.1% (EEA1; 95% CrI = 0%, 27.7%) to 33.8% (MAP kinase‐activated protein kinase, MAPKAPK5; 95% CrI = 22.6%, 47.0%), with a median estimate of 10.0% (Table S15).

Estimates for variance in SOMAmer levels accounted for by genetic and methylation data together, while conditioned on each other, ranged from 21.8% for ENTPD1 (95% CrI = 0.0%, 59.1%) to 93.3% for GHR (95% CrI = 80.1%, 100%) (Table S16). The mean and median estimates were 48.7% and 46.8%, respectively.

### 3.5 Co‐localization of protein QTLs with methylation QTLs

Fourteen proteins had both genome‐wide significant pQTL and CpG associations in our study. There were 39 possible SNP‐CpG pairs across these proteins. For each pair, we used linear regression to test if the SNP was associated with CpG methylation at *P* < 5 × 10^−8^, thereby representing an mQTL effect (Table S17). We also performed look‐up analyses of mQTL databases including GoDMC and phenoscanner.^35^, ^36^ In instances where an mQTL effect was identified in more than one database, coefficients from the study with the largest sample size were brought forward for co‐localization analyses. In addition, in instances where two or more mQTLs were associated with the same CpG site in a given locus, only the most significant mQTL was brought forward for co‐localization analyses (*n* = 19 mQTLs, 13 proteins; Table S18).

For six proteins, we observed strong evidence in *coloc* that a single *cis*‐acting variant might underpin differential DNAm levels and protein abundances (PP >95%, Table S19). The six proteins were ANXA2, F7, MATN3, PCSK7, PLA2G2A, and SERPINA3. MR analyses provided evidence that relationships between methylation and protein levels were bidirectional (Table S20).

### 3.6 MR analyses between plasma proteins and AD risk

Bidirectional MR was applied to test for associations between the 41 SOMAmers with pQTL associations in BayesR+ and 20 AD‐related traits (Table S21). A Bonferroni‐corrected threshold of *P* < 6.10 × 10^−5^ (< 0.05/41 × 20) was set. Plasma levels of three proteins had a unidirectional association with AD risk: TREM2 (Table 1, Wald ratio test, beta = −0.13, SE = 0.05, *P* = 8.4 × 10^−17^), colony stimulating factor 3 (CSF3) (Wald ratio test, beta = 0.10, SE = 0.02, *P* = 5.9 × 10^−6^), and TBCA (inverse variance‐weighted method, beta = ‐0.50, SE = 0.12, *P* = 1.2 × 10^−5^). Conversely, AD risk was not associated with plasma levels of these proteins. Co‐localization analyses suggested that one variant was associated with TREM2 or TBCA levels and AD risk, and two separate variants were associated with CSF3 levels and AD risk (Table S22).

**TABLE 1:** MR analyses of plasma protein levels and AD‐associated traits (Bonferroni‐corrected *P* < 6.10 × 10^−5^)

Protein	Trait	Method	Beta	SE	*P*	Reference
***Protein levels affecting AD‐associated traits***						
TBCA	Log‐transformed CSF Aβ42	IVW	−0.09	0.01	2.5 × 10^−17^	^38^
TREM2	AD risk	Wald ratio	−0.13	0.02	8.4 × 10^−17^	^3^
TBCA	CSF APOE	Wald ratio	0.75	0.10	7.3 × 10^−14^	^39^
TBCA	CSF Aβ (Z‐scores)	IVW	−0.45	0.06	2.1 × 10^−13^	^38^
TBCA	Log‐transformed CSF Aβ42/Aβ40	IVW	−0.08	0.01	6.9 × 10^−10^	^38^
CSF3	AD risk	Wald ratio	0.10	0.02	5.9 × 10^−6^	^3^
TBCA	AD risk	IVW	−0.50	0.12	1.2 × 10^−5^	^3^
***AD‐associated traits affecting protein levels***						
TBCA	Log‐transformed CSF Aβ42	Wald ratio	−11.14	0.53	4.4 × 10^−98^	^38^
TBCA	CSF Aβ (Z‐scores)	Wald ratio	−2.13	0.10	5.7 × 10^−98^	^38^
TBCA	Log‐transformed CSF Aβ42/Aβ40	Wald ratio	−11.13	0.53	5.7 × 10^−98^	^38^
TBCA	CSF Aβ	Wald ratio	12.21	0.63	3.7 × 10^−84^	^37^

Abbreviations: CSF, cerebrospinal fluid; IVW, inverse variance‐weighted method; MR, mendelian randomization; SE, standard error.

## 4 DISCUSSION

We identified seven novel protein QTLs and 19 novel CpG sites that associated with plasma levels of 18 AD‐related proteins. Using BayesR+, we provided estimates for associations between common genetic and DNAm variation and inter‐individual differences in plasma levels of 282 AD‐related proteins. We integrated our data with publicly available gene expression and methylation QTL databases and highlighted molecular mechanisms that might link pQTLs to differential levels of six proteins. We observed strong associations between plasma levels of TREM2 or TBCA and AD risk. These associations were driven by *trans* pQTLs in *MS4A4A* and *APOE*, respectively.

We show that a *trans* pQTL (rs1530914) in the *MS4A4A* locus associates with higher plasma TREM2 levels. It is in strong LD (*r* ^2^ ∼ 0.9) with the variant rs1582763, which has been associated with higher CSF TREM2 levels and lower AD risk.^3^, ^43^ It is also in moderate LD (*r* ^2^ = 0.6) with a variant in the 3′UTR region of *MS4A6A* (rs610932), which was associated with plasma TREM2 levels in a sample of 35,559 Icelanders.^11^ Polymorphisms in *MS4A4A* were shown to alter *MS4A4A* expression and subsequently modulate TREM2 concentration in human macrophages.^44^ We supplement existing data by identifying a novel blood CpG correlate of plasma TREM2 levels (cg02521229) located near *MS4A4A* that previously associated with dementia risk in Generation Scotland participants.^45^ Our data suggest that risk mechanisms arising from *MS4A4A* polymorphisms and TREM2 levels can be captured in plasma assays and that these mechanisms involve differential methylation in the *MS4A4A* locus.

We observed associations between plasma levels of three proteins (CSF3, MAPKAPK5, and TBCA) and *trans* pQTLs in the *TOMM40‐APOE‐APOC2* locus. Furthermore, we identified two pQTLs and three CpG correlates of plasma MAPKAPK5 levels near the transmembrane protein 9 *(TMEM97)*locus. MAPKAPK5 correlated with cognitive decline in the Twins UK cohort; however, its relationship with neuropathology is unknown.^22^ TMEM97 acts a synaptic receptor for Aβ and mediates its cellular update via *APOE*‐dependent and *APOE*‐independent mechanisms.^46^ Given that *TMEM97* polymorphisms may influence MAPKAPK5 levels, our data prioritize MAPKAPK5 for follow‐up studies as a potential downstream effector or correlate of TMEM97 in Aβ clearance. TBCA correlates with Aβ burden.^16^ TBCA levels are higher in individuals with the protective *APOE* ε2/ε2 genotype and lower in carriers of the risk ε4 polymorphism.^47^ These data are consistent with our GWAS and MR analyses. Future studies should examine whether TBCA dysregulation is a cause or consequence of disease risk mechanisms in carriers of *APOE* ε4 polymorphisms.

Our study has a number of limitations. First, our review does not reflect an exhaustive list of potential AD‐associated traits. Furthermore, there is heterogeneity across studies in terms of diagnostic criteria and phenotype definitions. Second, by focusing on the SOMAscan platform alone, we do not capture all blood protein correlates of AD that are reported in the literature. Third, an insufficient number of variants were available to test for horizontal pleiotropy in MR analyses. Fourth, it is important to note that variants may alter SOMAmer reactivity with protein targets, or reflect technical artifacts such as sample handling and cross‐reactive events. Fifth, our sample consisted of Scottish individuals with a relatively homogenous genetic background thereby limiting generalizability of findings.

## 5 CONCLUSIONS

Our strategy of integrating multiple omics measures determined the degree to which molecular factors can explain inter‐individual differences in blood levels of possible biomarkers for AD, and advanced understanding of mechanisms underlying AD risk.

## CONFLICT OF INTEREST

Andrew M. McIntosh has received research support from Eli Lilly, Janssen, and the Sackler Foundation. Andrew M. McIntosh has also received speaker fees from Illumina and Janssen and consulting fees. Simon Lovestone is currently an employee of Johnson & Johnson Medical Ltd and previously received grant support from multiple pharmaceutical companies and the EU through the Innovation Medicines Initiative programmes European Medical Information Framework and European Prevention of Alzheimer's Dementia (EPAD). Simon Lovestone is also co‐founder of Akrivia Health. Alejo J. Nevado‐Holgado has received funding from GlaxoSmithKline, UK, Ono Pharma, Japan, and Johnson & Johnson, UK. David J. Porteous is PI on a grant from Wellcome made to the University of Edinburgh with travel allowance. Craig W. Ritchie has been a paid consultant for several companies developing treatments for Alzheimer's disease over the last 5 years including Biogen, Eli Lilly, Merck, Roche, Janssen, AbbVie, Kyowa Kirin, Actinogen, and Eisai. Craig W. Ritchie was the UK Chief Investigator for the ENGAGE Trial and Academic Lead on the EPAD Programme, which was a public‐private partnership between the EU and several companies with an interest in developing treatments for AD (www.ep‐ad.org). Craig W. Ritchie's unit at the University of Edinburgh (Edinburgh Dementia Prevention) has received grant funding from Biogen, Janssen, AC Immune, and Actinogen; he is the unpaid chairperson of the Brain Health Clinic Consortium established in the UK by Biogen; was a member of a Data Safety Monitoring Board (DSMB) for a University College London (UCL) sponsored study (no payment); and serves as Director of Brain Health Scotland and Chair of the Scottish Dementia Research Consortium. Ian J. Deary has received royalties from Oxford University Press and Cambridge University Press. Archie Campbell was a member of the EMREC Edinburgh Medical Research Ethics Committee (no payment involved). Riccardo E. Marioni has received speaker fees from Illumina and is an advisor to the Epigenetic Clock Development Foundation. The remaining authors declare that they have no competing interests.

## ETHICS STATEMENT

All components of the Generation Scotland study received ethical approval from the NHS Tayside Committee on Medical Research Ethics (REC Reference Numbers: 05/S1401/89 and 10/S1402/20). All participants provided broad and enduring written informed consent for biomedical research. Generation Scotland has also been granted Research Tissue Bank status by the East of Scotland Research Ethics Service (REC Reference Number: 20‐ES‐0021). This study was performed in accordance with the Declaration of Helsinki.

## CODE AVAILABILITY

All code is available at the following Github repository.^50^

## Supporting information



SUPPORTING INFORMATION

SUPPORTING INFORMATION

SUPPORTING INFORMATION

SUPPORTING INFORMATION

## Data Availability

According to the terms of consent for Generation Scotland participants, access to data must be reviewed by the Generation Scotland Access Committee. Applications should be made to access@generationscotland.org. Full and openly accessible summary statistics from GWAS and EWAS on SOMAmer levels are available on the University of Edinburgh Datashare site.^48^, ^49^
