# Supplementary figures and images for: Genome‐ and epigenome‐wide studies of plasma protein biomarkers for Alzheimer's disease implicate TBCA and TREM2 in disease risk
Source: Alzheimers Dement (Amst). 2022 Apr 20;14(1):e12280. doi: 10.1002/dad2.12280 (PMC9019629; doi:10.1002/dad2.12280)

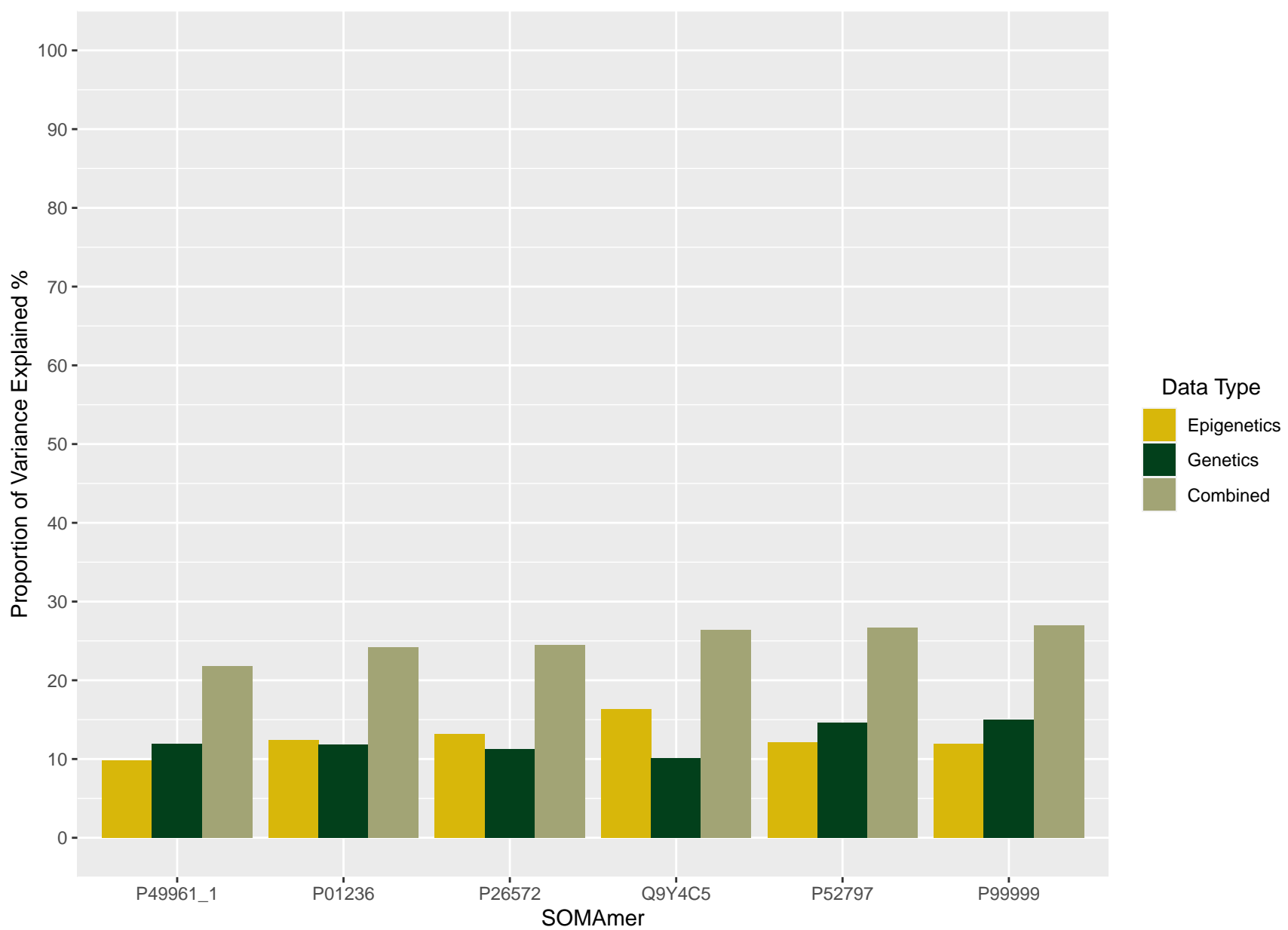

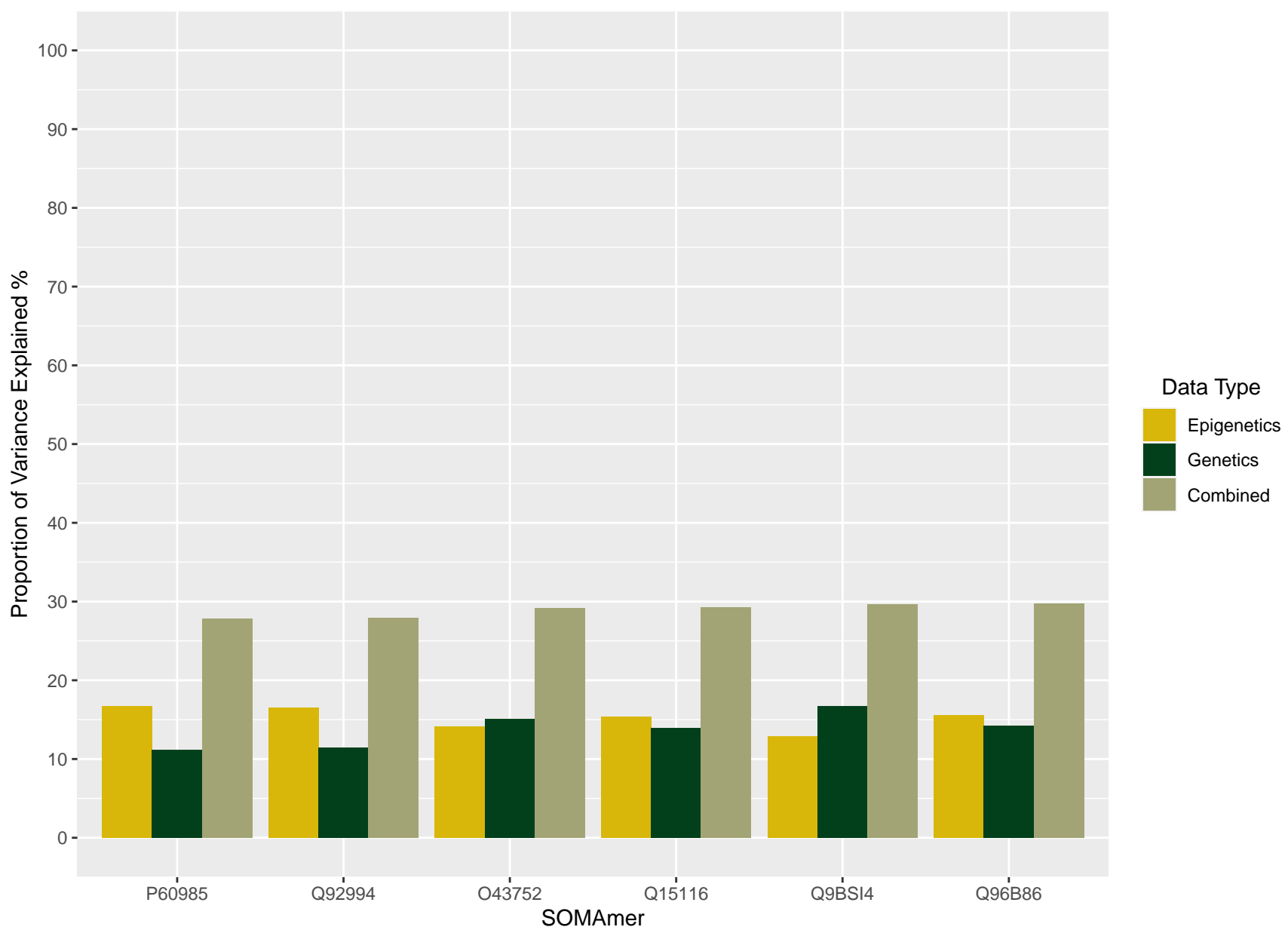

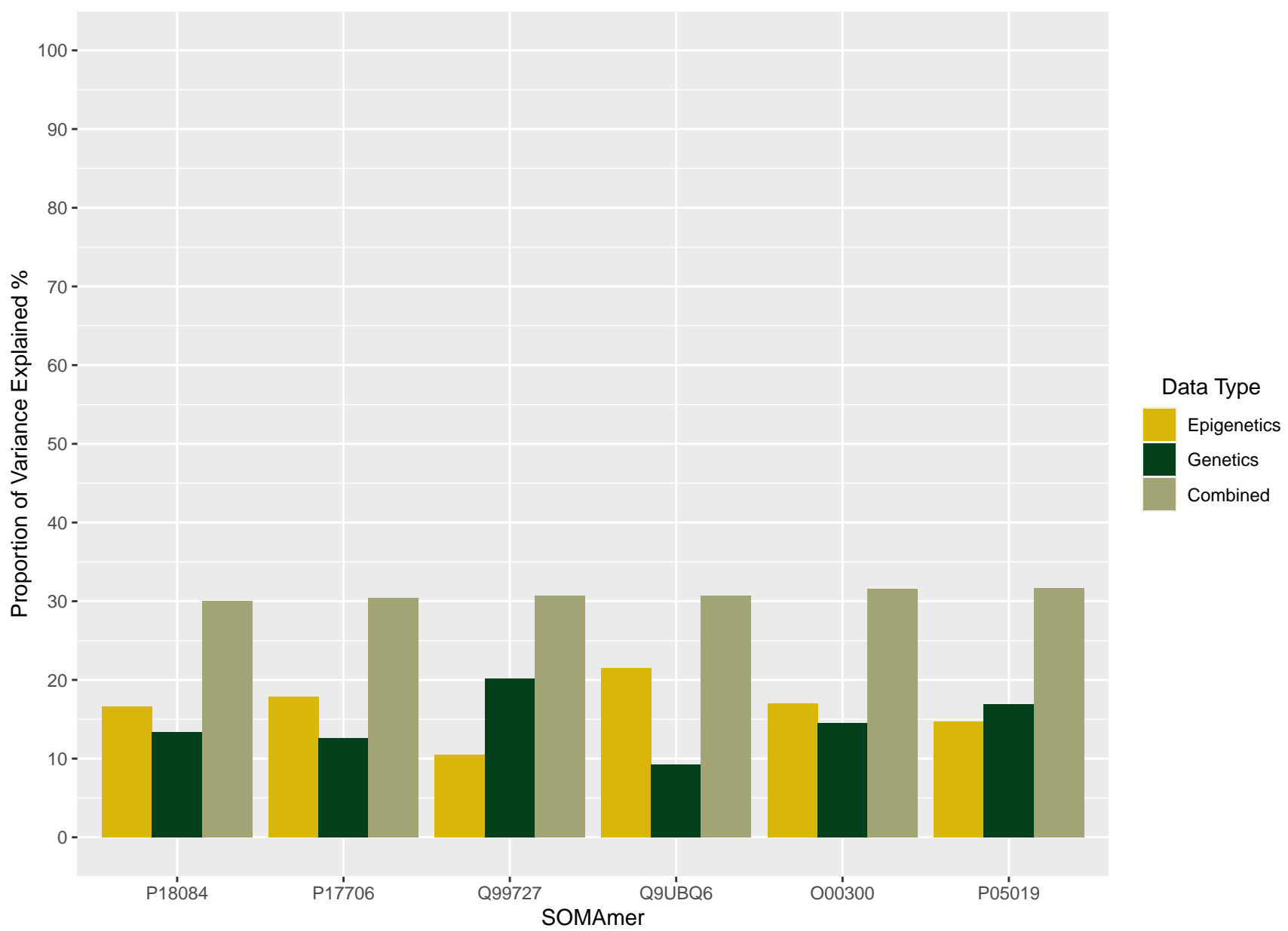

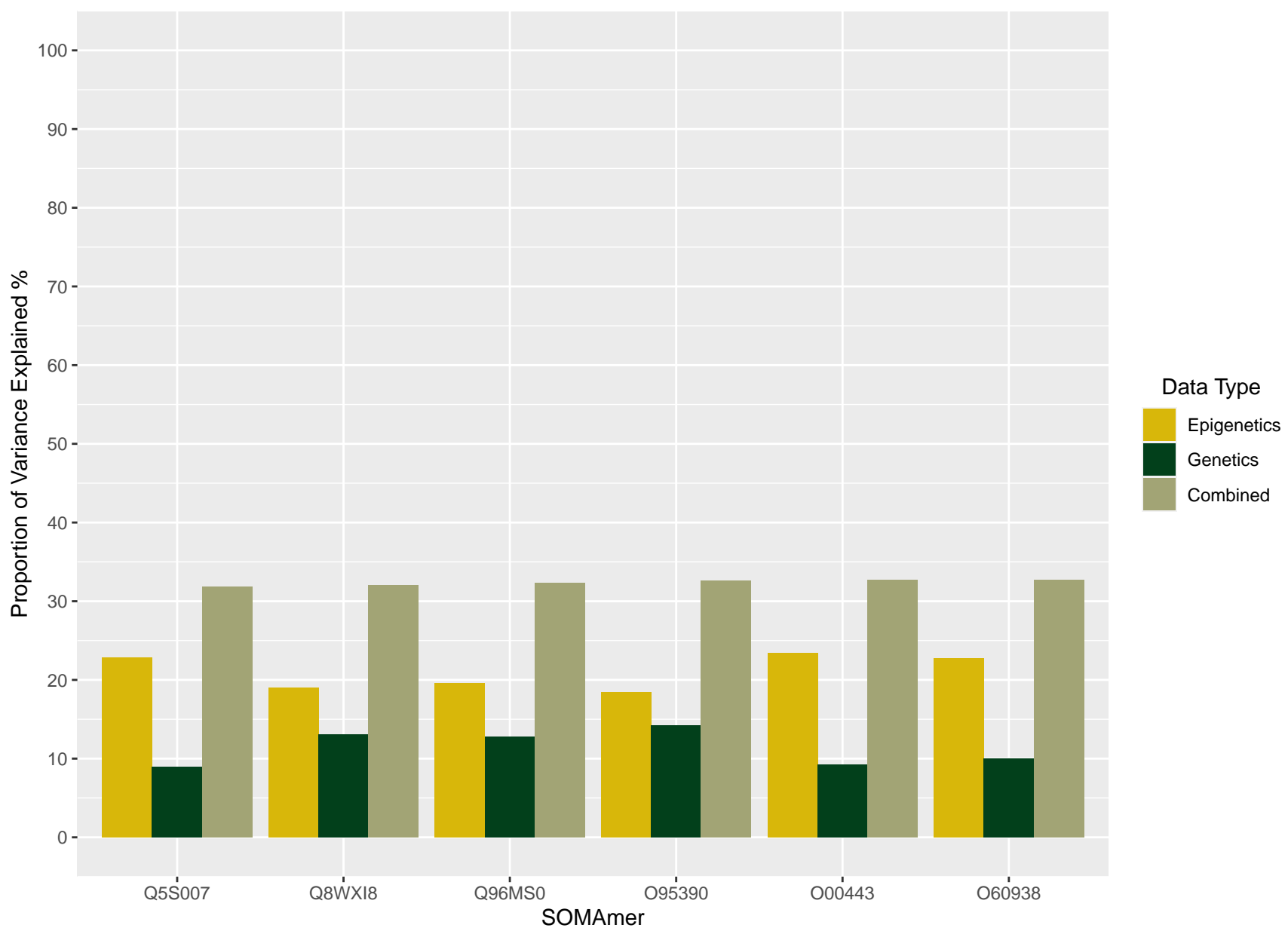

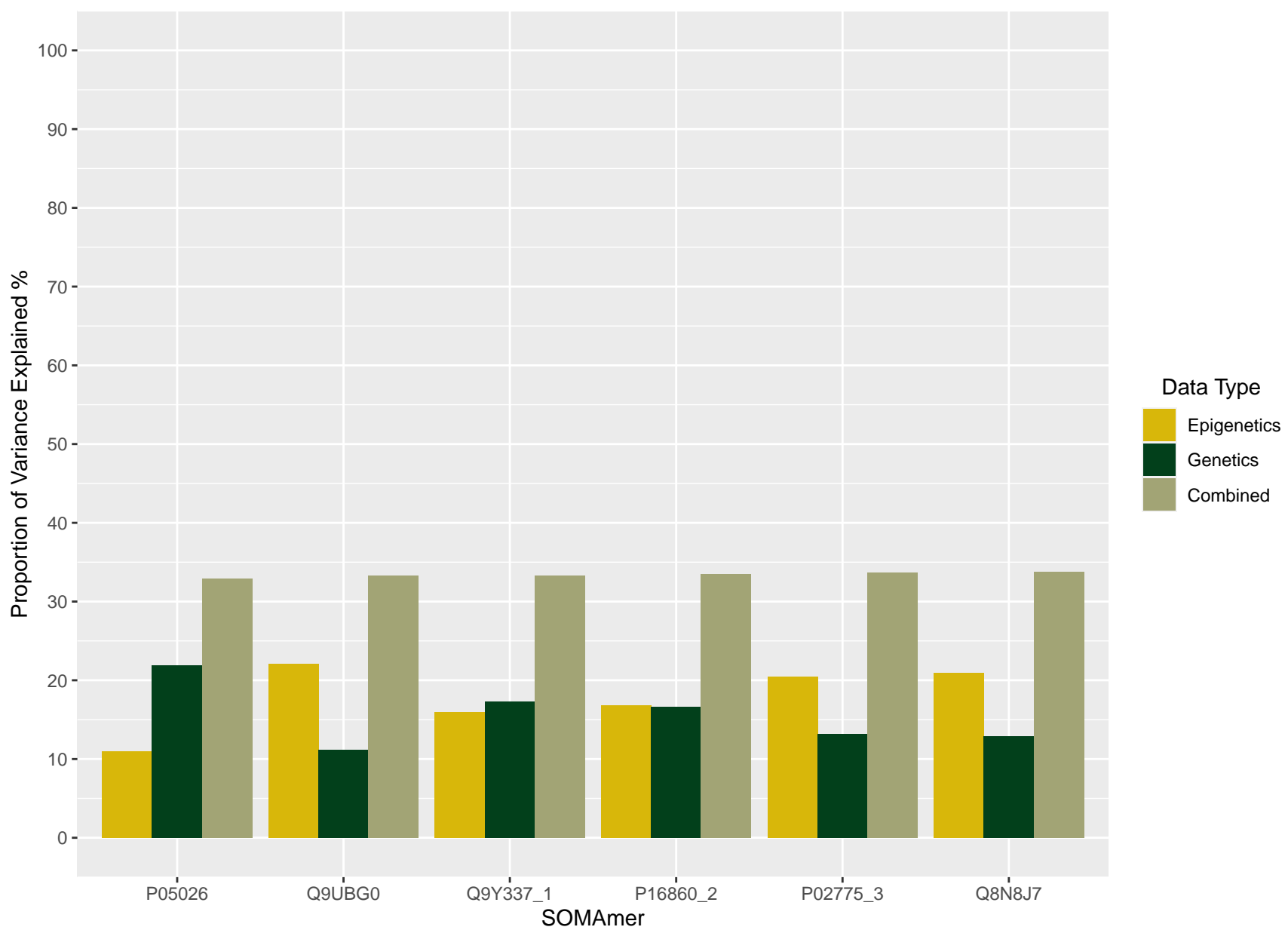

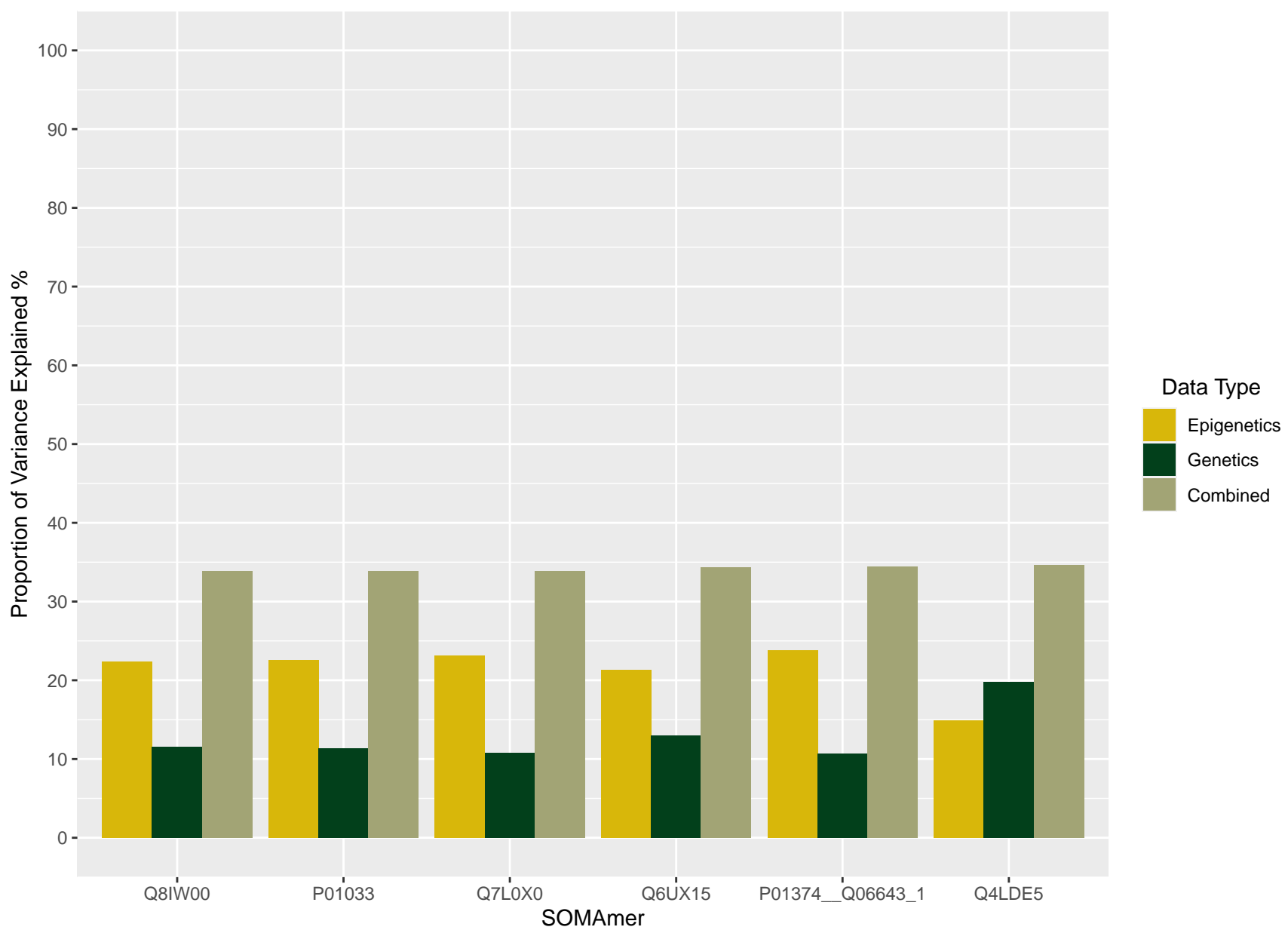

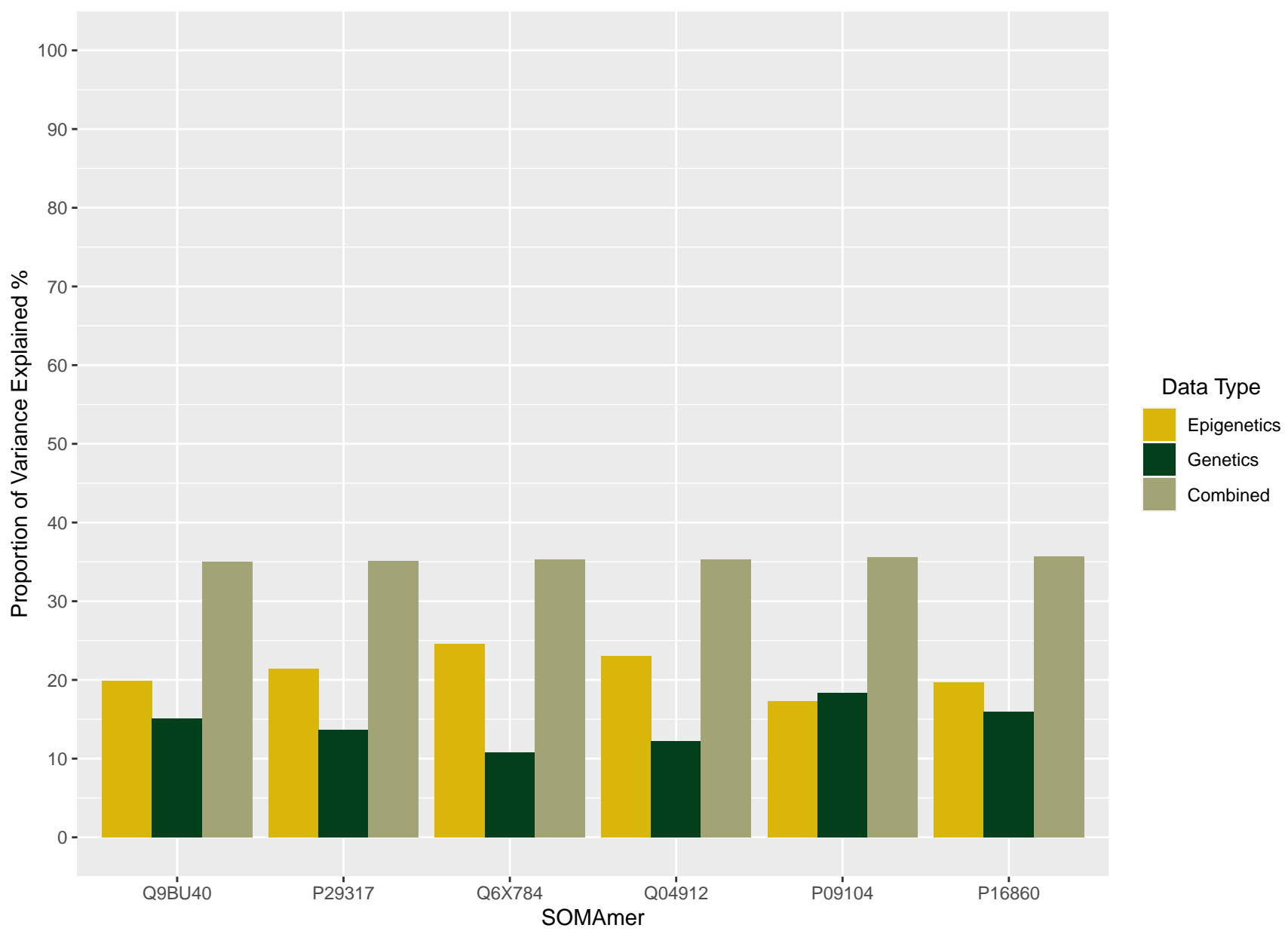

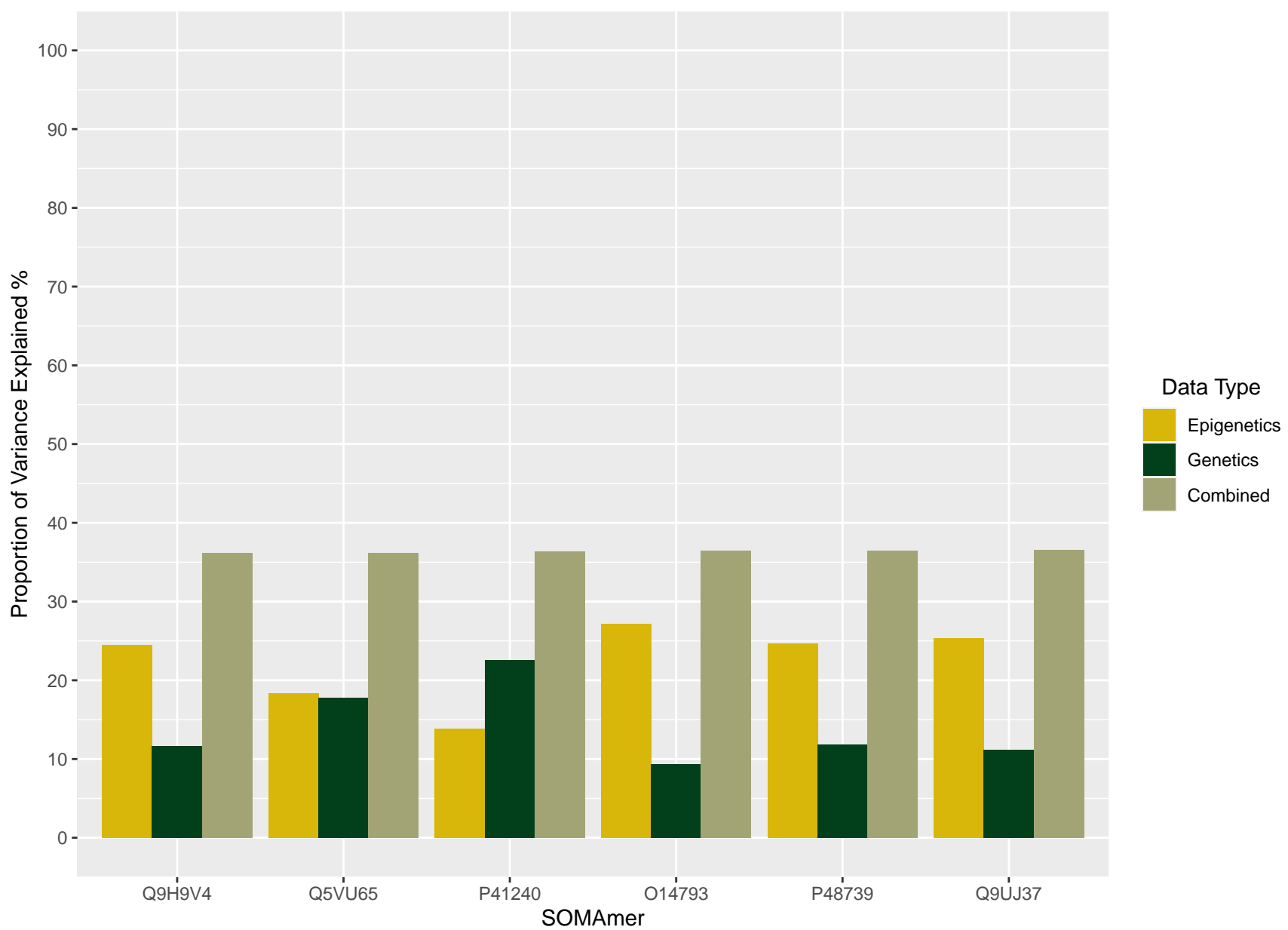

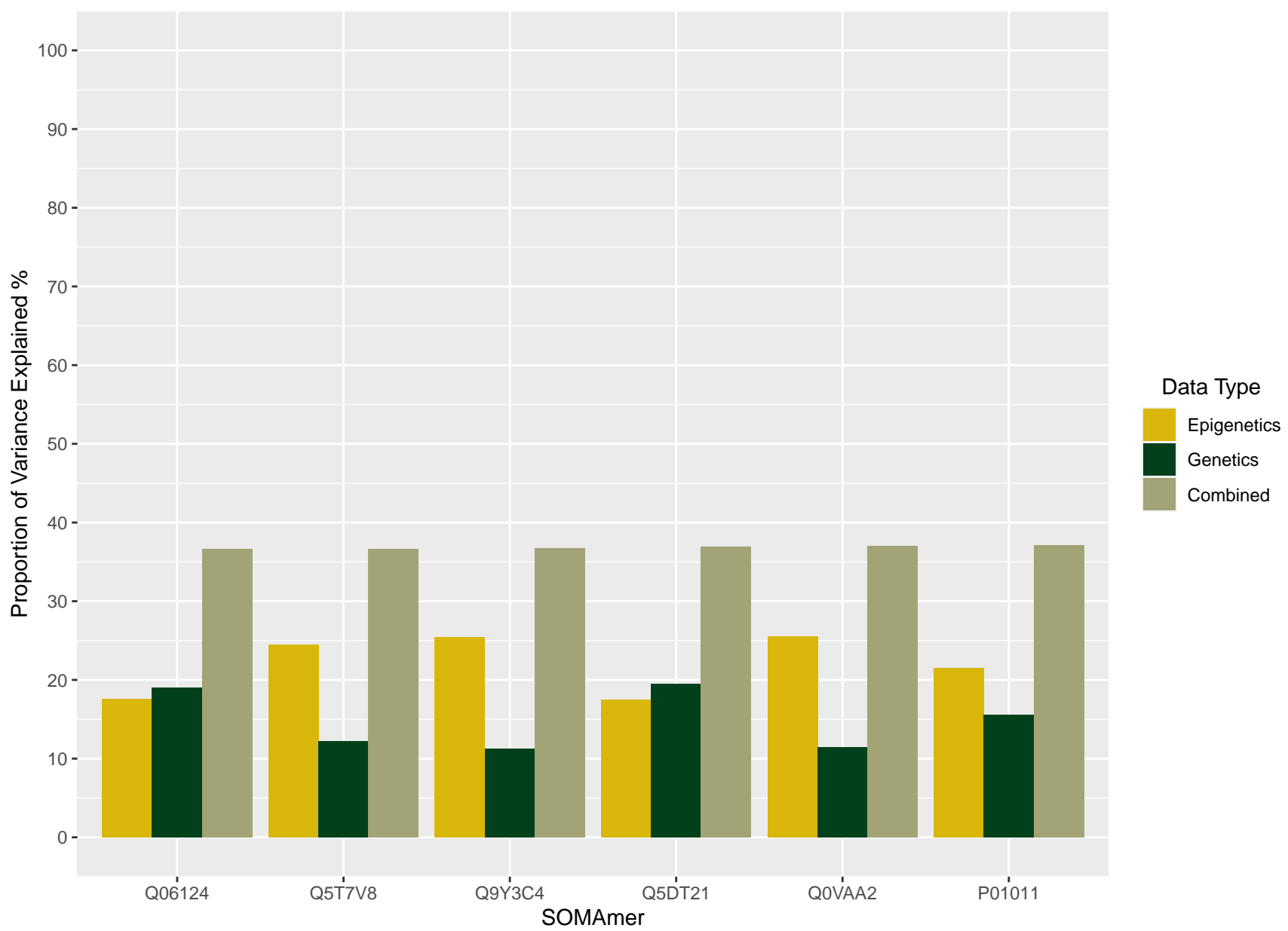

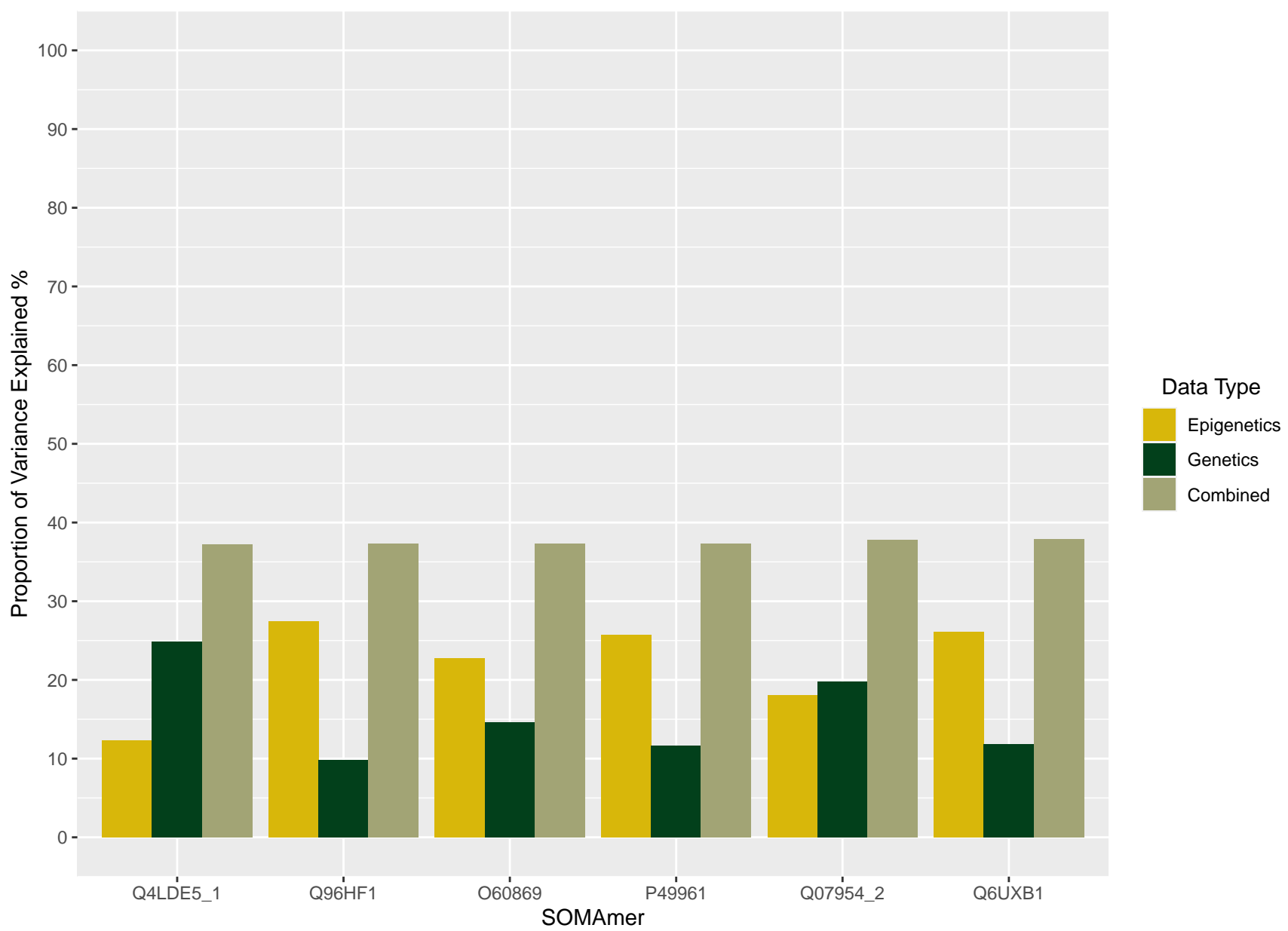

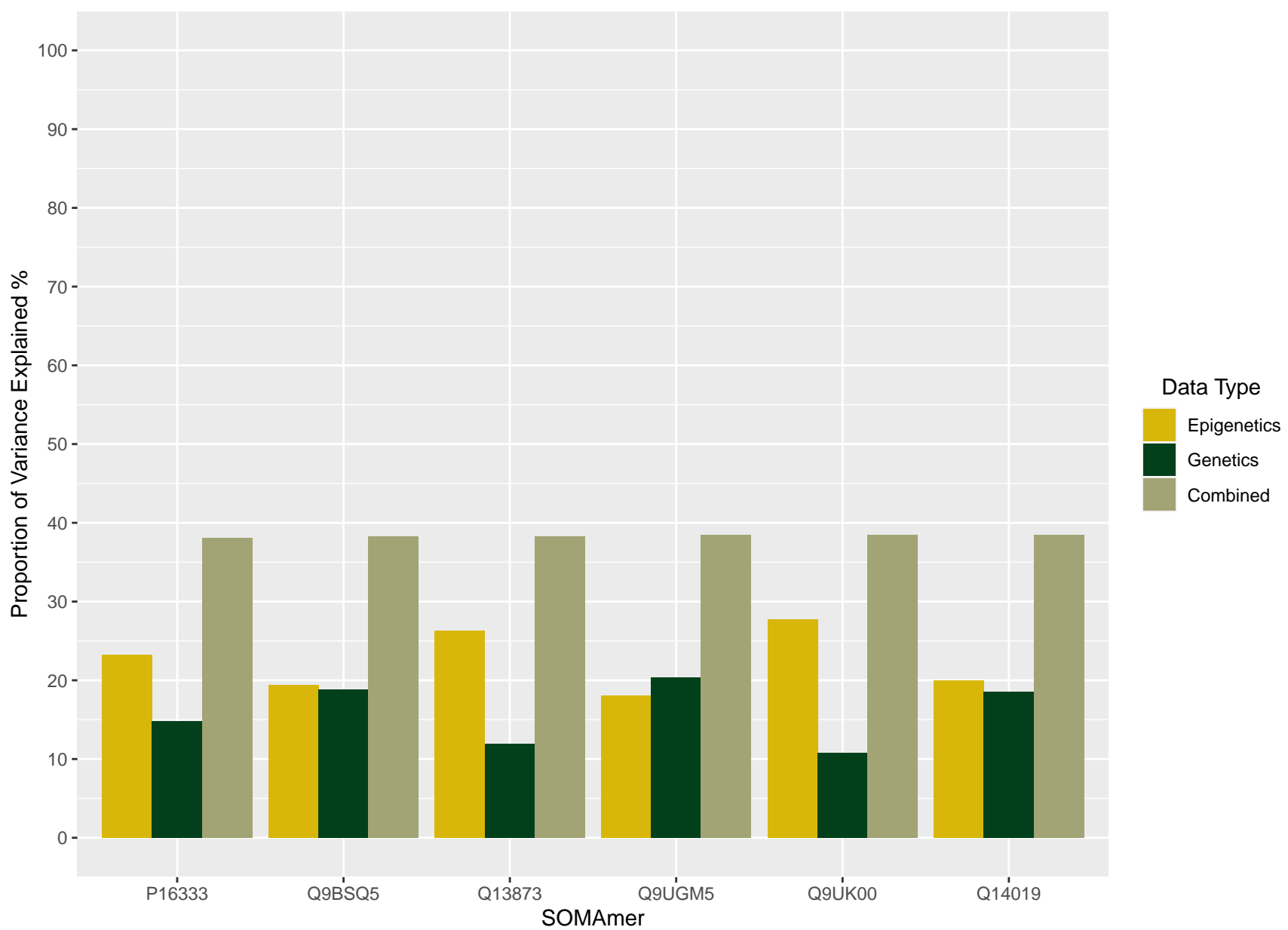

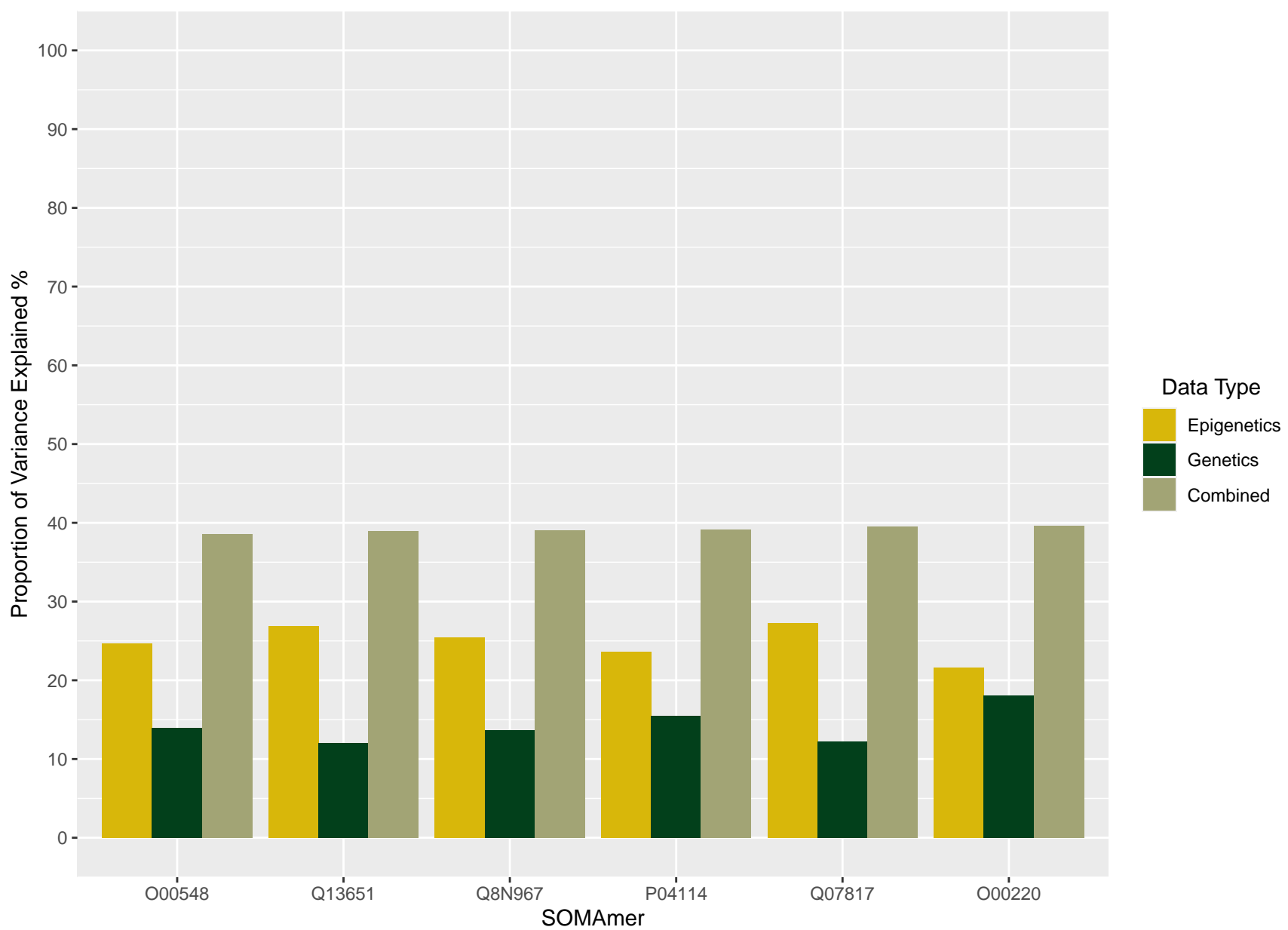

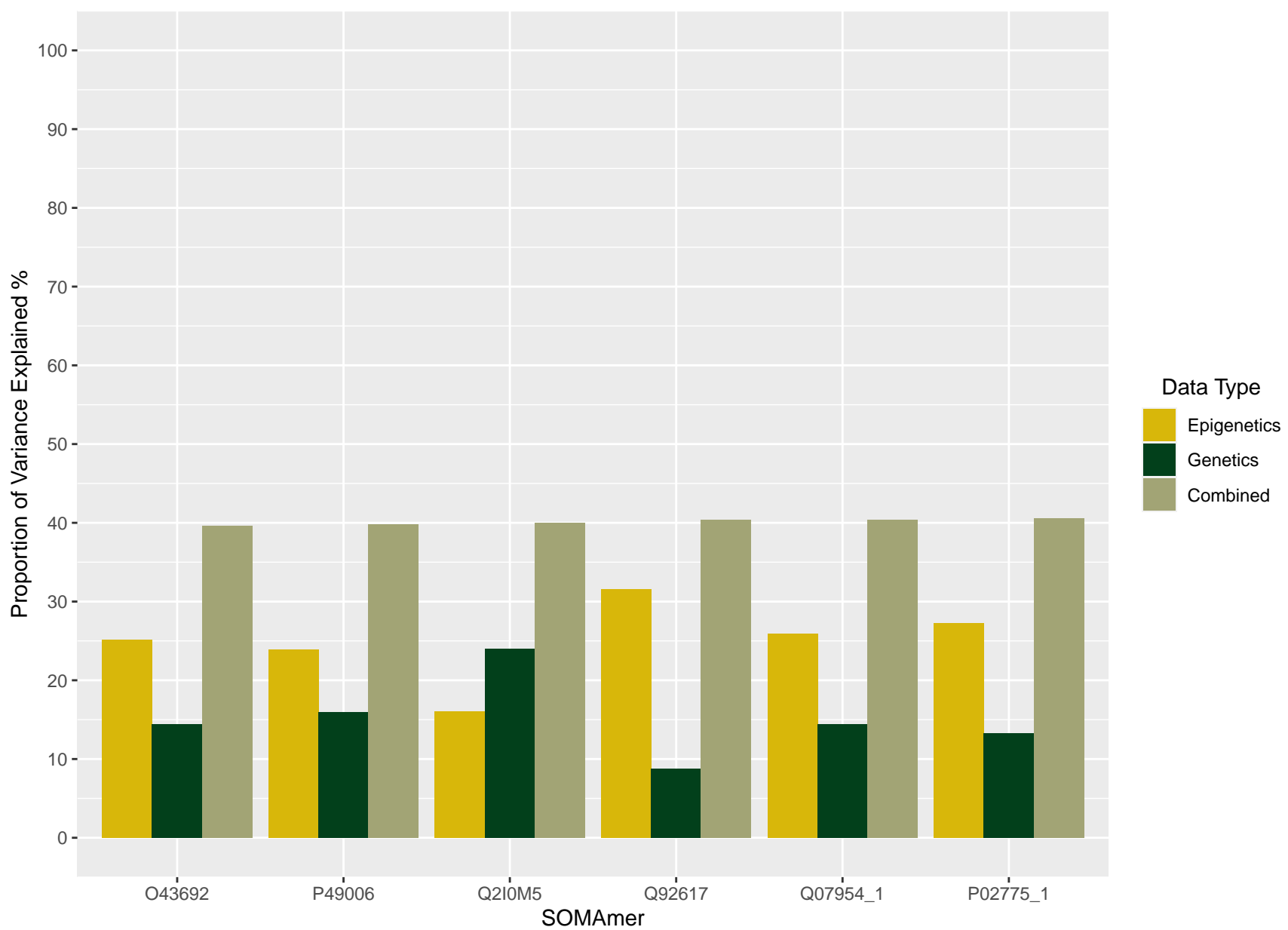

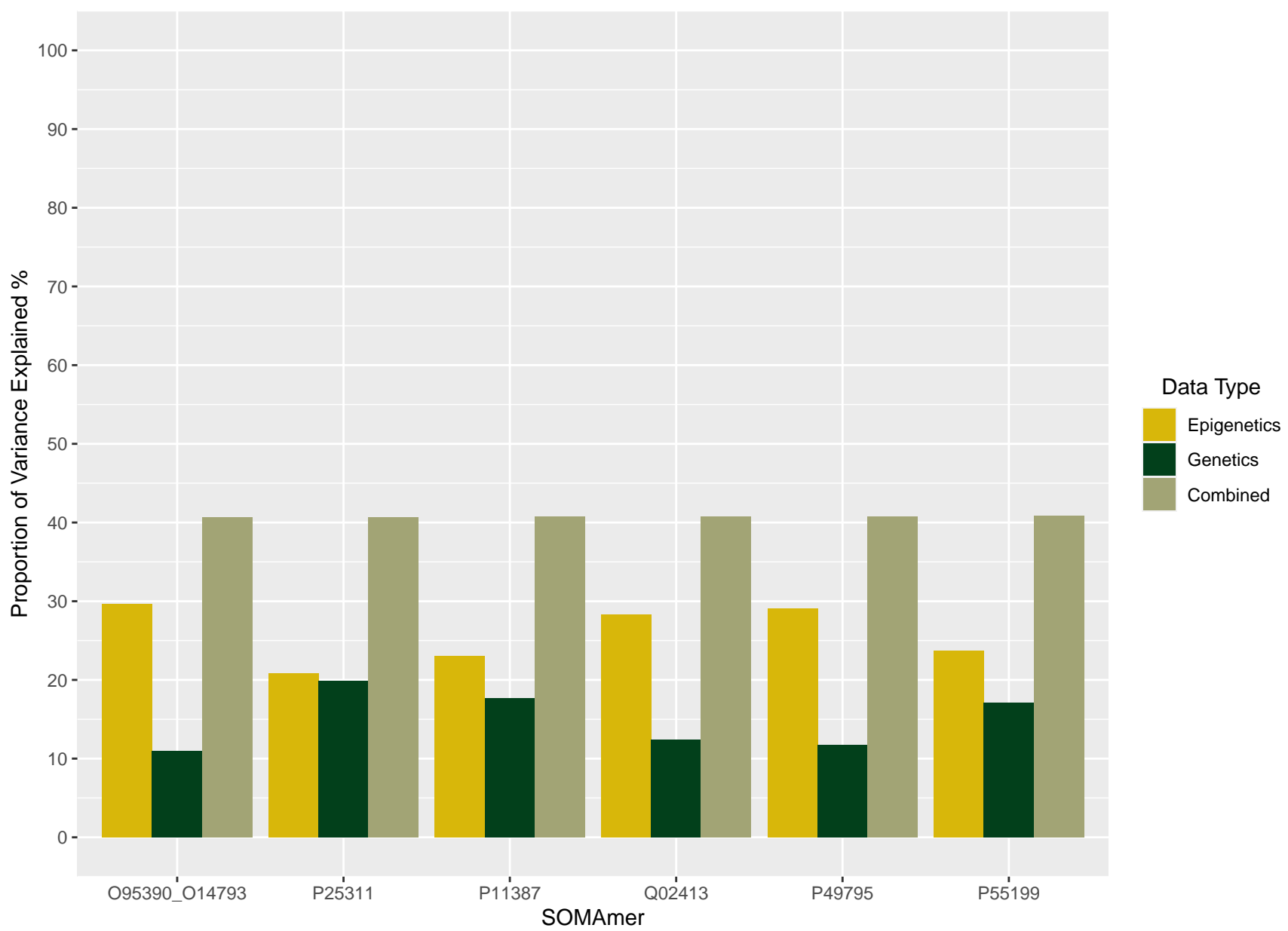

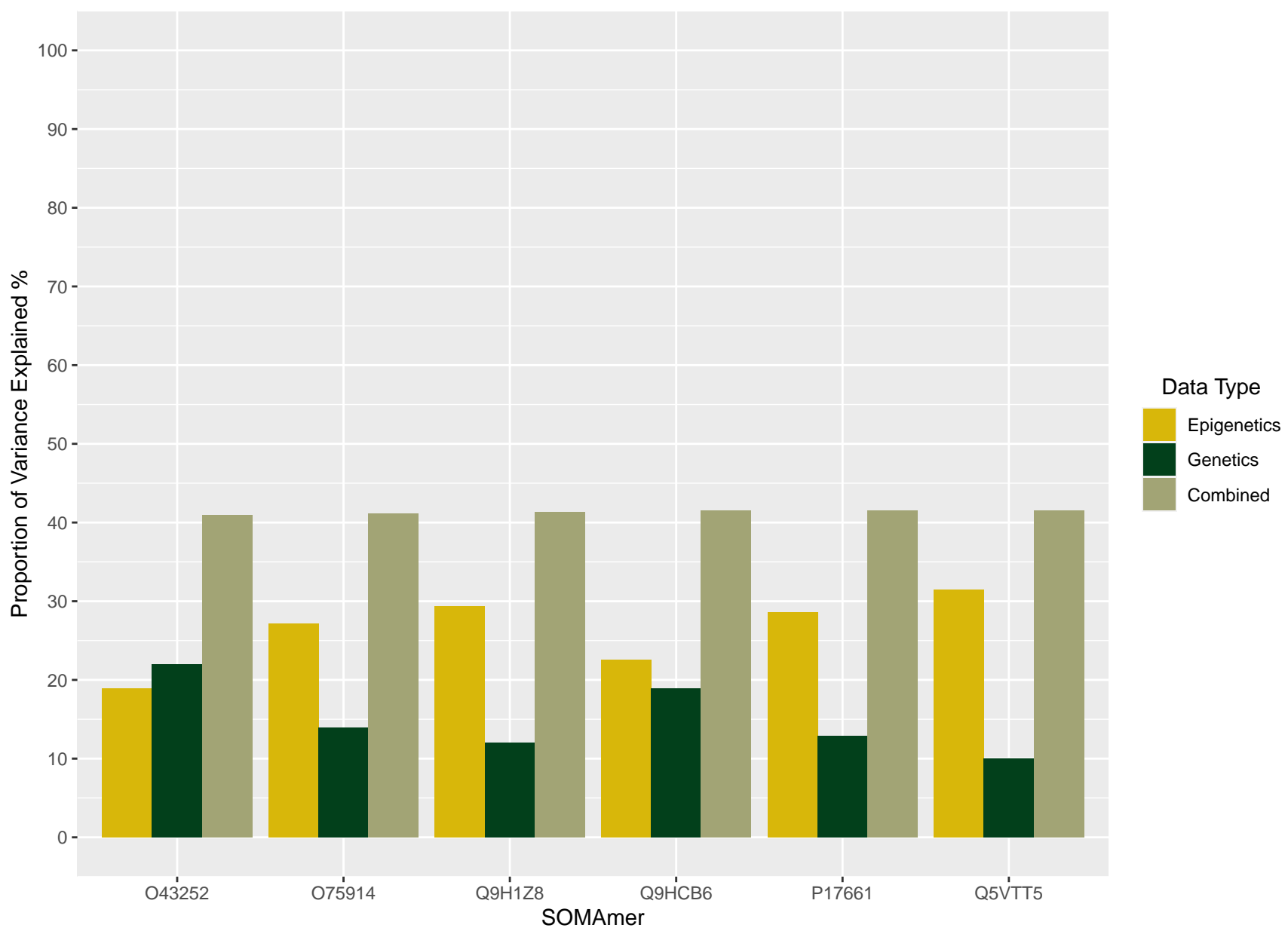

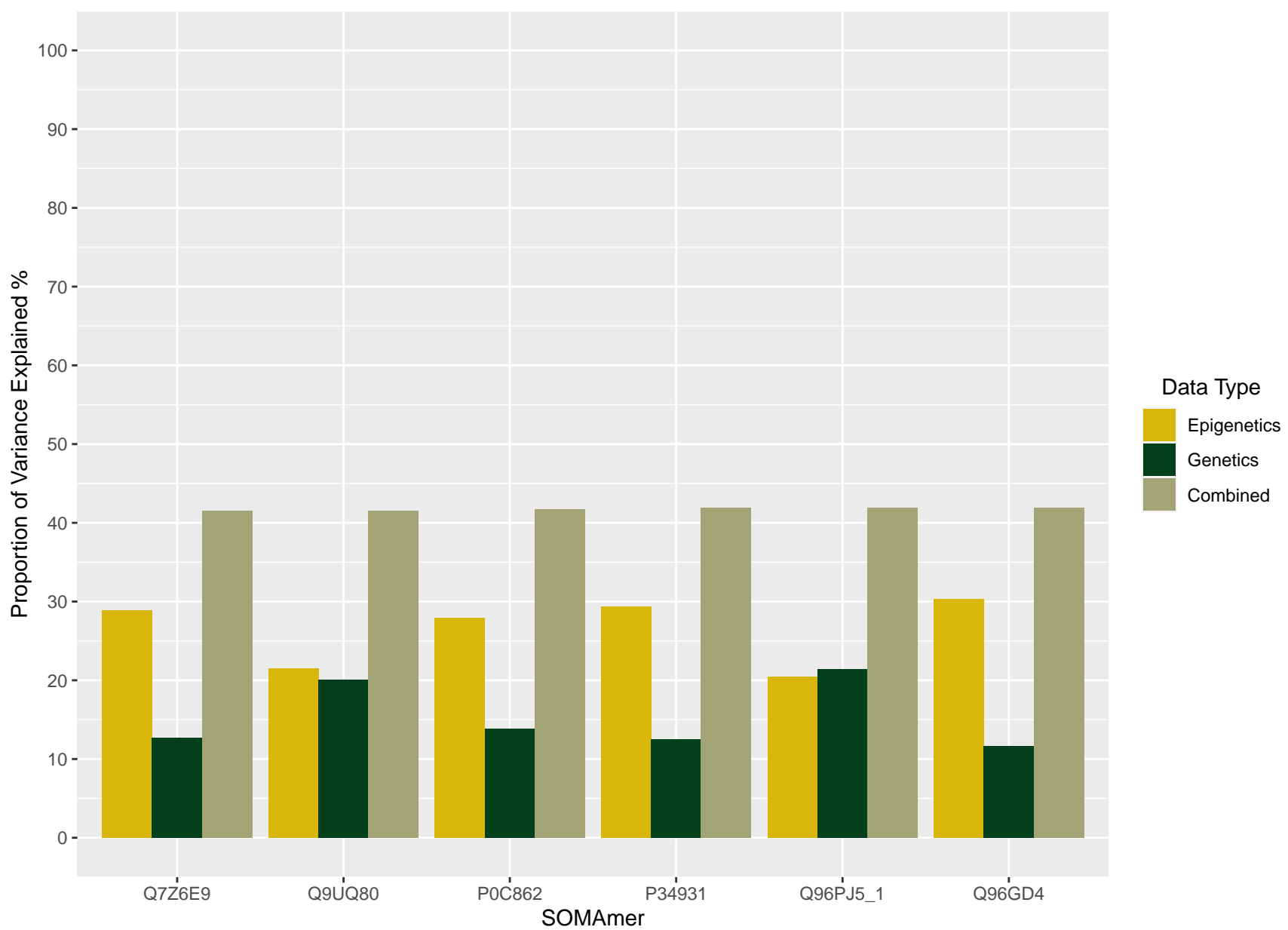

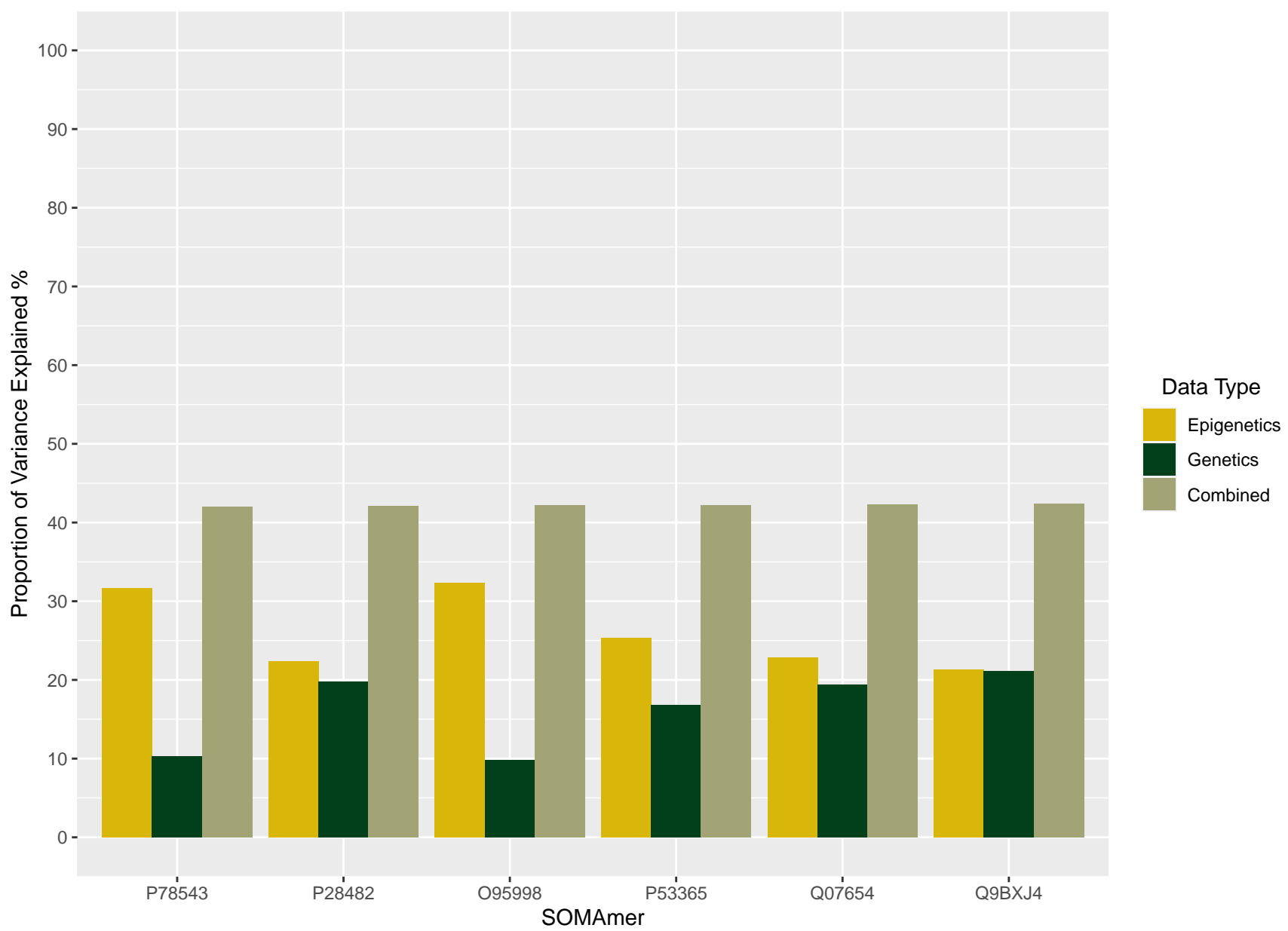

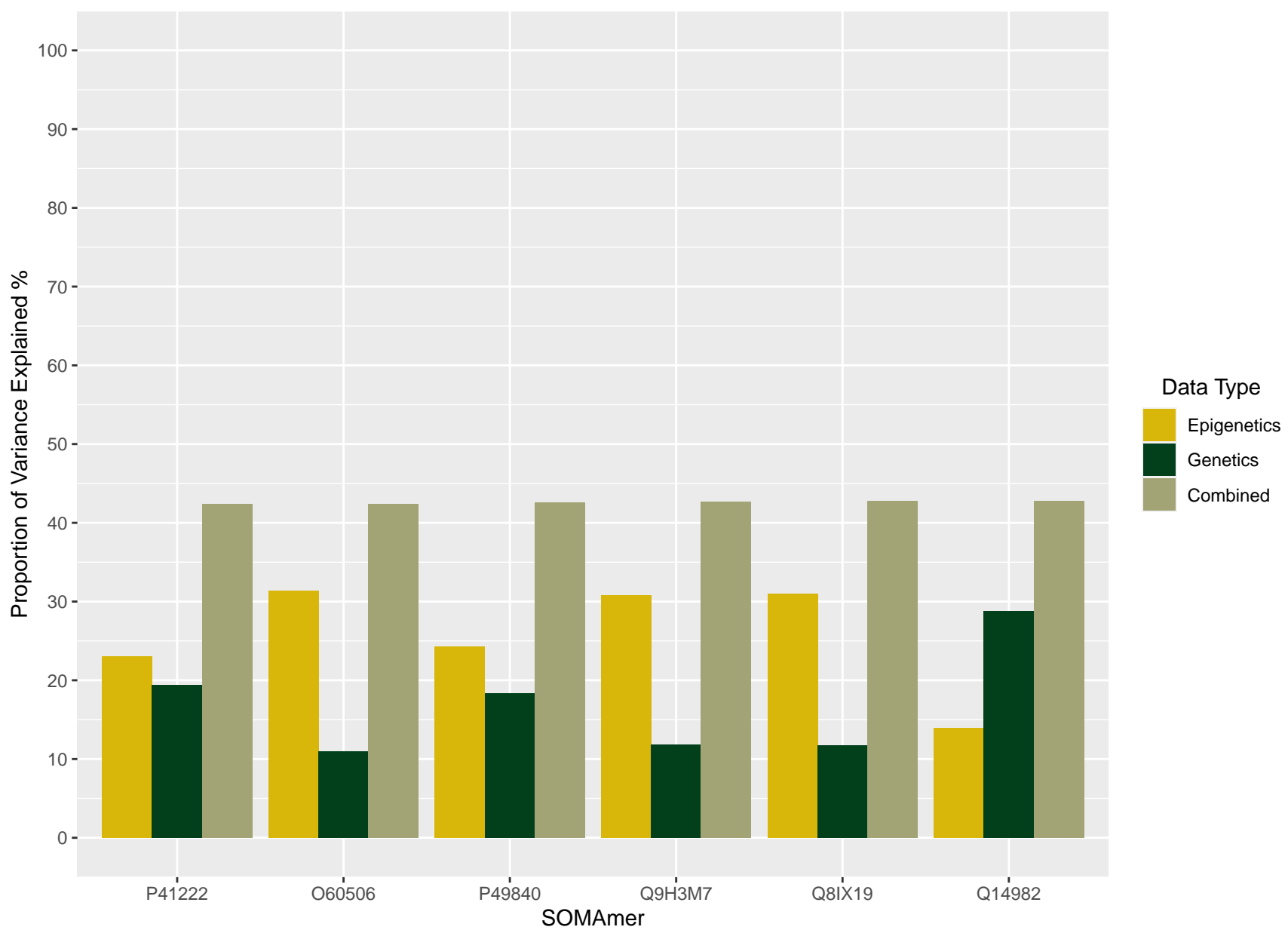

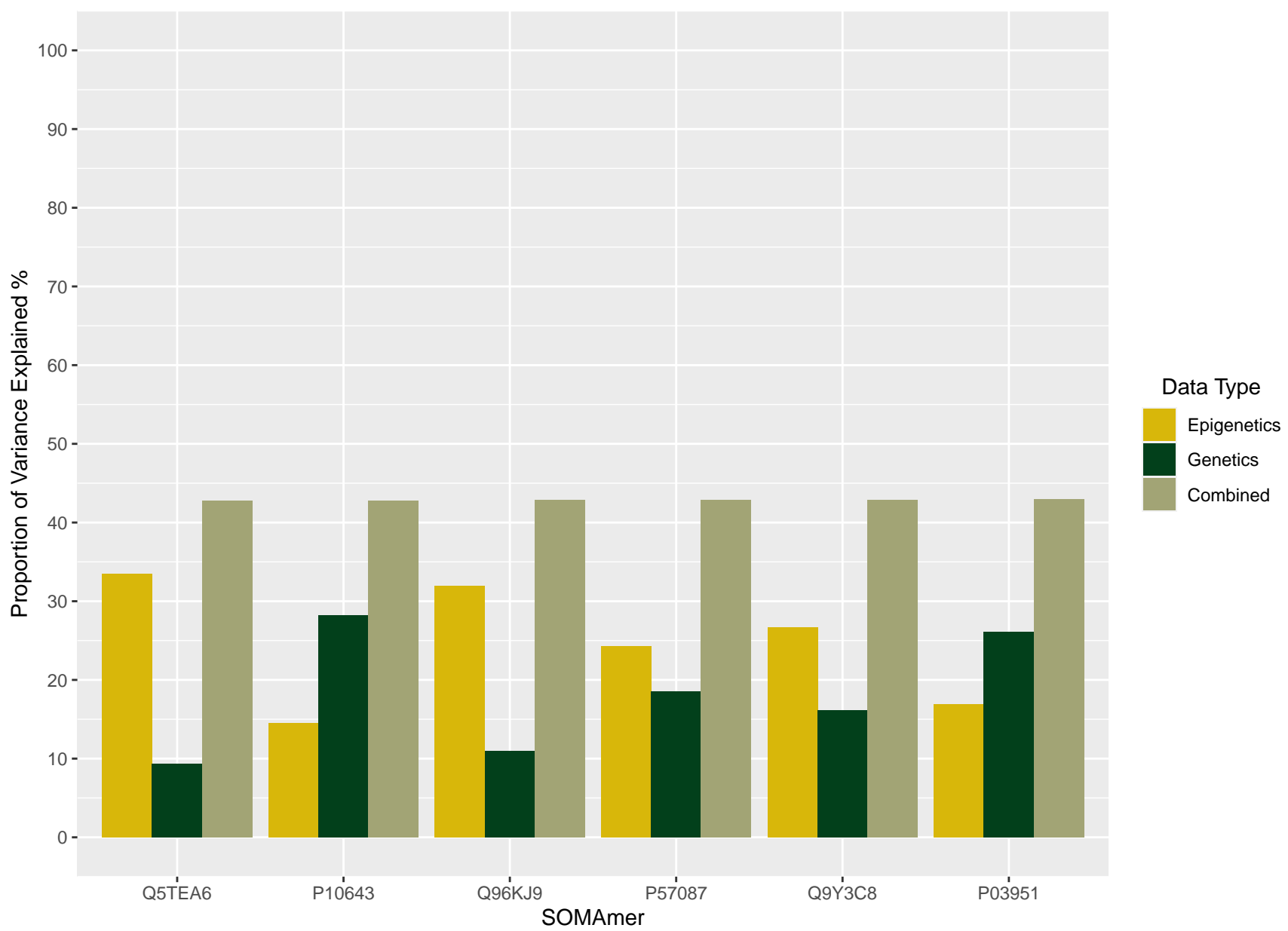

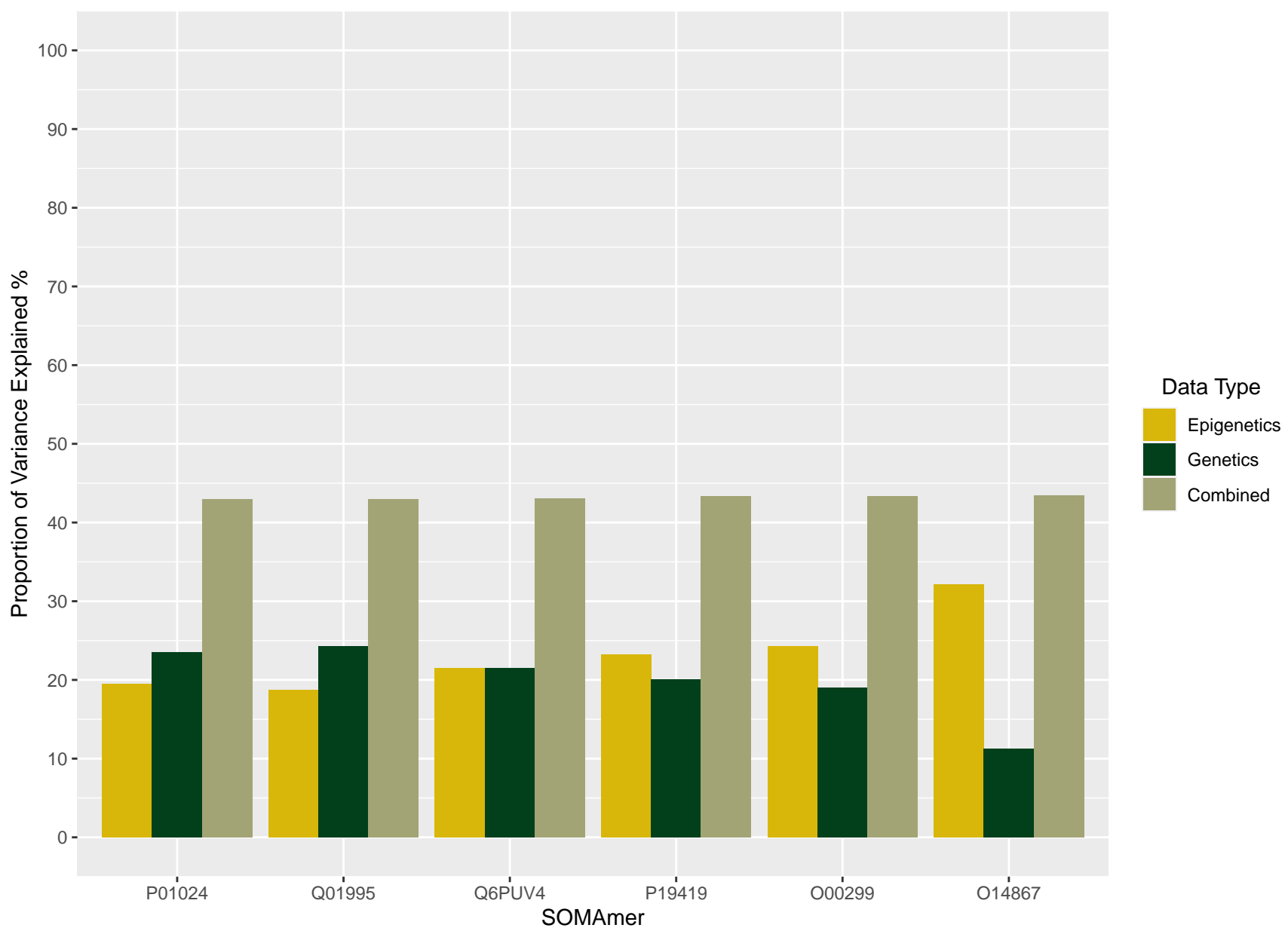

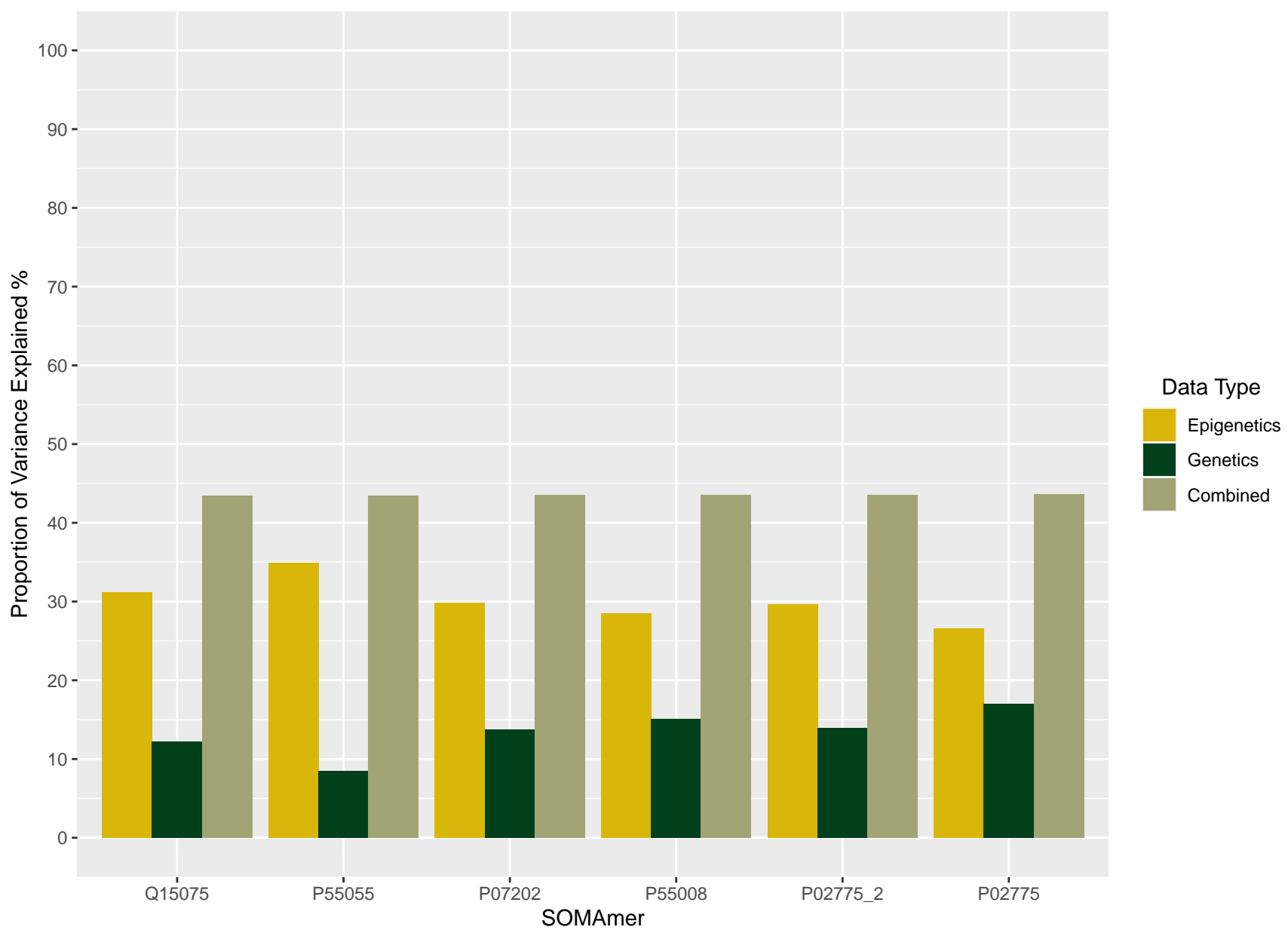

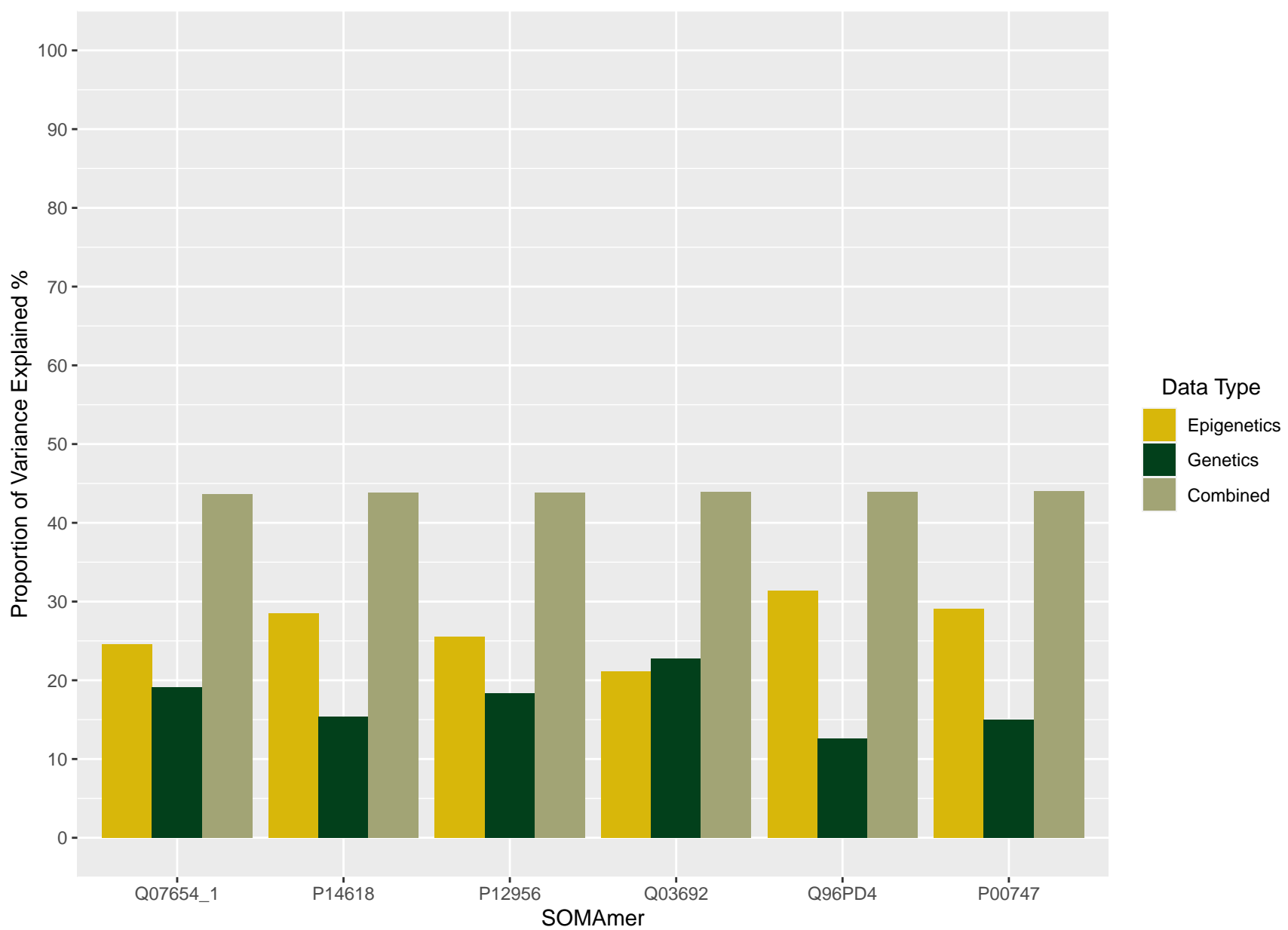

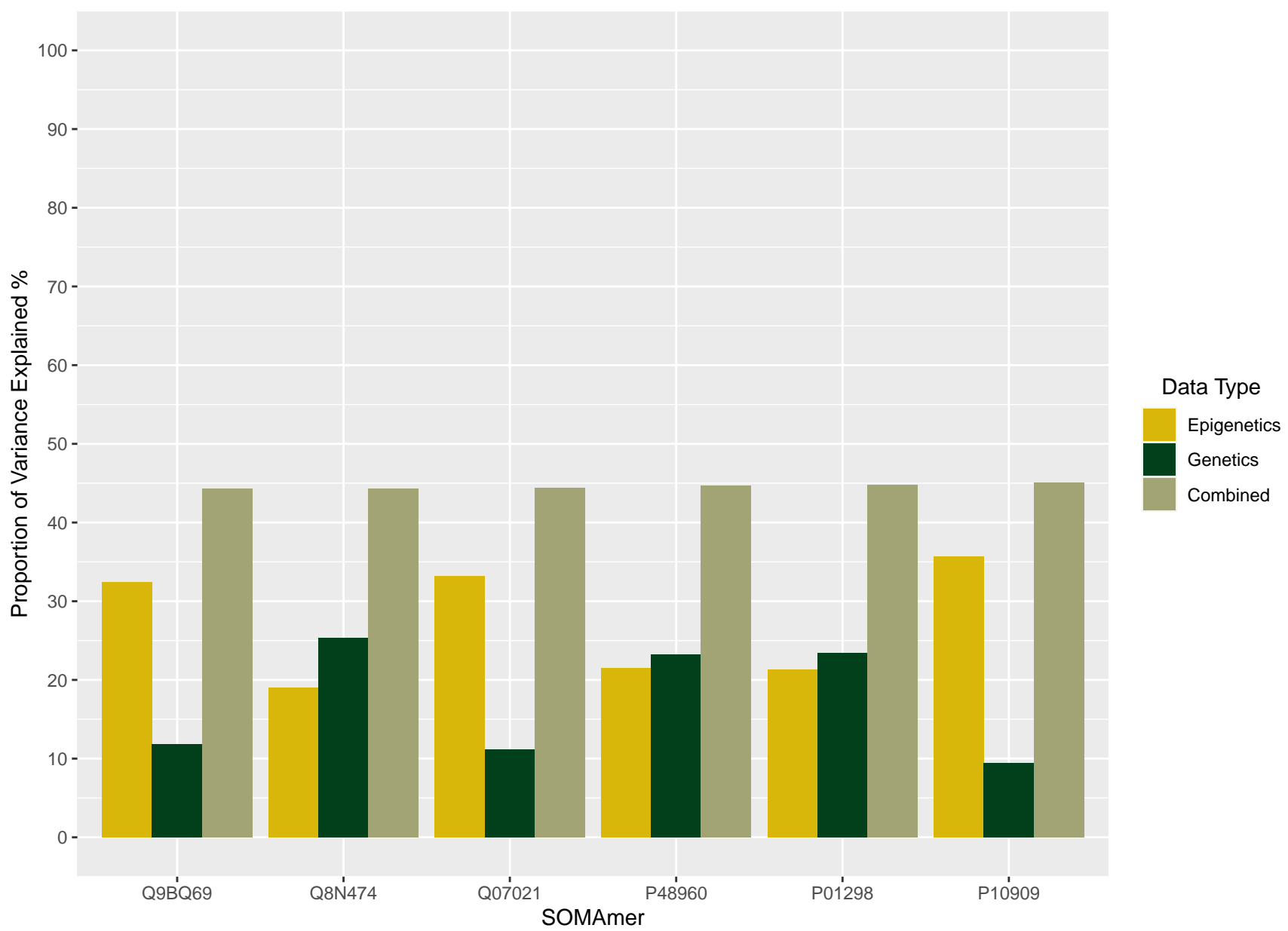

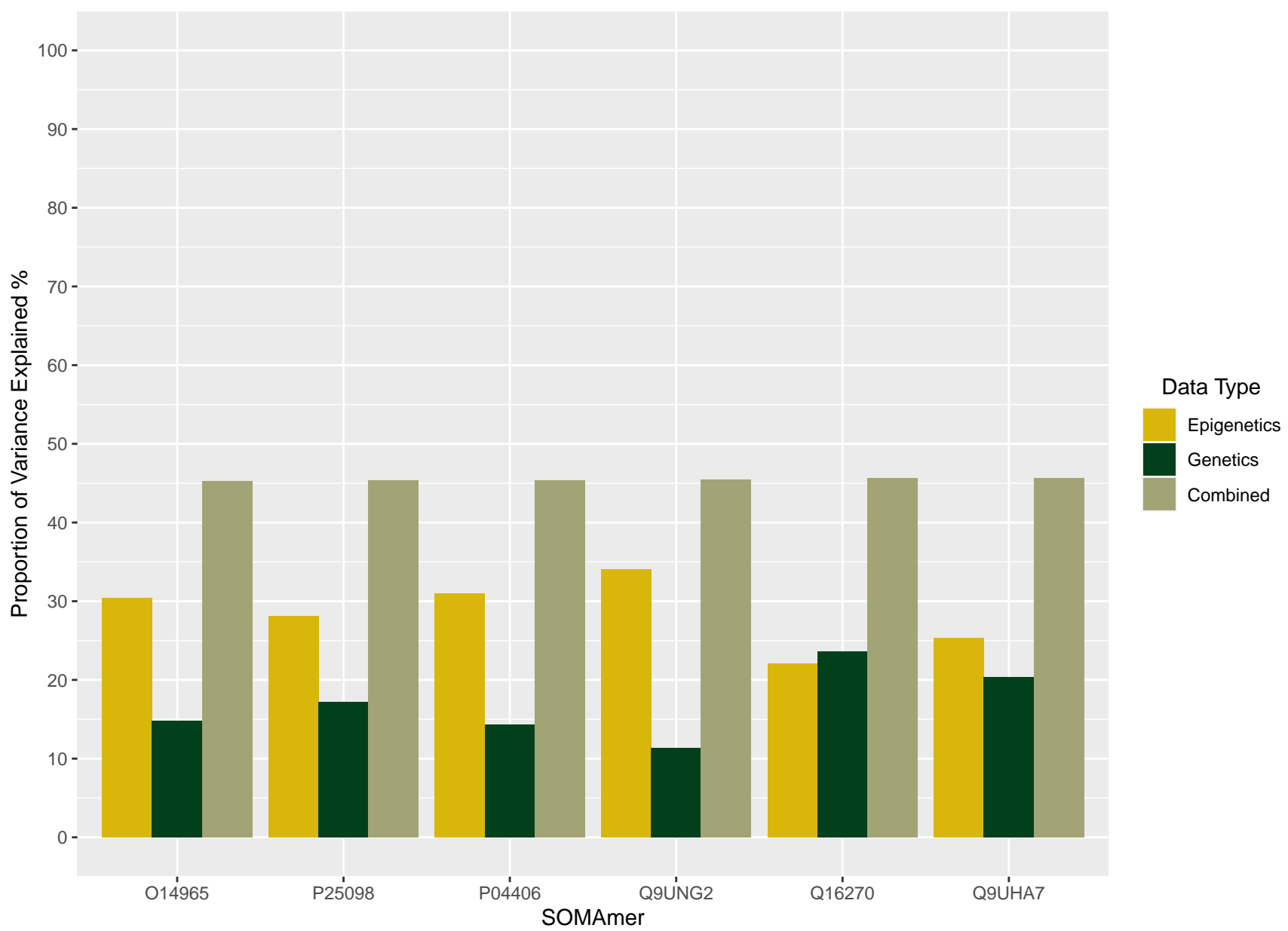

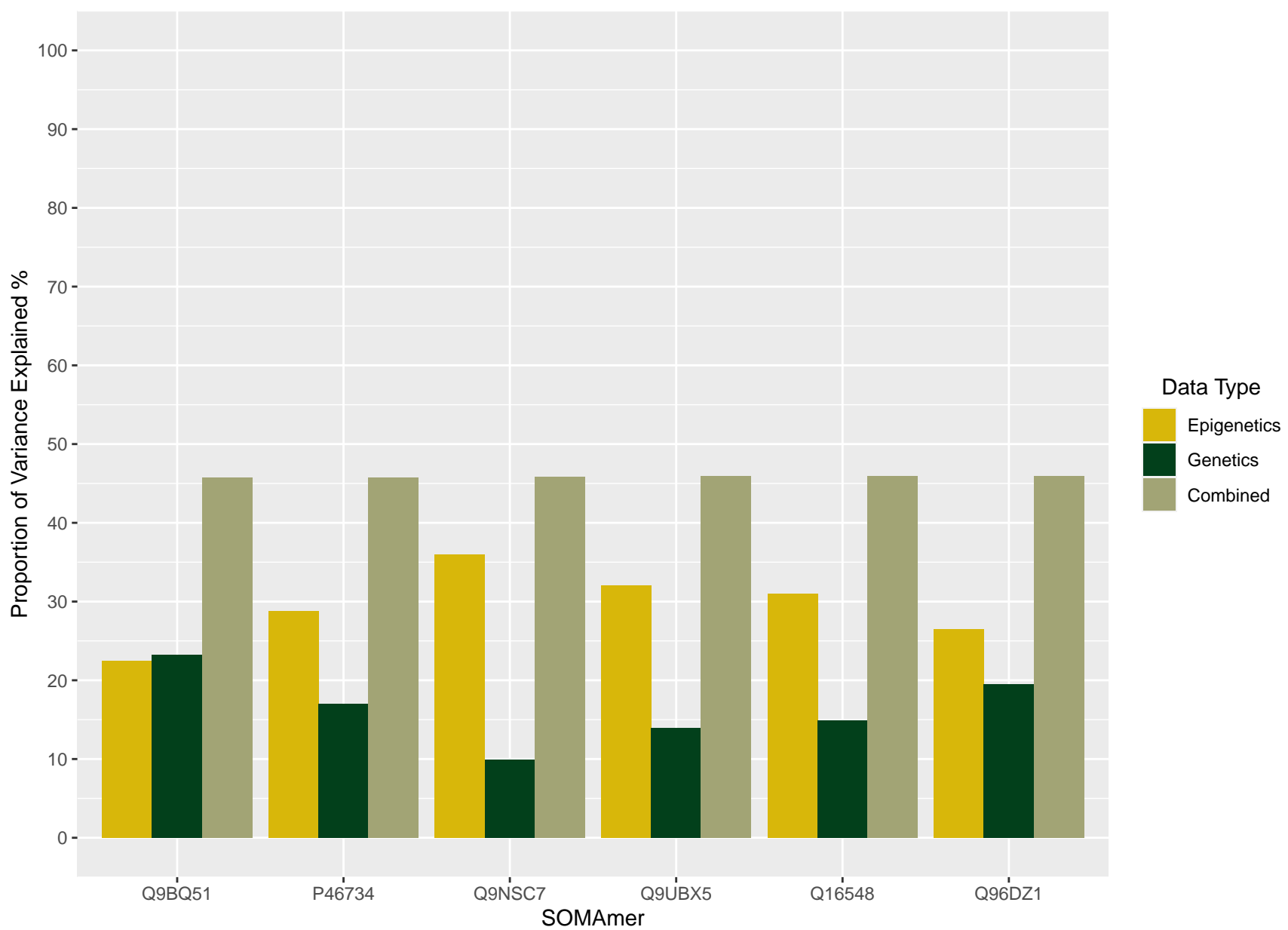

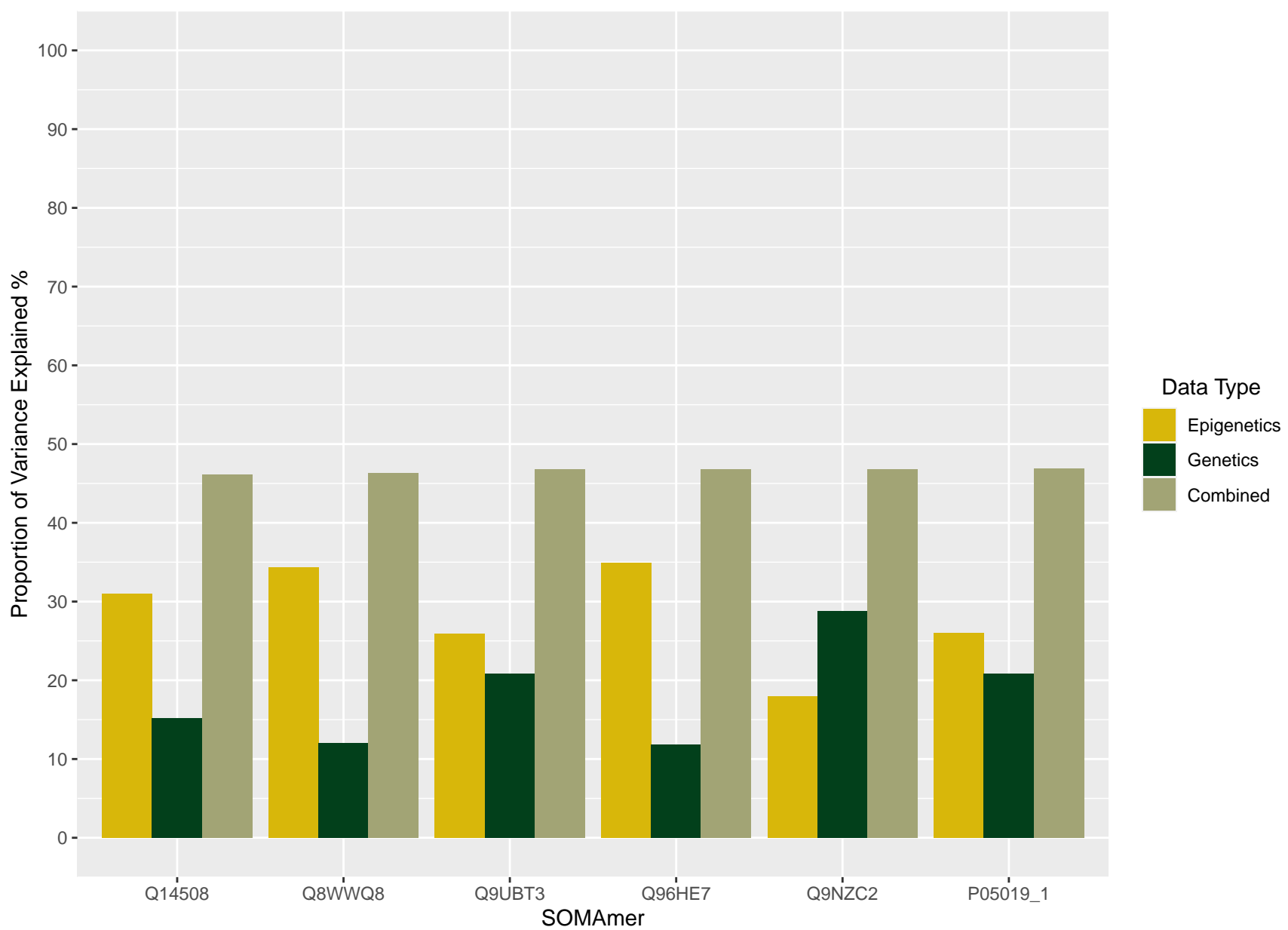

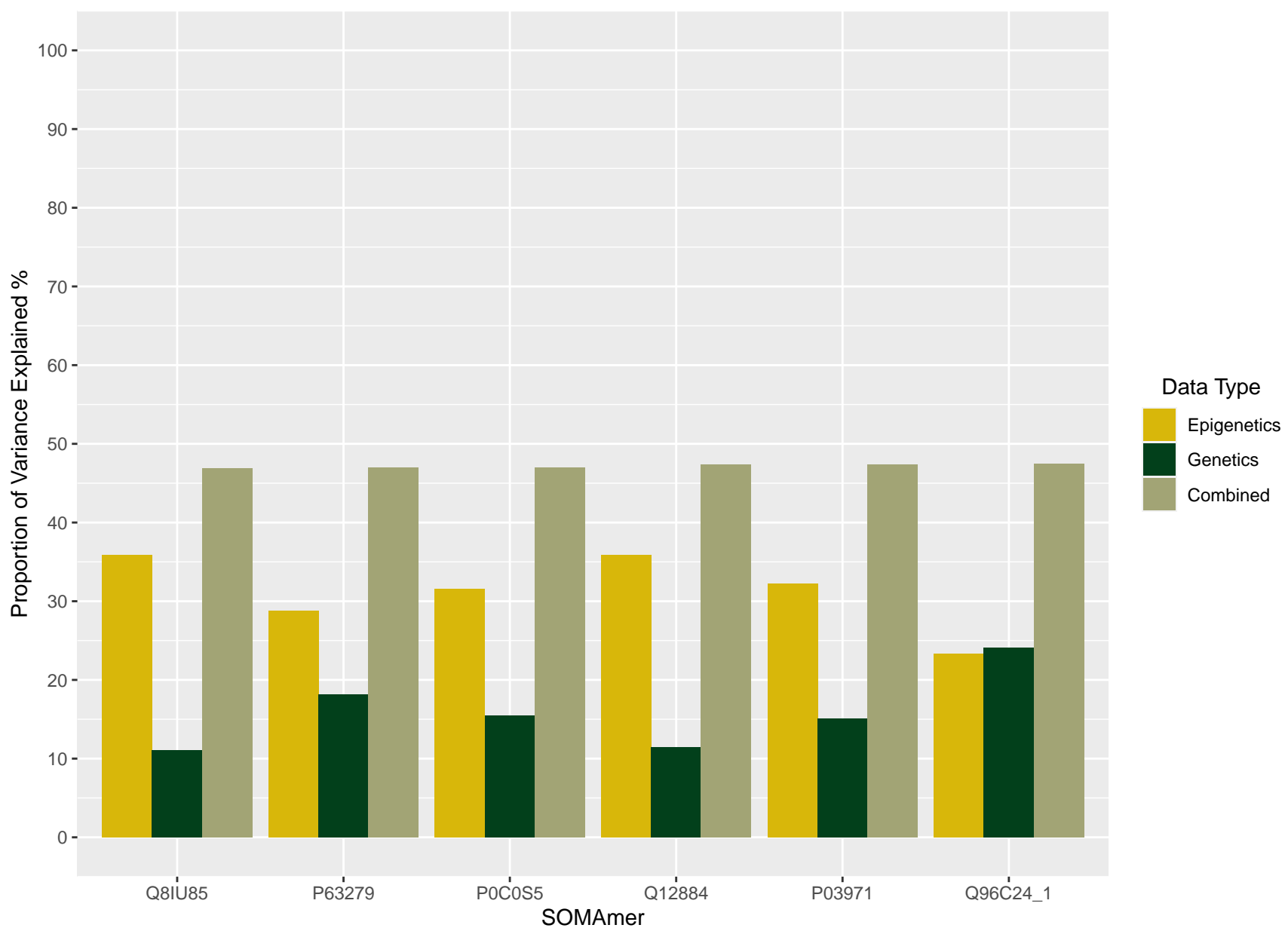

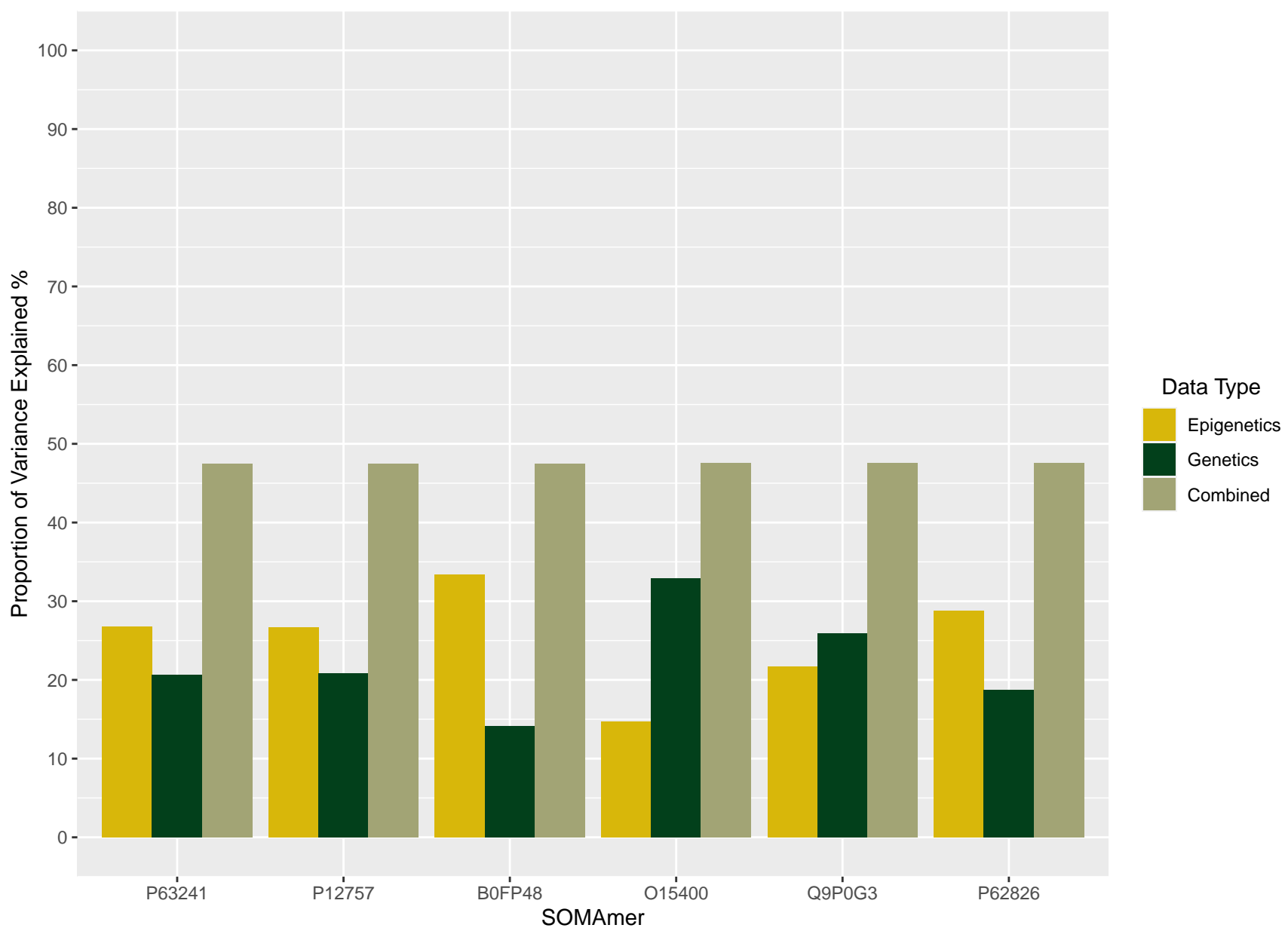

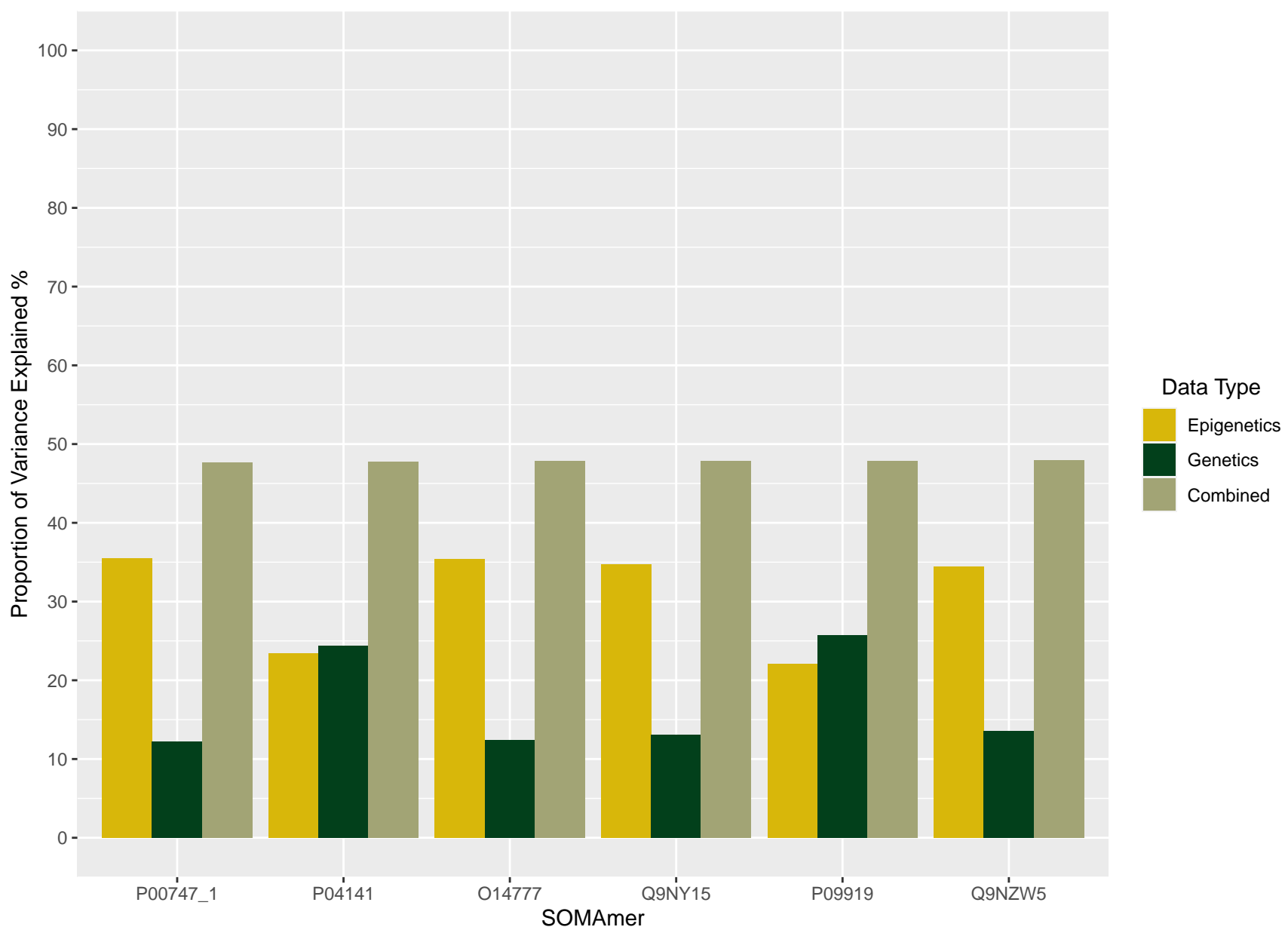

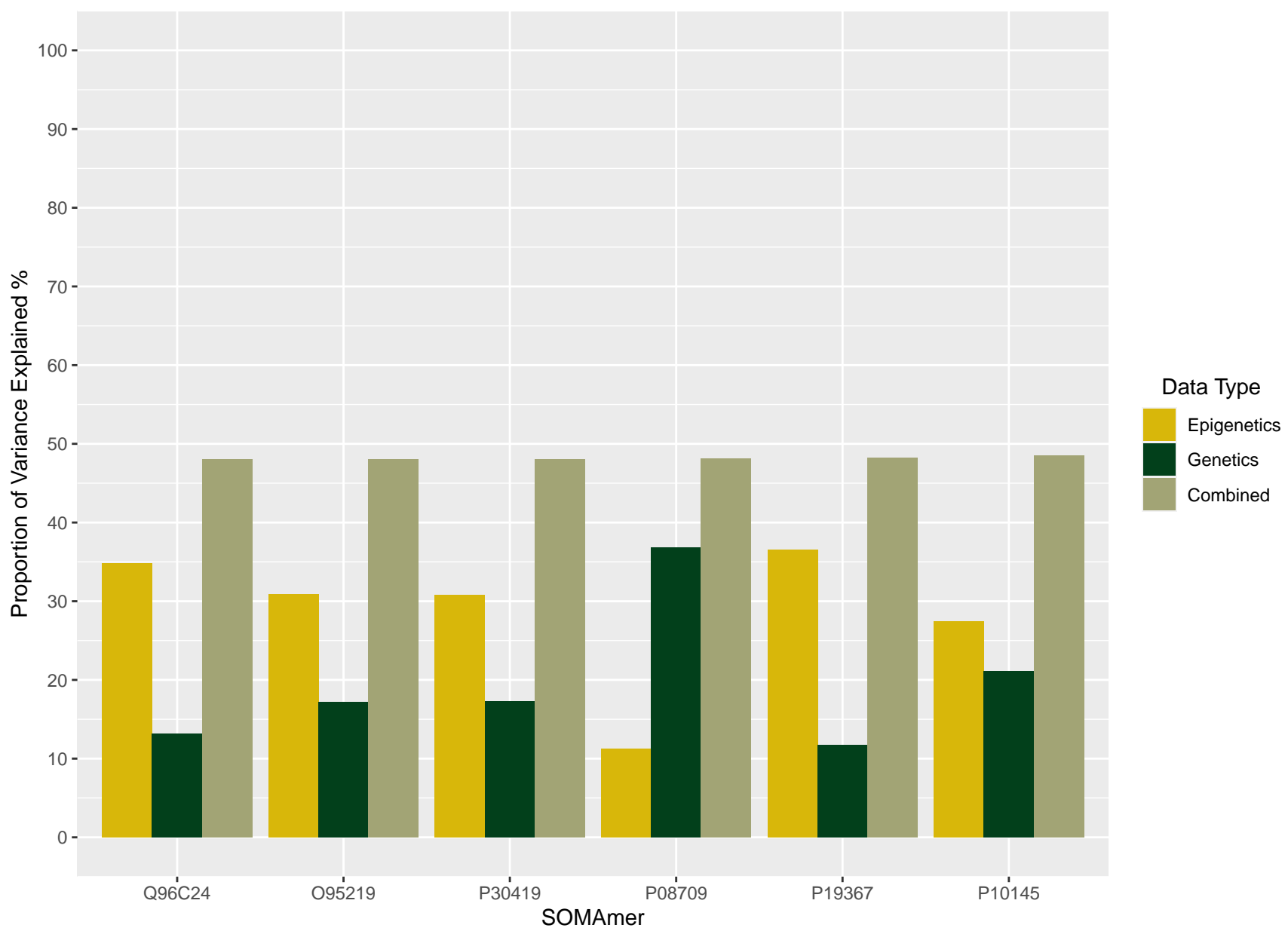

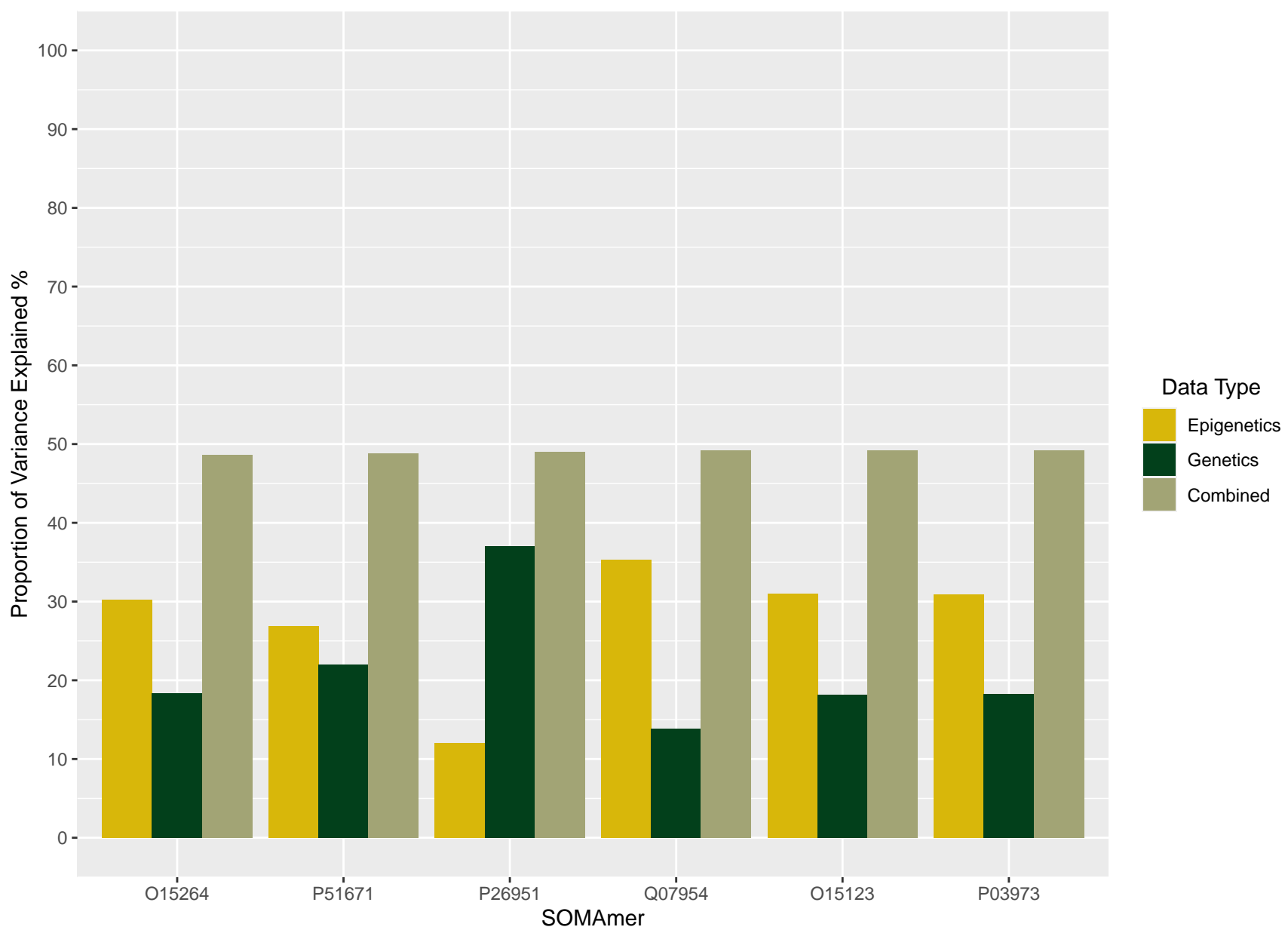

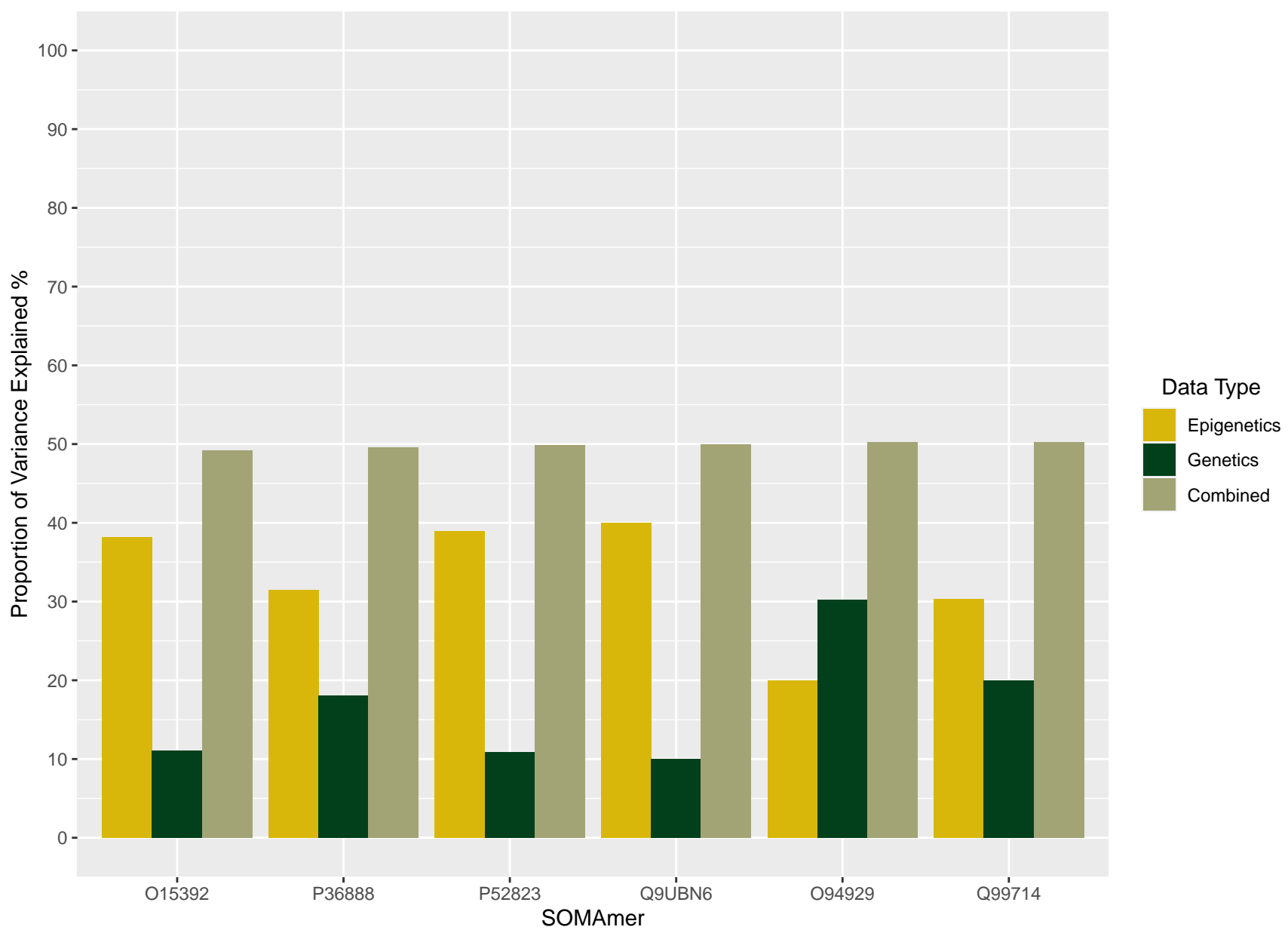

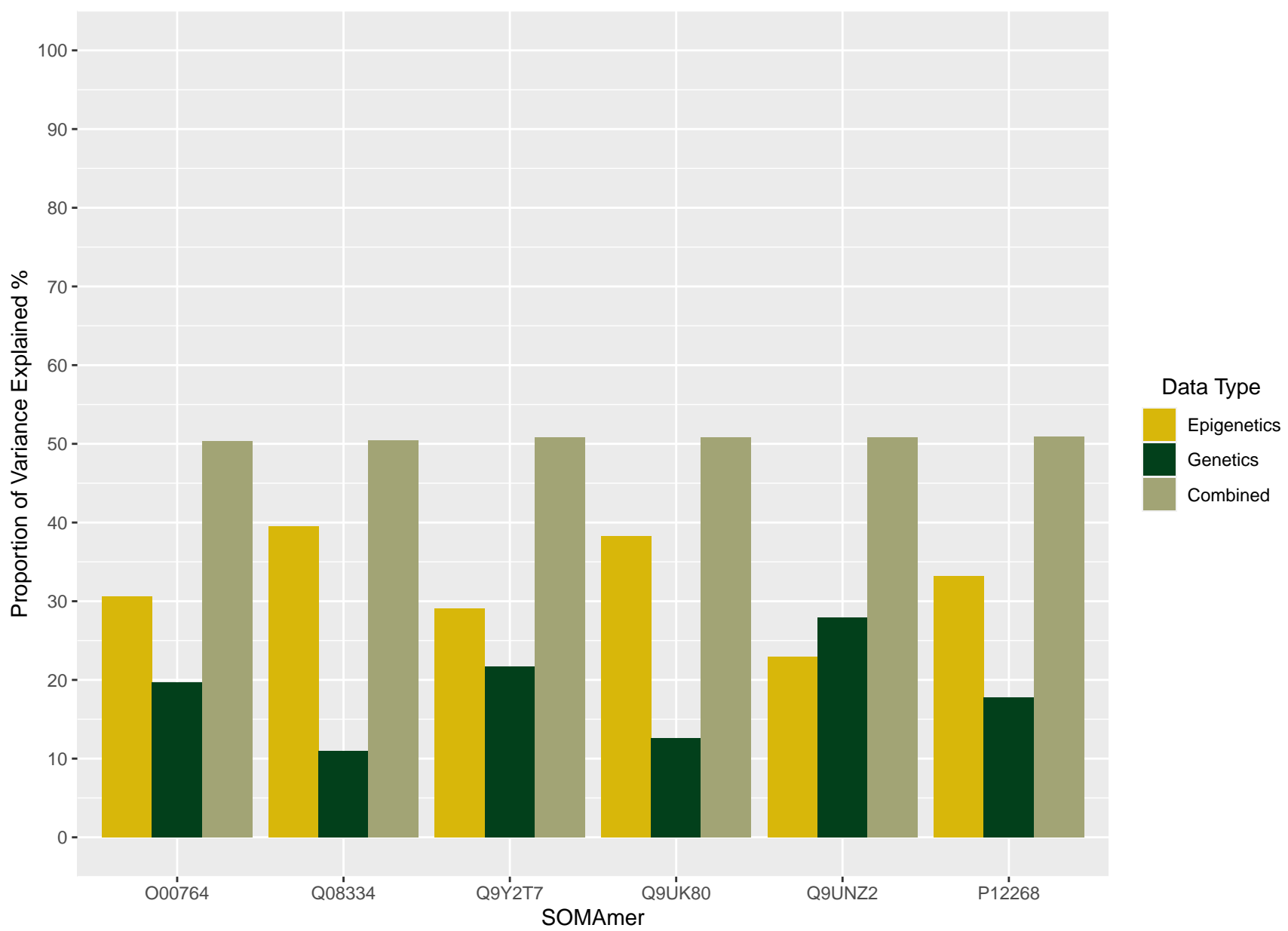

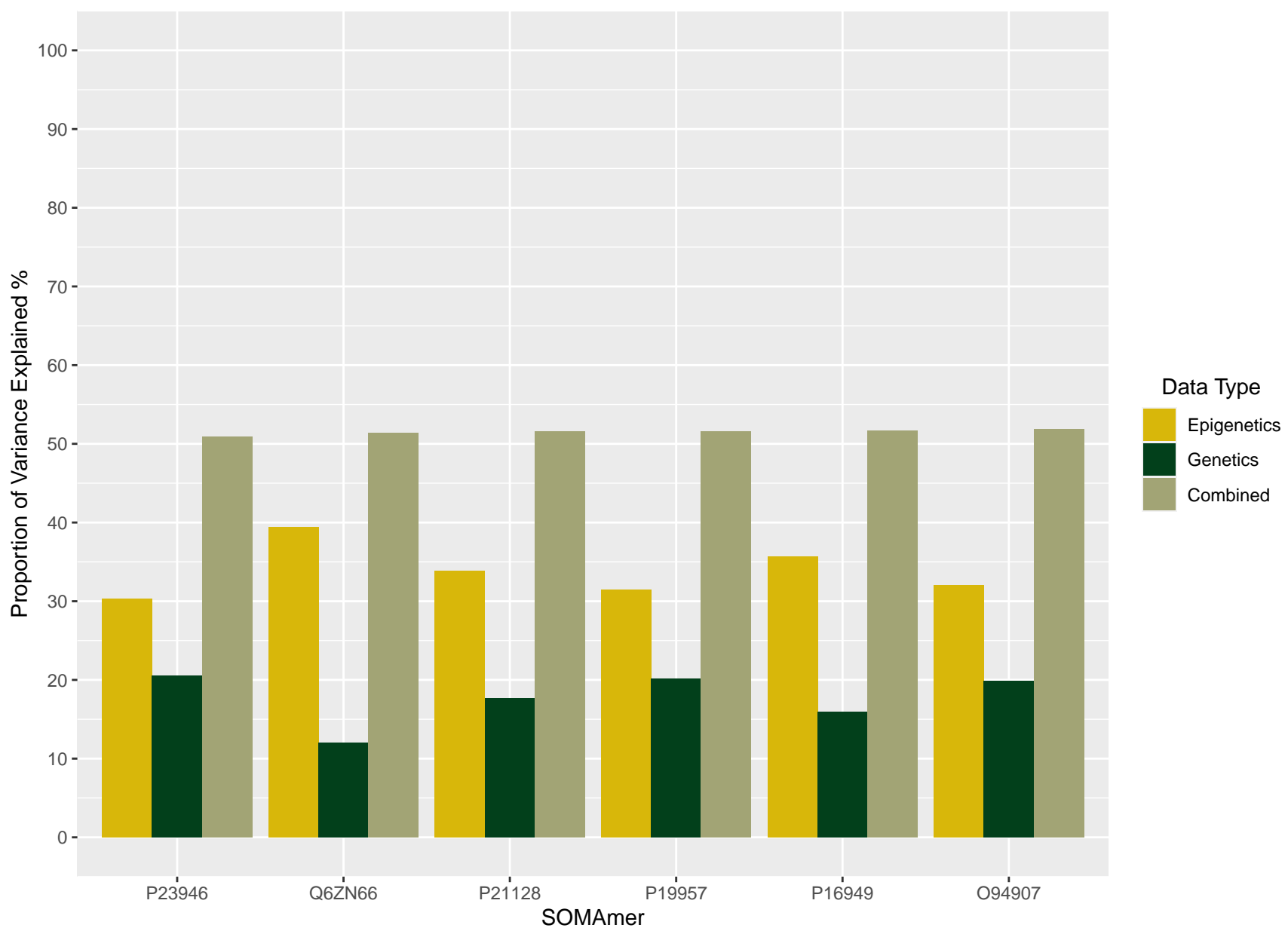

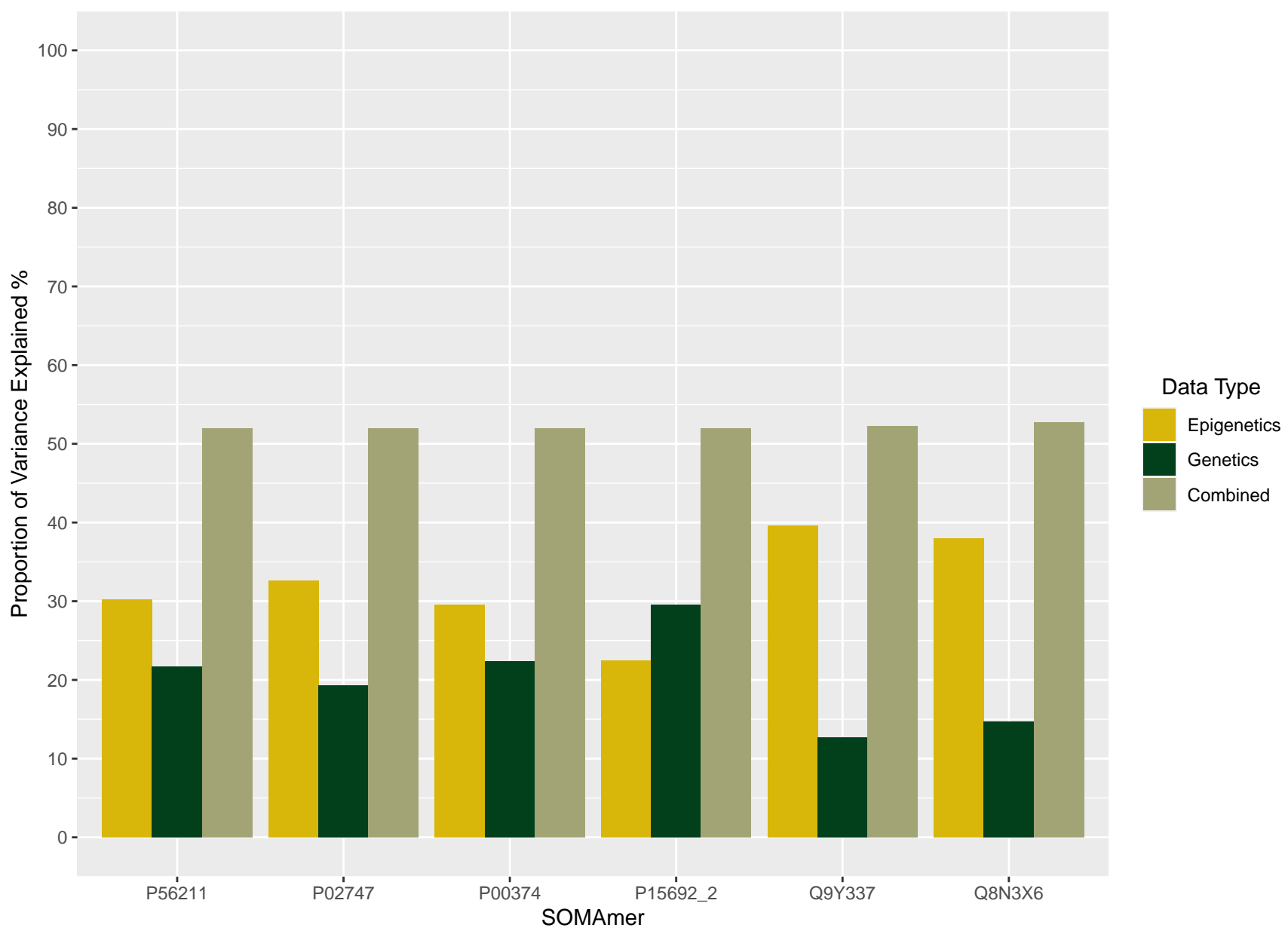

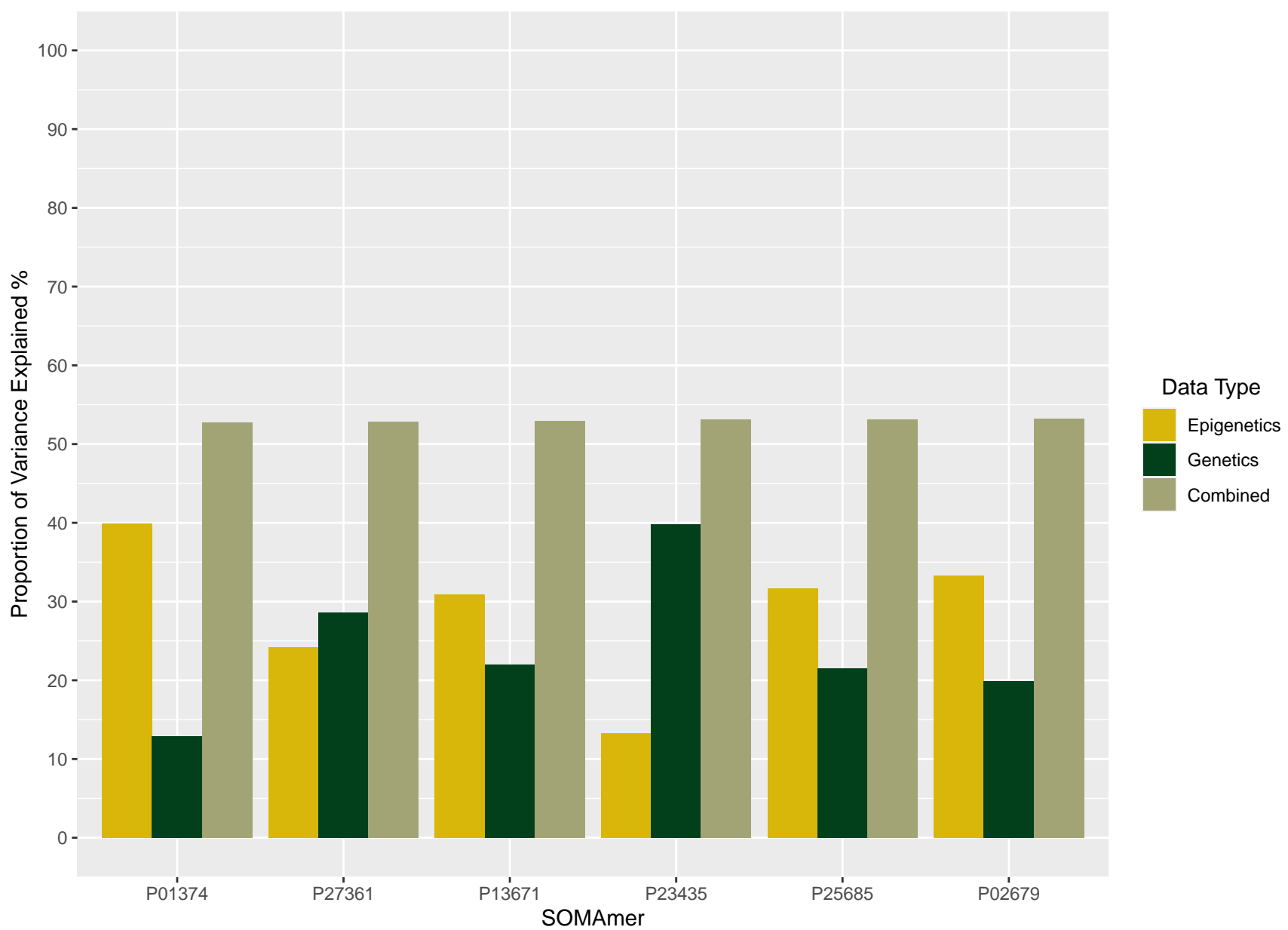

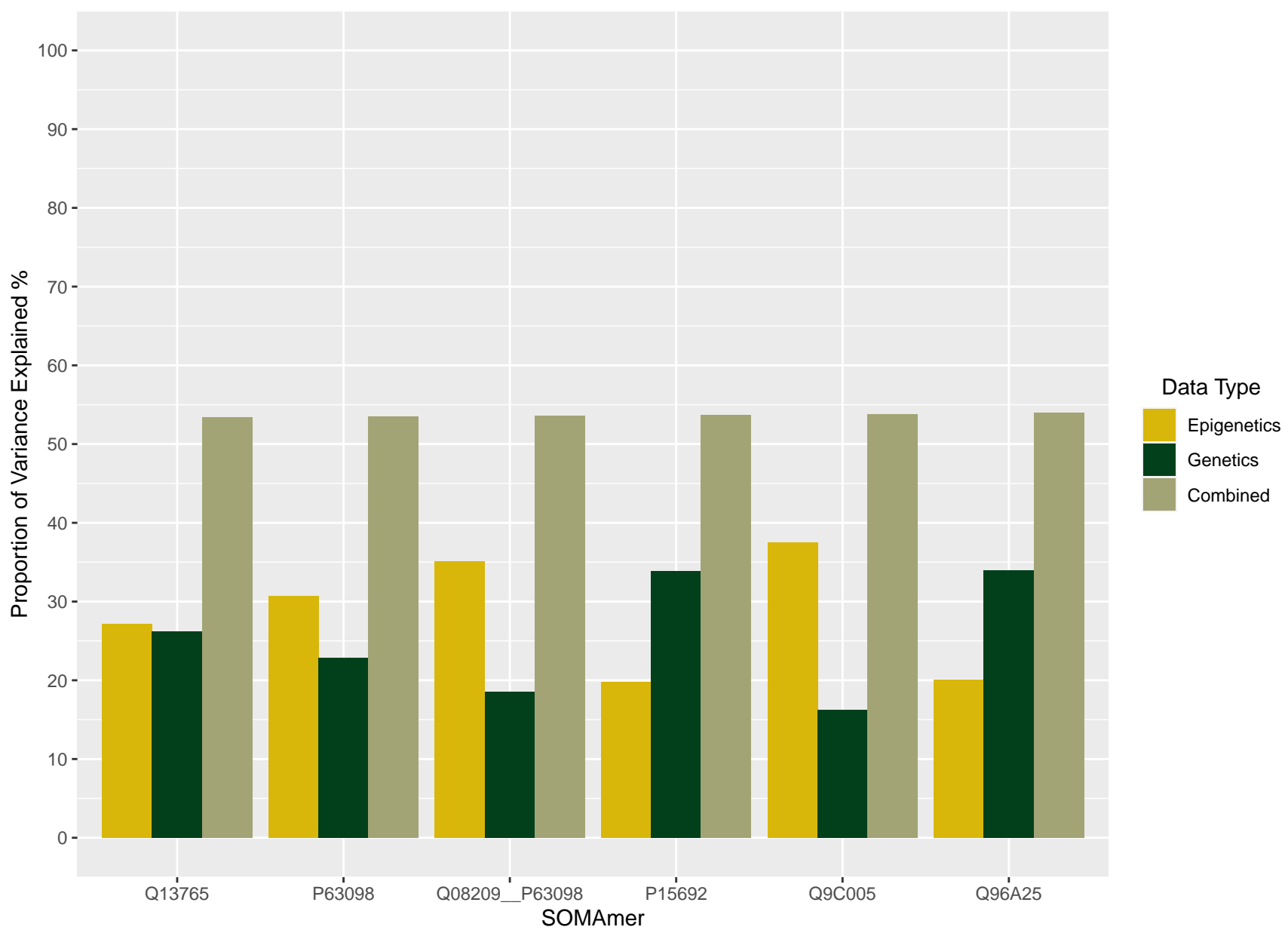

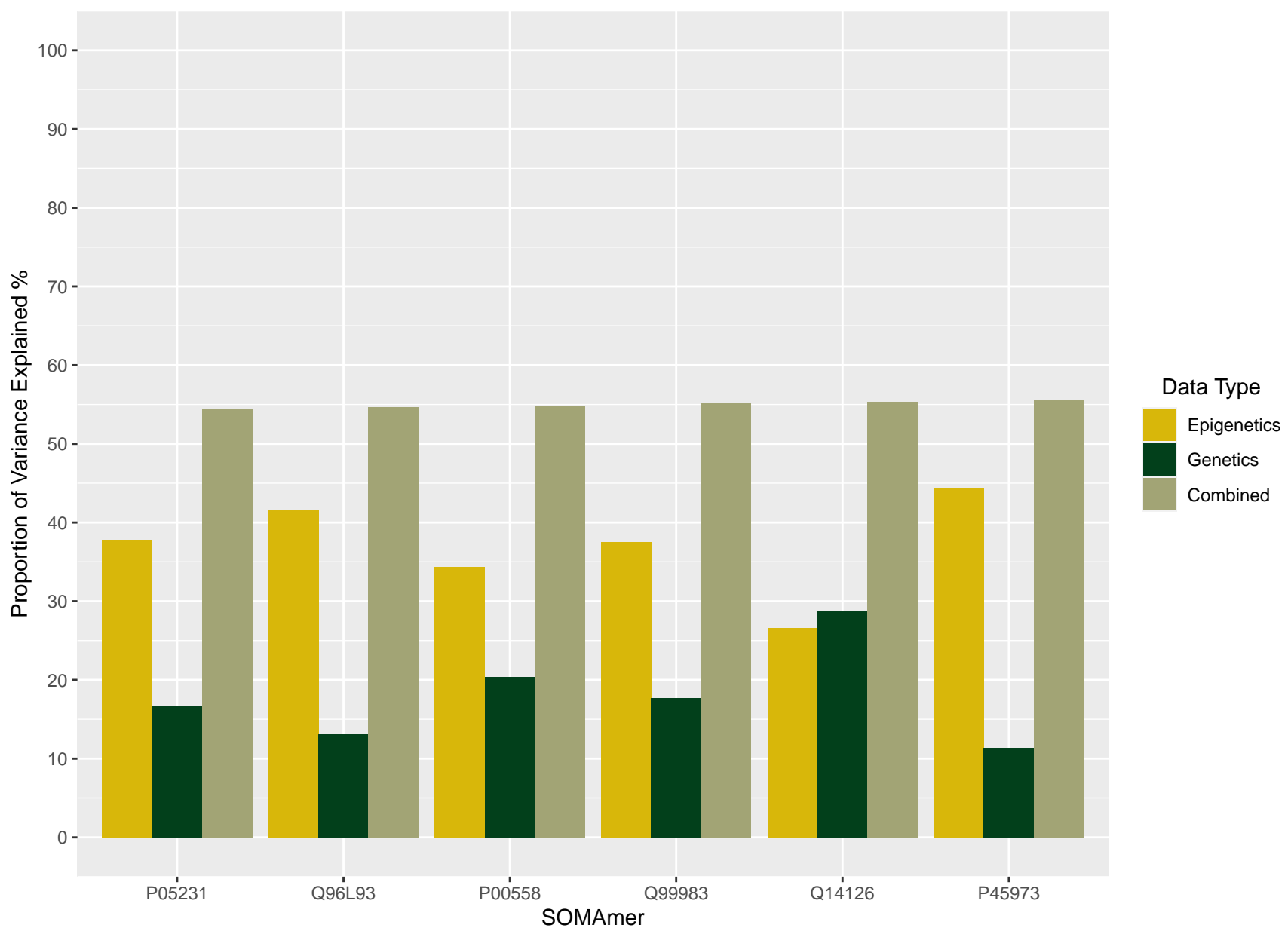

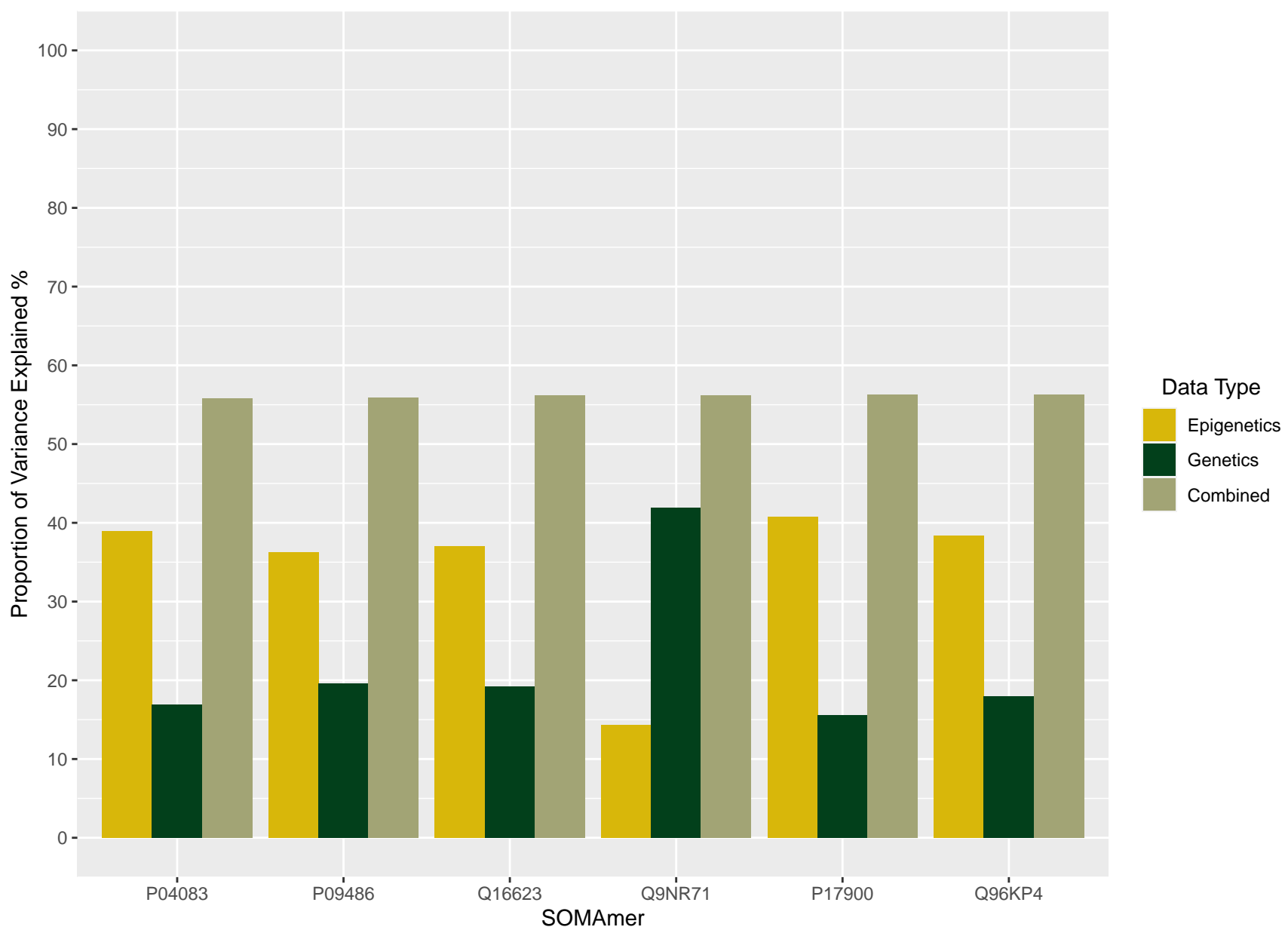

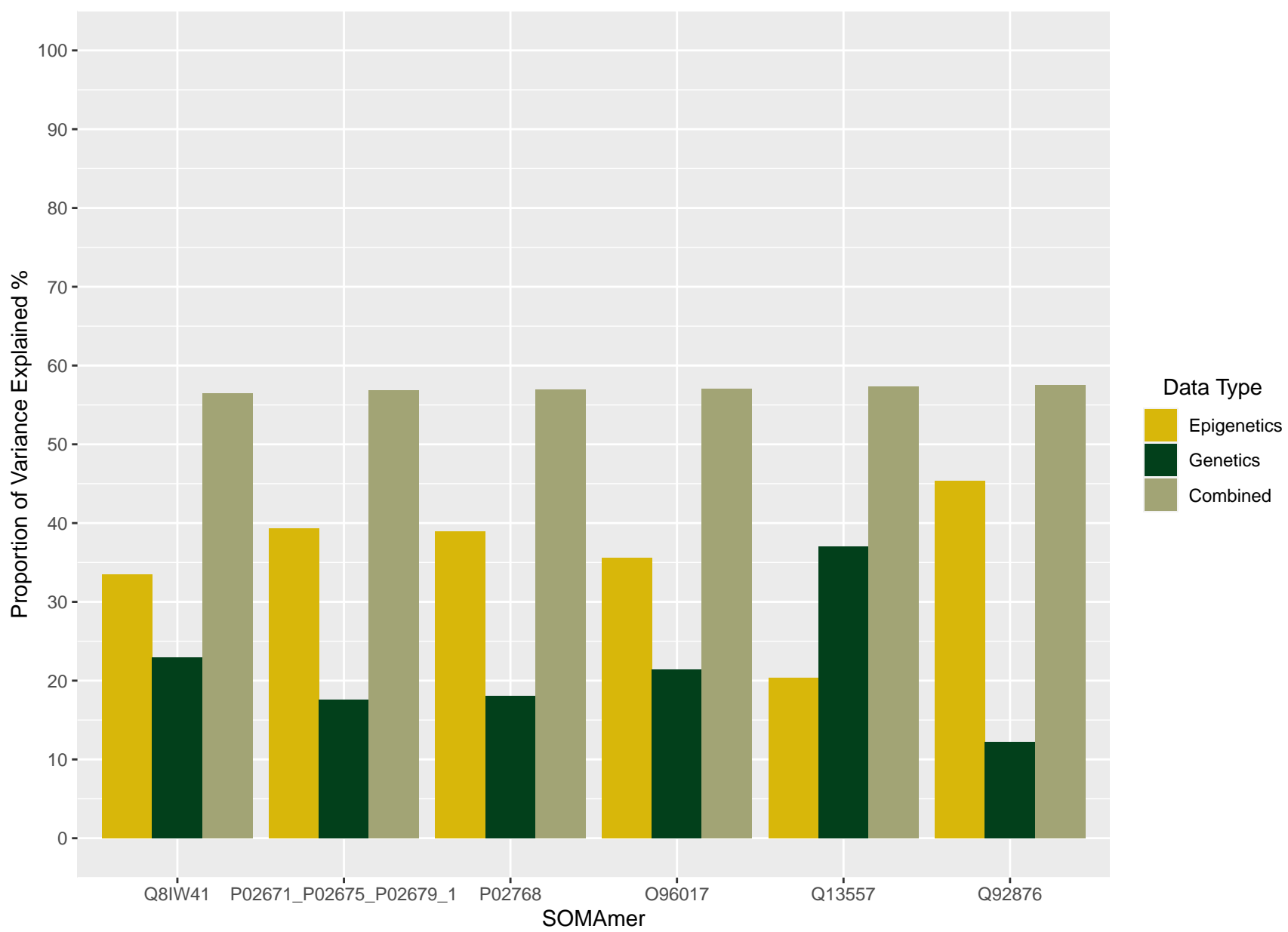

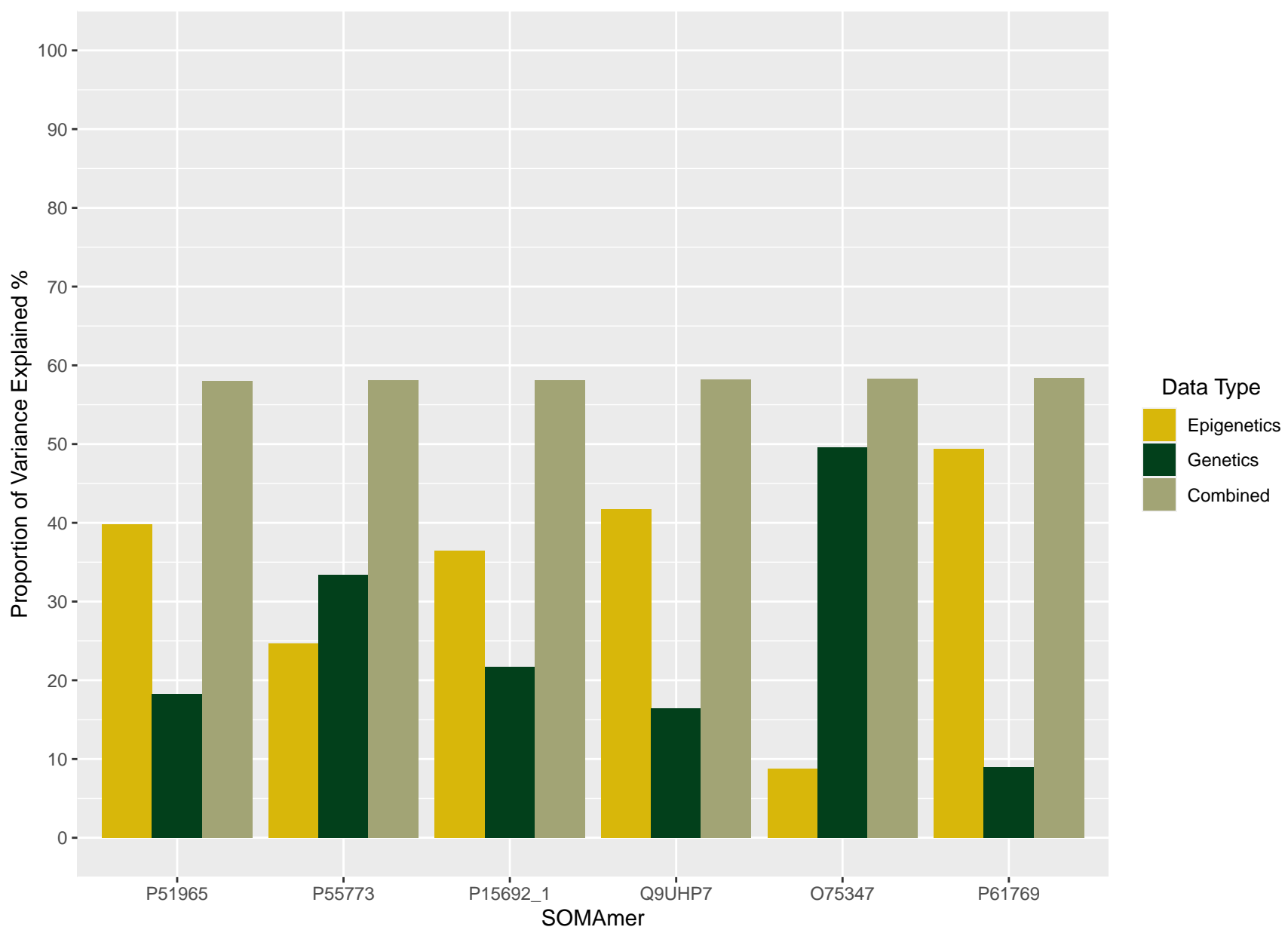

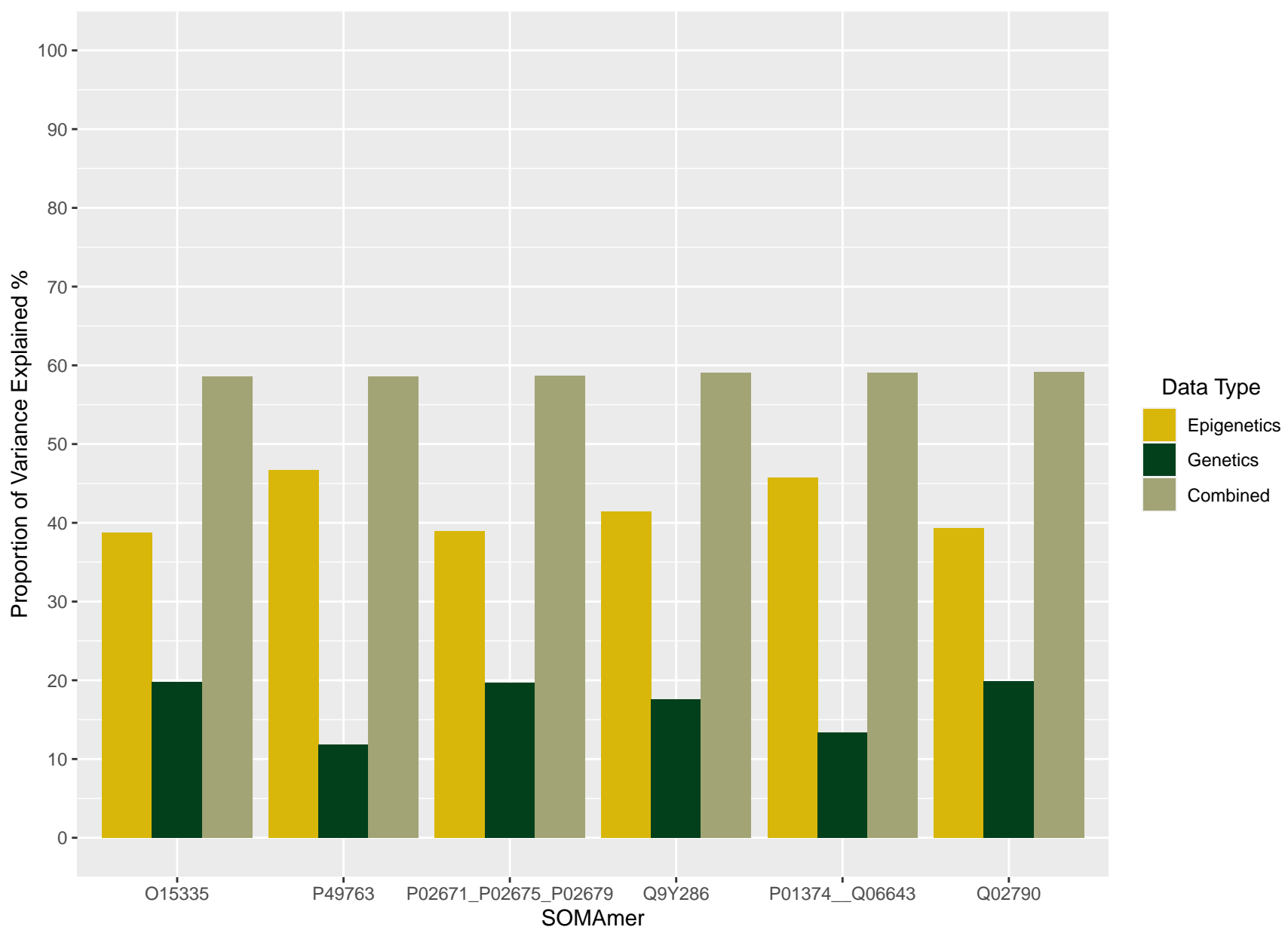

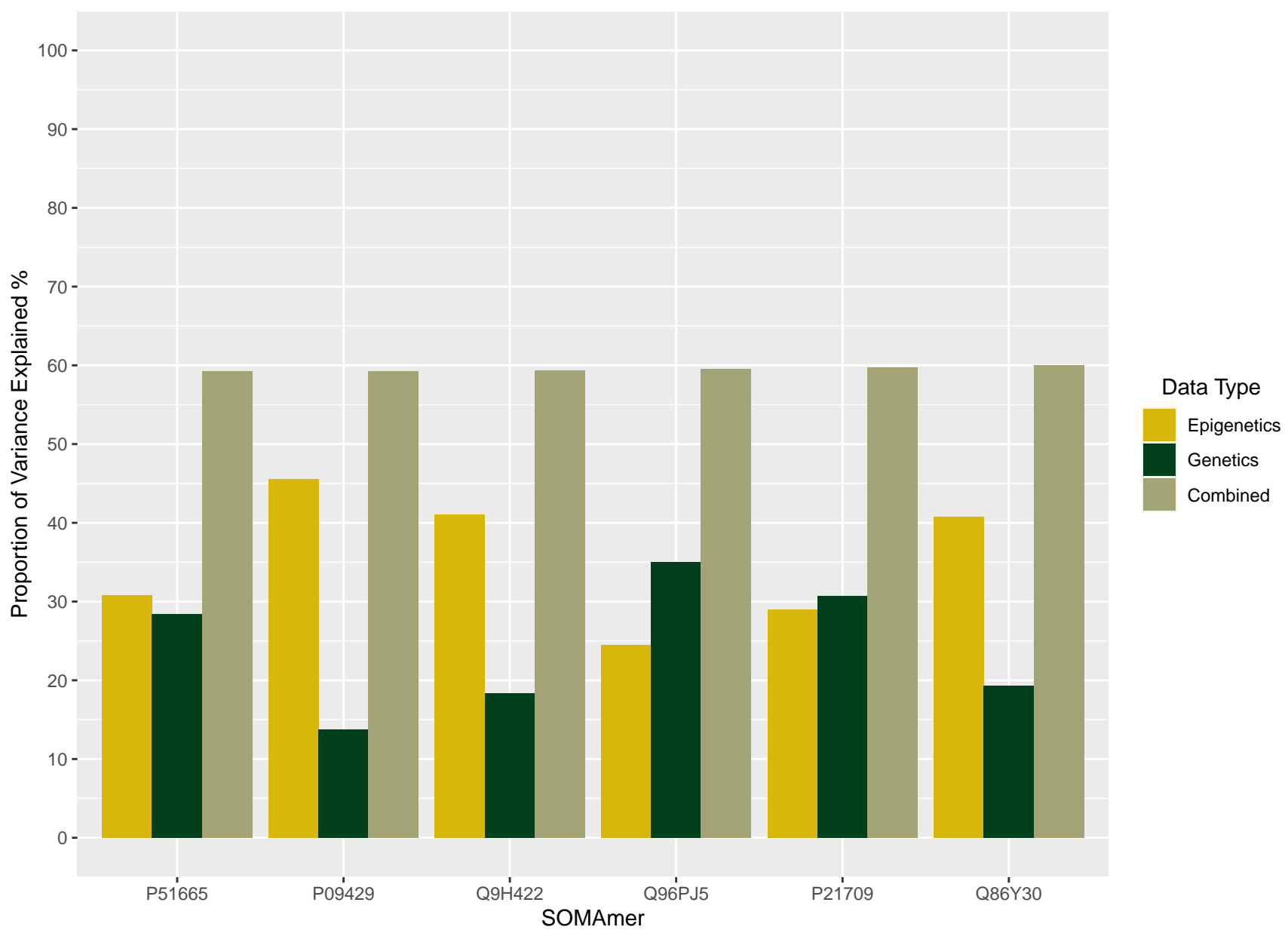

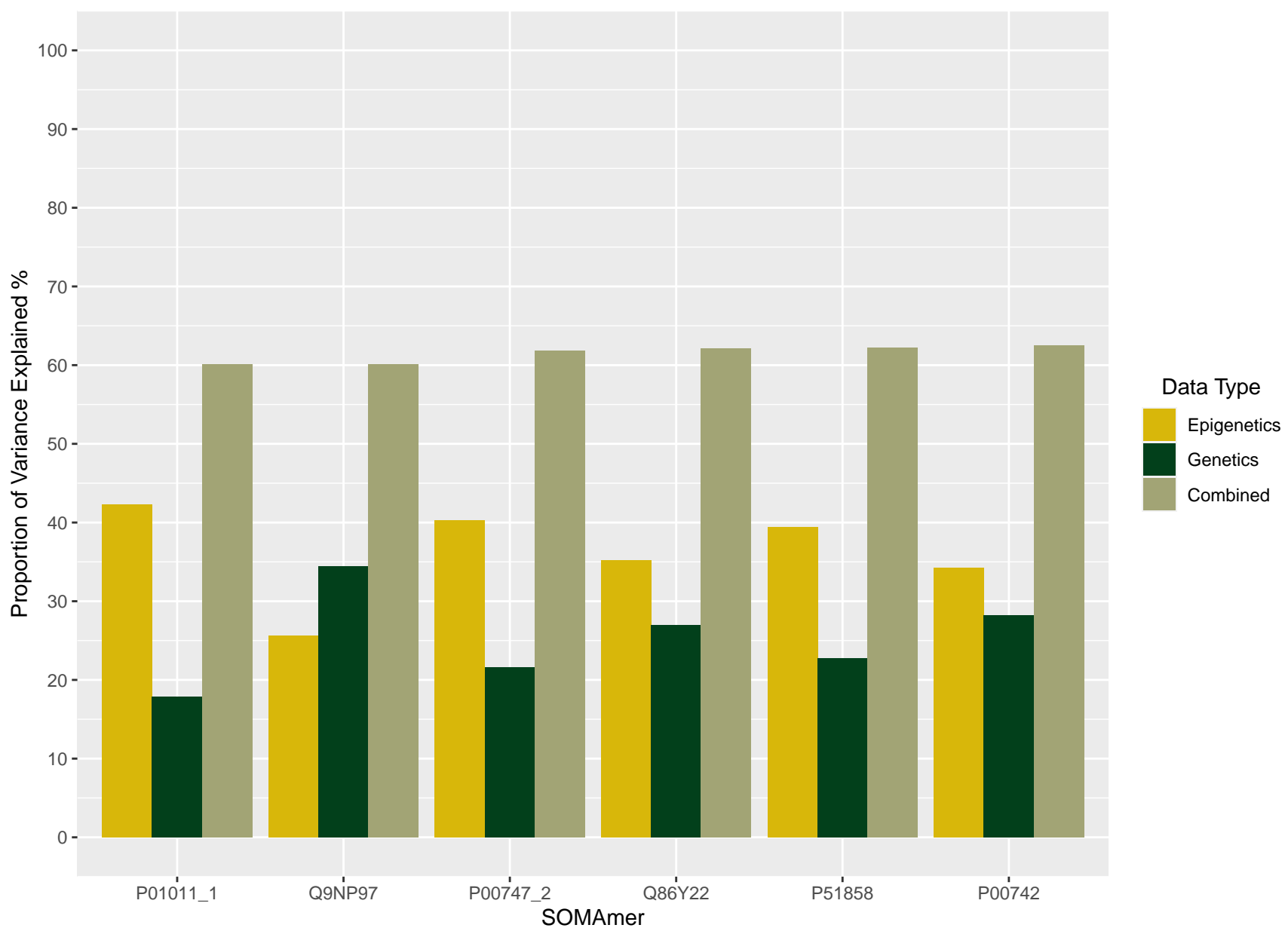

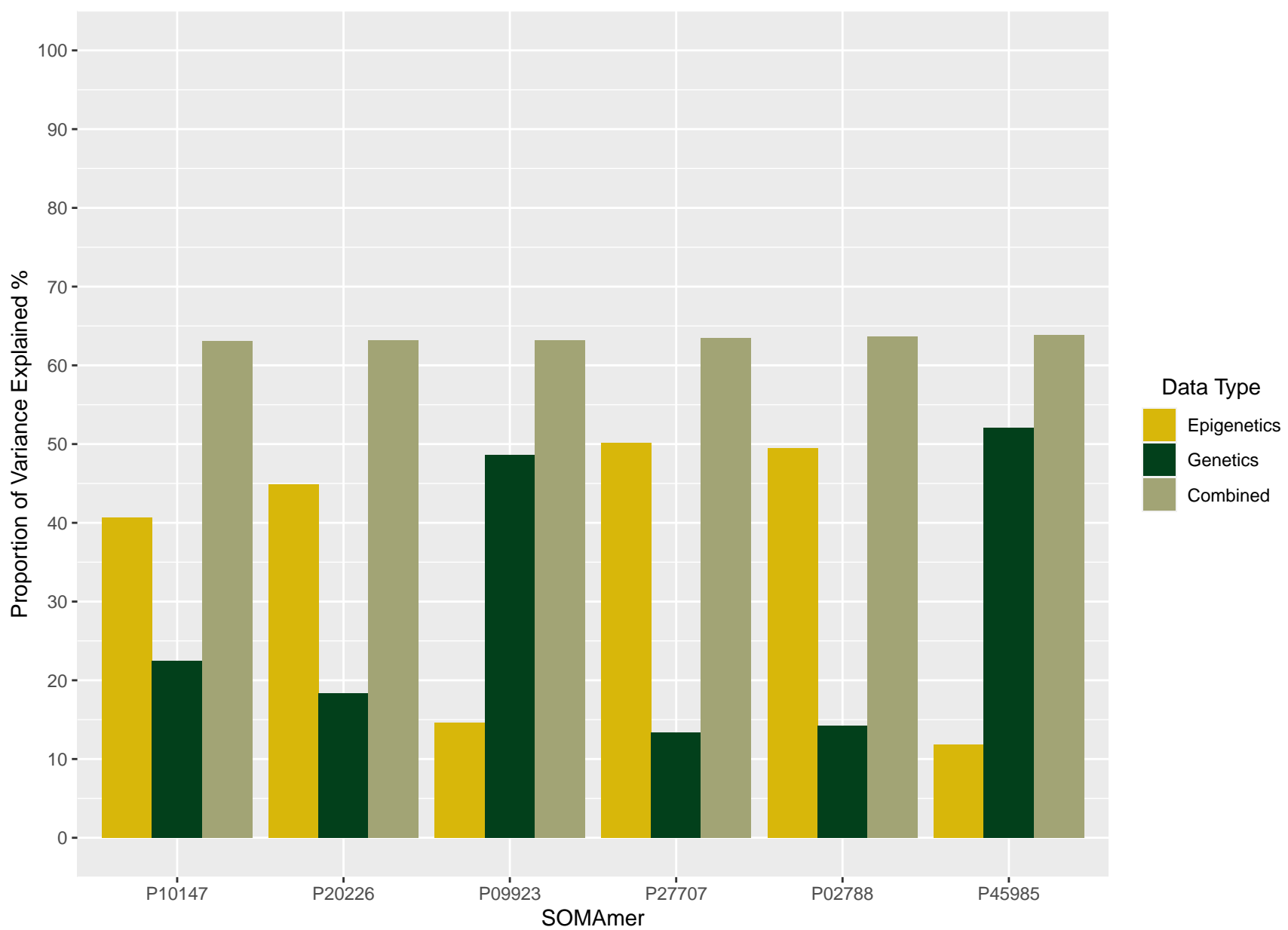

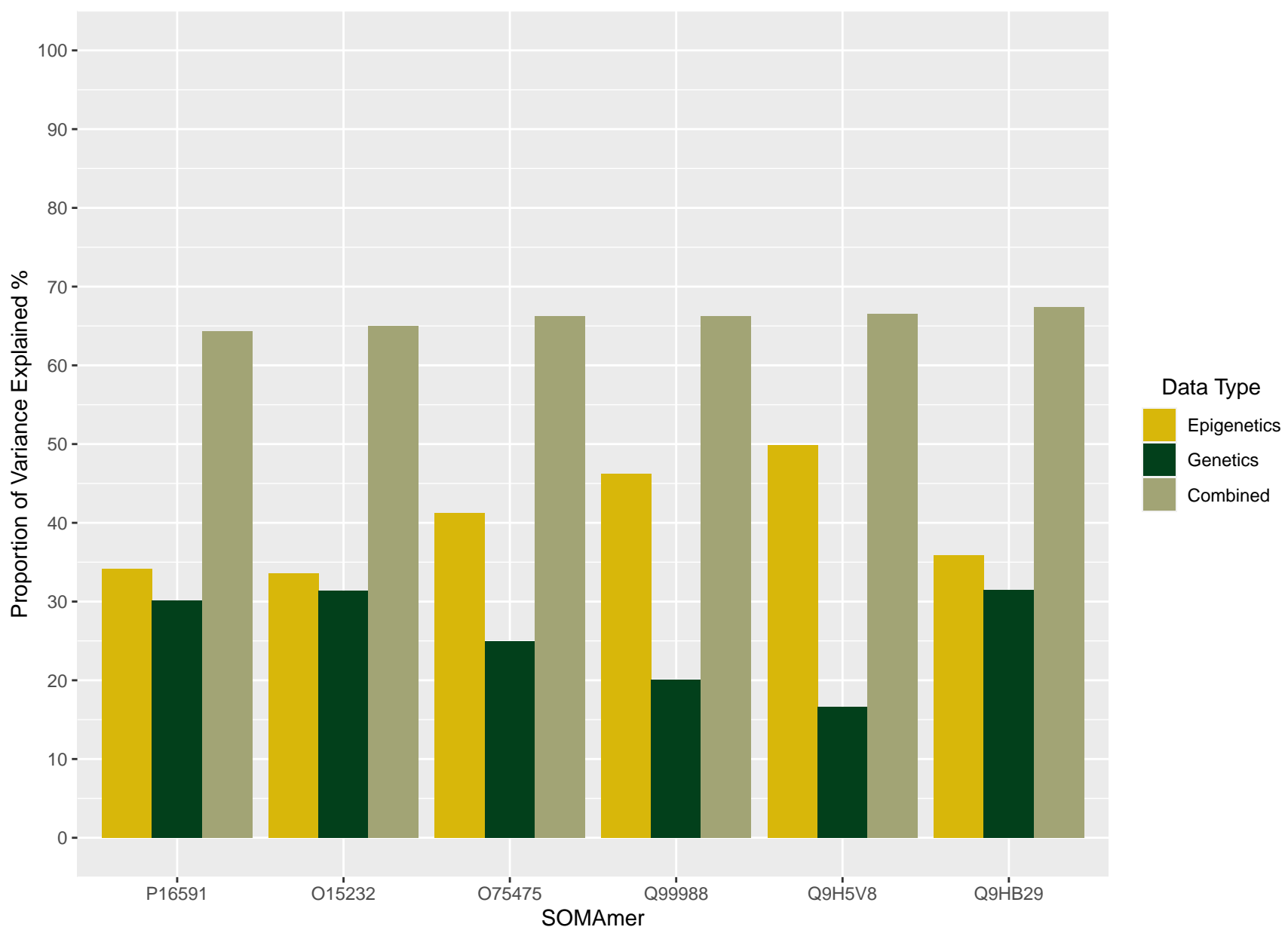

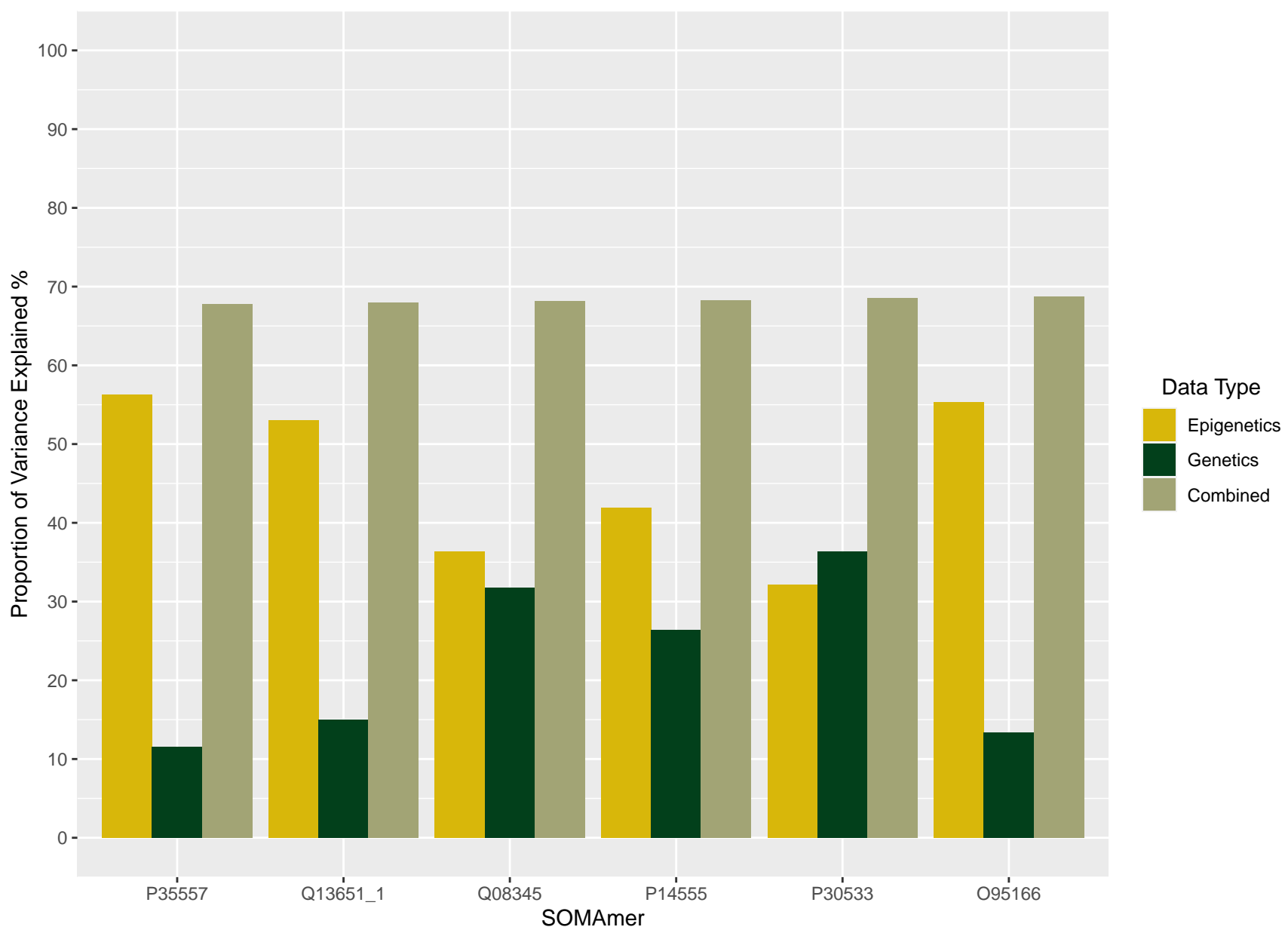

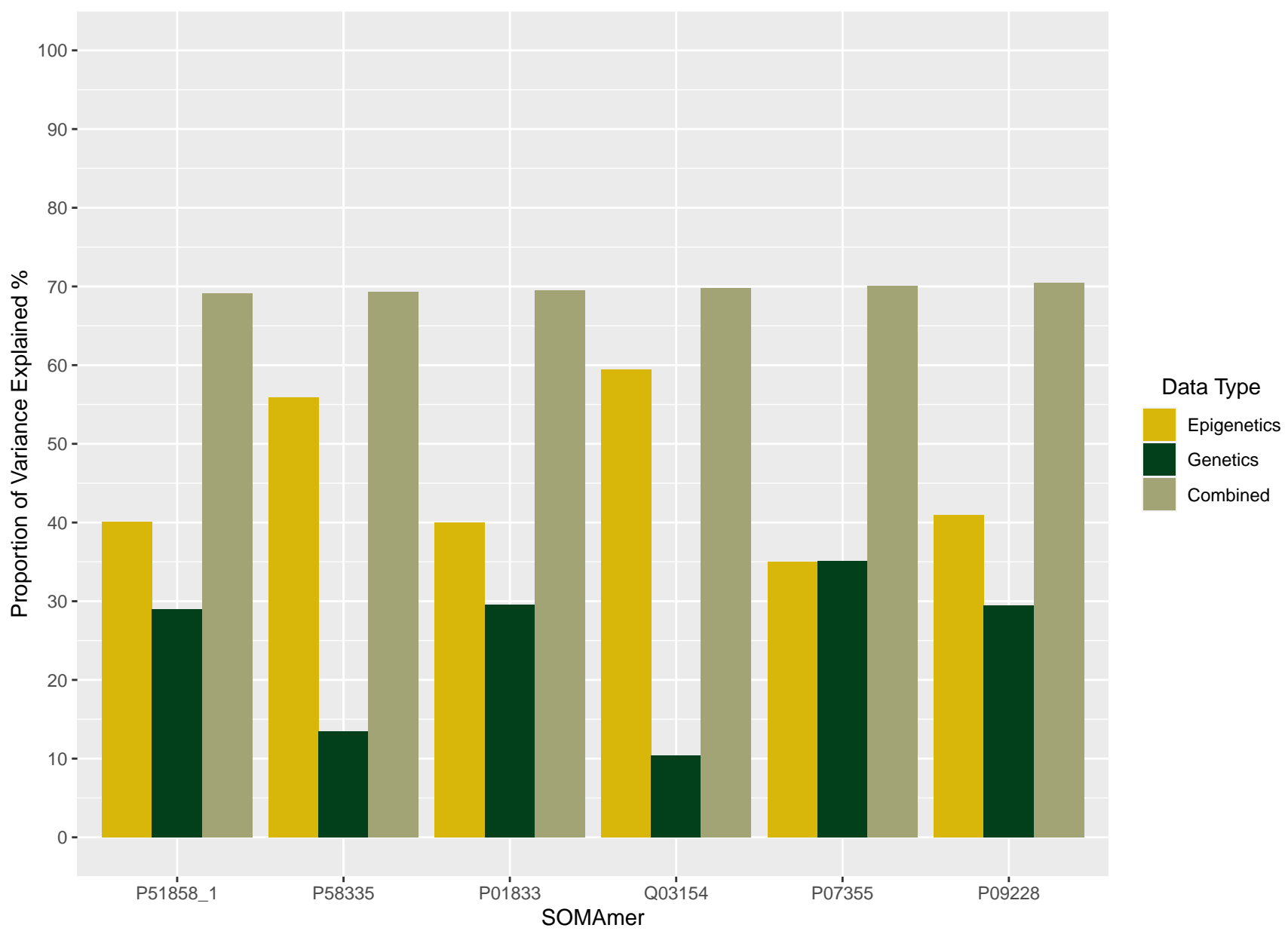

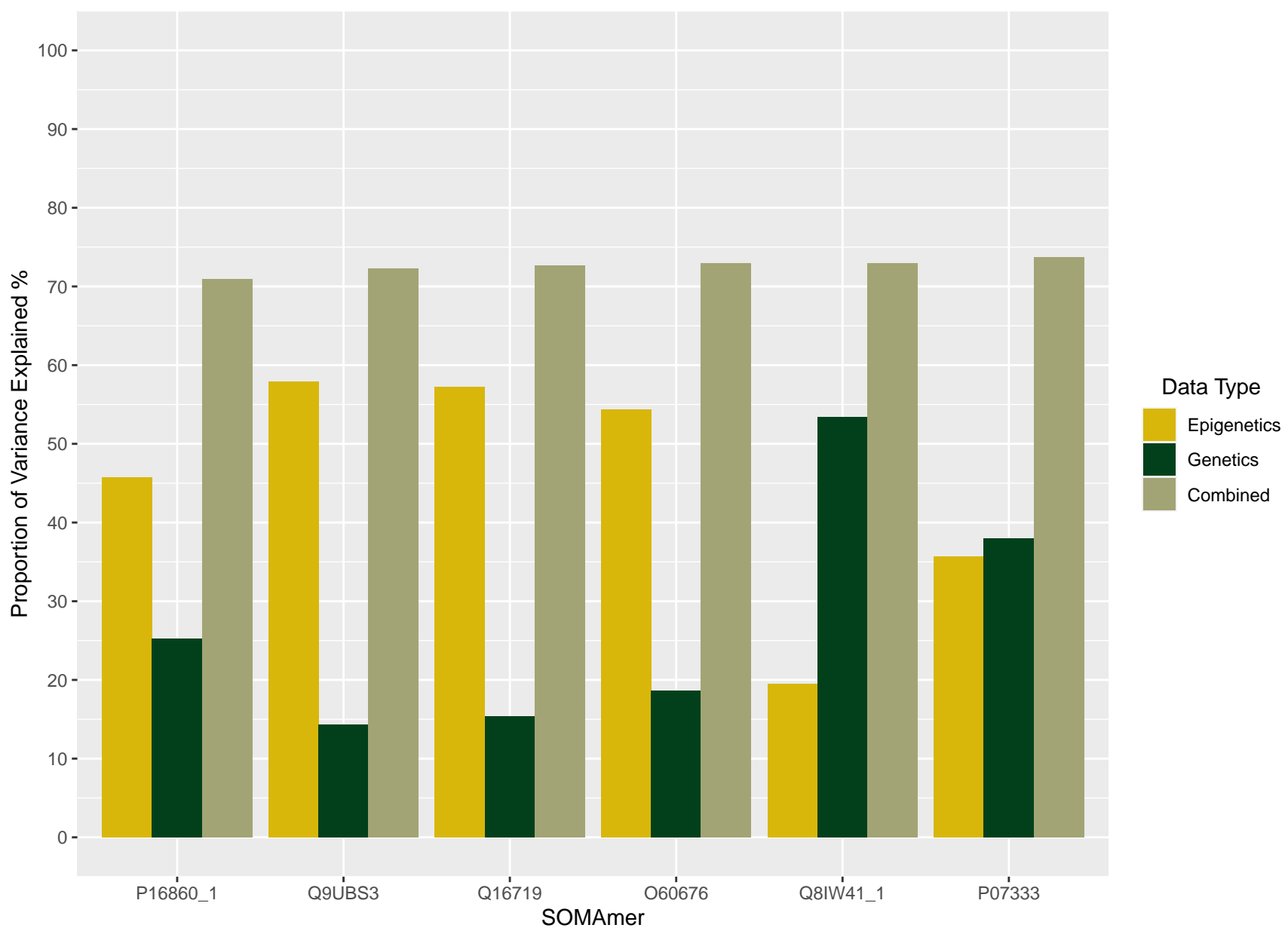

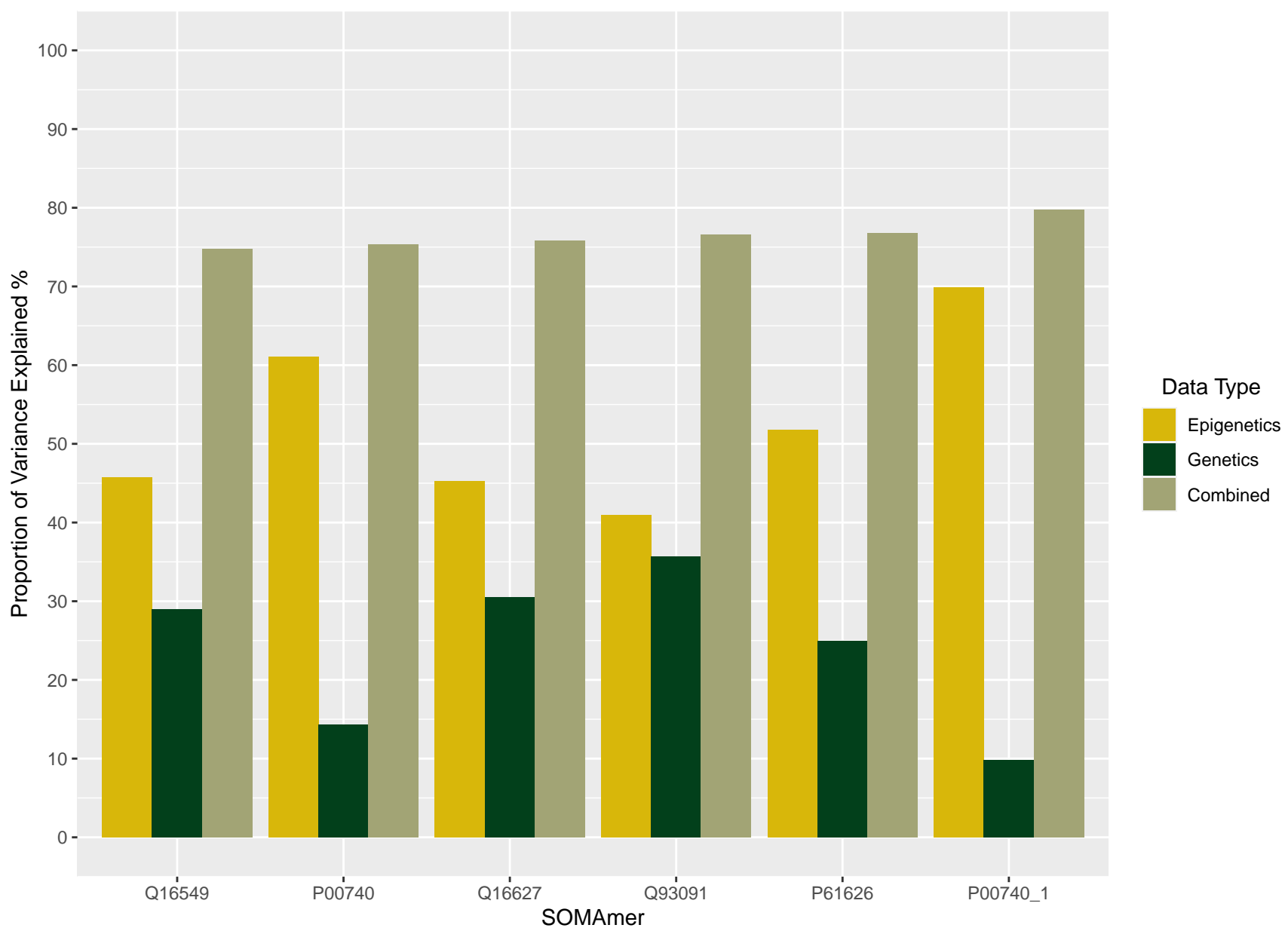

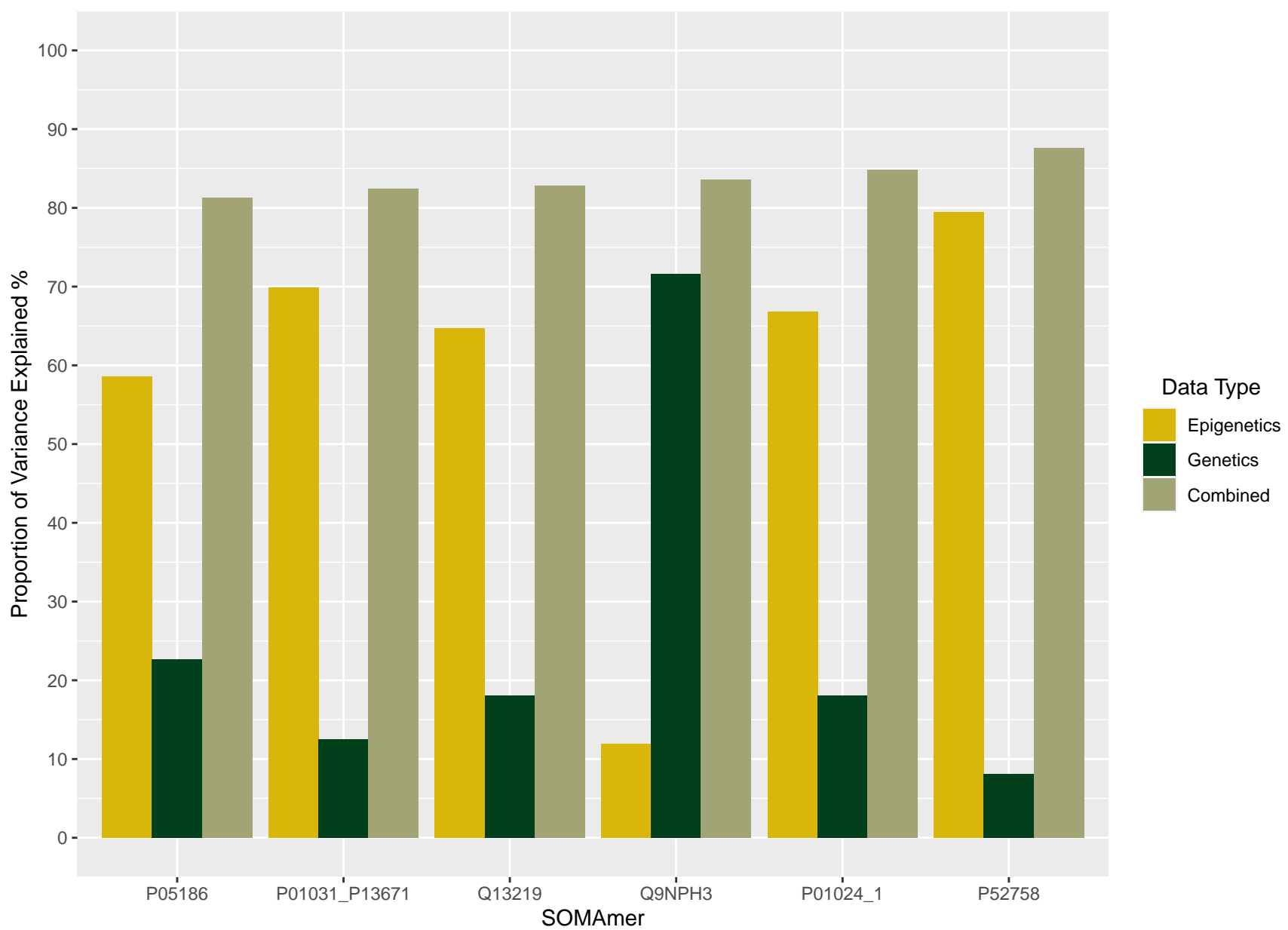

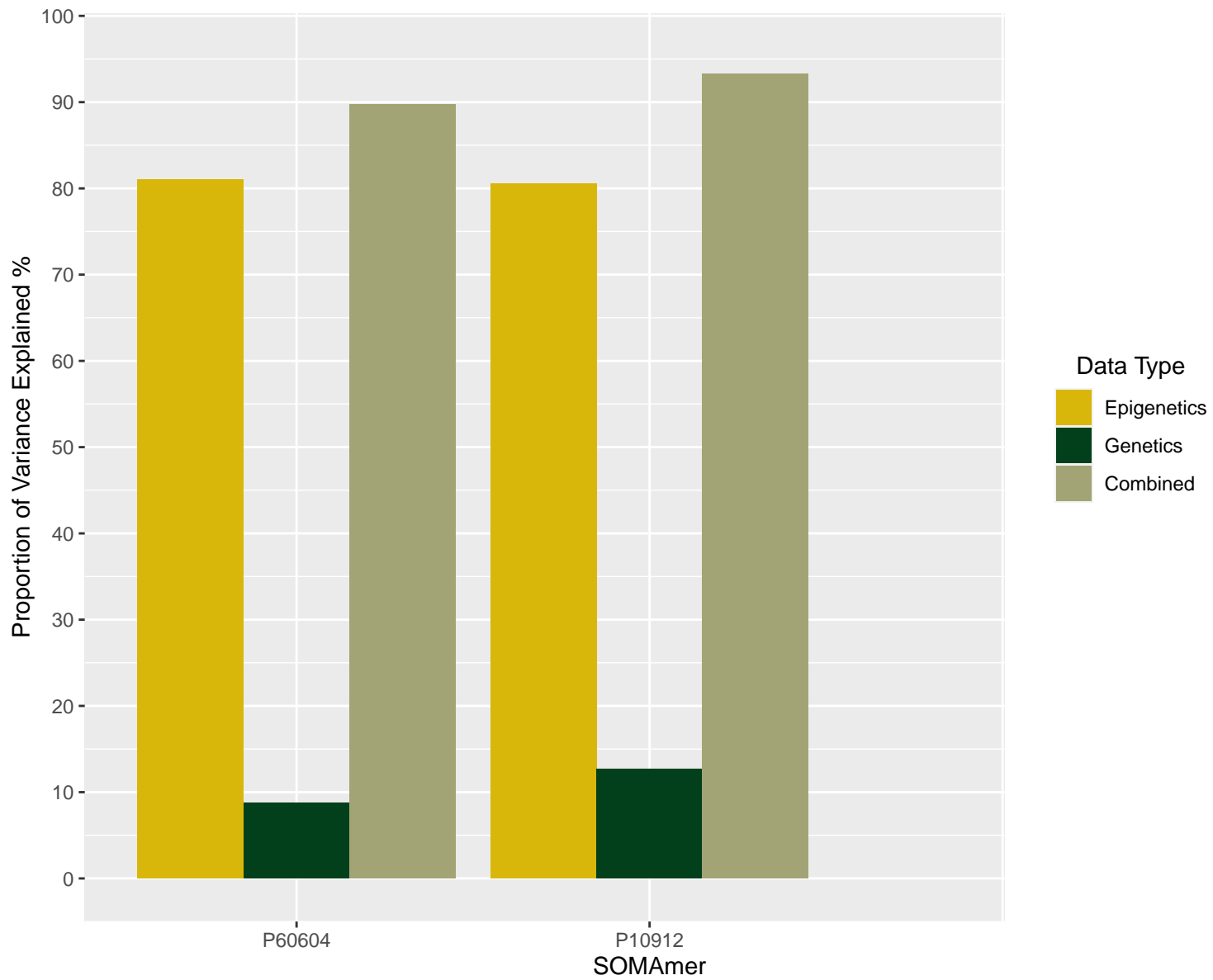

Supplement: Supplementary file 3 — SUPPORTING INFORMATION [file DAD2-14-e12280-s004.pdf]
